# Genotoxicants Target Distinct Molecular Networks in Neonatal Neurons

**DOI:** 10.1289/ehp.9073

**Published:** 2006-09-07

**Authors:** Glen E. Kisby, Antoinette Olivas, Melissa Standley, Xinfang Lu, Patrick Pattee, Jean O’Malley, Xiaorong Li, Juan Muniz, Srinavasa R. Nagalla

**Affiliations:** 1 Center for Research on Occupational and Environmental Toxicology (CROET), Oregon Health & Science University, Portland, Oregon; 2 Department of Pediatrics, School of Medicine, Oregon Health & Science University, Portland, Oregon

**Keywords:** cerebellum, DNA damage, granule cell, HN2, MAM, methylazoxymethanol, nitrogen mustard

## Abstract

**Background:**

Exposure of the brain to environmental agents during critical periods of neuronal development is considered a key factor underlying many neurologic disorders.

**Objectives:**

In this study we examined the influence of genotoxicants on cerebellar function during early development by measuring global gene expression changes.

**Methods:**

We measured global gene expression in immature cerebellar neurons (i.e., granule cells) after treatment with two distinct alkylating agents, methylazoxymethanol (MAM) and nitrogen mustard (HN2). Granule cell cultures were treated for 24 hr with MAM (10–1,000 μM) or HN2 (0.1–20 μM) and examined for cell viability, DNA damage, and markers of apoptosis.

**Results:**

Neuronal viability was significantly reduced (*p* < 0.01) at concentrations > 500 μM for MAM and > 1.0 μM for HN2; this correlated with an increase in both DNA damage and markers of apoptosis. Neuronal cultures treated with sublethal concentrations of MAM (100 μM) or HN2 (1.0 μM) were then examined for gene expression using large-scale mouse cDNA microarrays (27,648). Gene expression results revealed that *a*) global gene expression was predominantly up-regulated by both genotoxicants; *b*) the number of down-regulated genes was approximately 3-fold greater for HN2 than for MAM; and *c*) distinct classes of molecules were influenced by MAM (i.e, neuronal differentiation, the stress and immune response, and signal transduction) and HN2 (i.e, protein synthesis and apoptosis).

**Conclusions:**

These studies demonstrate that individual genotoxicants induce distinct gene expression signatures. Further study of these molecular networks may explain the variable response of the developing brain to different types of environmental genotoxicants.

The Children’s Health Act (2000) authorized the National Children’s Study (NCS) to study the long-term effects of the environment on children’s health and development by examining children across the United States from before birth to 21 years of age (Branum et al. 2003). One of the top priorities of the NCS was to identify factors responsible for the increasing rise of neurodevelopmental disorders (e.g, learning disabilities, mental retardation, attention deficit disorder) (Branum et al. 2003). Because brain development begins early in fetal life and continues until adolescence, exposure to environmental chemicals at this early age may be a leading cause of neuro-developmental disorders. In support, a report by the National Research Council recently concluded that 3% of developmental disabilities are the direct consequence of exposure to environmental neurotoxins and that another 25% arise out of the interplay between environmental factors and genetic susceptibility (Landrigan et al. 2004). These conclusions were derived from data collected on children who had been exposed to established neurotoxic agents (e.g., alcohol, pesticides, heavy metals, polychlorinated biphenyls). However, many of the chemicals identified by the Chemical Agents Working Group of the NCS are genotoxicants and therefore are capable of directly or indirectly damaging DNA to induce long-term neurologic impairment. Although DNA damage is a characteristic feature of certain neurodevelopmental disorders (Nishioka and Arnold 2004) or neurologic disease (Alam et al. 1997; Lyras et al. 1997; Mecocci et al. 1994, 1997), our understanding of how genotoxicants may contribute to these conditions is poorly understood.

The complex and hierarchical cytoarchitecture of the mature brain is the culmination of a sequence of biochemical and molecular events tightly controlled by specific patterns of gene expression. Regions of the central nervous system (CNS) develop at different stages and this correlates with a distinct sequence of events that includes cell proliferation, migration, and differentiation or maturation. Interference at any one of these stages of development would be expected to induce permanent impairment. Because most neurodevelopmental disorders are categorized as migrational disorders (Gleeson 2001), environmental agents that preferentially target the DNA of immature postmitotic neurons would be expected to disrupt the transcriptional events that control the key steps involved in laying down the final cytoarchitecture of the mature brain. Identifying the key molecular networks specifically targeted by genotoxicants in immature postmitotic neurons could provide an important first step in understanding how this class of environmental agents influences brain development.

Methylazoxymethanol (MAM) and nitrogen mustard (HN2) are two established genotoxicants that reproducibly disrupt neuronal development when administered during the fetal or neonatal period of CNS development (Cattabeni and Di Luca 1997; Ferguson 1996; Graef et al. 1948; McDonald and Asano 1961). The glucoside form of MAM (i.e, cycasin) is also strongly linked to a prototypical neurologic disorder found in the western Pacific with features of amyotrophic lateral sclerosis, Parkinson disease and an Alzheimer-like dementia (ALS/PDC; Spencer et al. 1991; Zhang et al. 1996). These studies suggest that early life exposure to a genotoxicant is associated with neurodevelopmental or neurodegenerative changes. The genotoxic properties of MAM have been widely used by neurobiologists to selectively target neurons during CNS development (Cattabeni and Di Luca 1997; Colacitti et al. 1999), whereas the chemotherapeutic agent HN2 induces immediate and delayed neurotoxicity in humans (Sullivan et al. 1982) and is a potent experimental teratogen [Sullivan et al. 1982; see also review by Spencer et al. (1999)]. Rodents treated with MAM or HN2 *in utero* or within 1–5 days of birth show strikingly abnormal development of the cerebral cortex (Balduini et al. 1986; Cattabeni and Di Luca 1997; Ferguson and Holson 1997) or cerebellum (Ferguson et al. 1996; Sullivan-Jones et al. 1994), respectively, and exhibit changes in motor or cognitive function. Prenatal exposure to MAM is characterized by cortical atrophy (Colacitti et al. 1999), an increased susceptibility to epileptogenic agents (Baraban and Schwartzkroin 1996; Chevassus-Au-Louis et al. 1999; DeFeo et al. 1995; Jacobs et al. 1999), an age-dependent decline in learning and memory (Matijasevic et al. 1993; Vorhees et al. 1984), and an impaired social behavior that bears resemblance to that seen in schizophrenia (Flagstad et al. 2005; Talamini et al. 1998, 1999). When MAM is administered after birth (1–4 days), the effects are confined primarily to the cerebellum (Ferguson 1996; Sullivan-Jones et al. 1994). This exposure also leads to atrophy that is characterized by specific targeting of glutaminergic and GABAergic precursor cells of the cerebellum (especially granule cells) resulting in misalignment of Purkinje cells and ectopic and multinucleated granule cells. Multinucleated and ectopic neurons have also been reported in the cerebellum and vestibular nuclei of subjects with ALS/PDC (Shiraki and Yase 1975), an observation that suggests human exposure to MAM during early CNS development may have arrested the mitotic and migratory developmental responses of neurons.

Gene expression profiling is becoming an increasingly useful approach for elucidating complex relationships between toxins and the patterns of plasticity during CNS development (Mody et al. 2001; Poguet et al. 2003) or for understanding the full impact of environmental toxins on cells or tissues (Amin et al. 2002; Mandel et al. 2002). For example, gene expression profiling has been used recently to dissect the complex mechanisms underlying CNS injury in several neurodevelopmental disorders (e.g., epilepsy, schizophrenia, learning disabilities) (Becker et al. 2002; Mirnics et al. 2000) and in neurodegenerative disease (Ishigaki et al. 2002; Pasinetti 2001). Because the majority of neurodevelopmental disorders in children occur during the migration of immature neurons, gene expression profiling was used to identify the specific molecular networks targeted by MAM or the related alkylating agent HN2 in cultures of young postmitotic cerebellar neurons.

## Materials and Methods

### Neuronal and astrocyte cell cultures

We prepared primary mouse granule and astrocyte cell cultures from the cerebella of 6- to 8-day-old neonatal C57BL/6 (Charles River Laboratories, Wilmington, MA) mice by placing the tissues in ice-cold Hibernate/B27 cell culture media (Invitrogen Corp., Carlsbad, CA) and dissociating the tissue in balanced salt solution with 0.1% trypsin as previously described (Kisby et al. 2000, 2004; Meira et al. 2001). The cell suspension was placed in poly-d-lysine coated (Biocoat; BD Biosciences, Bedford, MA) 48-well plates (viability studies), 8-well chamber slides [terminal deoxynucleotidyl transferase-mediated biotinylated-UTP nick end-labeling (TUNEL)], or 6-well plates (DNA damage) at a density of 0.07 × 10^6^/well (8-well chamber slides and 48-well plates) or 1 × 10^6^ cells/well (6-well plates), respectively. We fed cell cultures weekly by adding fresh culture media to the wells and maintained the cells for 7 days (neurons) or 3–4 weeks (astrocytes) before treatment with 10–1,000 μM MAM or 0.1–20 μM mechlorethamine hydrochloride (HN2). All animals used in these studies were treated humanely and with regard to the alleviation of suffering according to protocols approved by the Oregon Health & Science University Institutional Animal Care and Use Committee.

### Cell viability

Mouse neuronal and astrocyte cell cultures treated with control media or media supplemented with various concentrations of MAM or HN2 were examined for cell viability using the fluorochrome acetoxymethyl ester, as previously described (Kisby et al. 2004; Meira et al. 2001). The fluorochrome-containing media was aspirated, the cultures washed once with control media, and cell survival examined on a fluorescence microplate reader (GeminiXS; Molecular Devices, Sunnyvale, CA) with well-scan capabilities. Values were expressed as the mean percent surviving of control cells ± SE (*n* = 6/treatment group × 3–5 separate experiments).

### DNA damage

#### *N*7-Alkylguanine levels

We isolated and purified DNA from MAM- or HN2-treated cerebellar neuronal cell cultures by extracting the tissue with Tri-Reagent (Molecular Research Corp., Cincinnati, OH) according to the manufacturer’s instructions. DNA concentration ranged between 20 and 30 μg/1 × 10^6^ cells, and the purity was checked by measuring 260/280 ratios (range 1.7–2.0). An alkylated DNA standard was prepared by treating calf thymus DNA (CT-DNA) with 1 mM MAM in buffer [300 mM MOPS, 2 mM EDTA (pH 7.5)] for 1 hr at 37°C. DNA samples and alkylated CT-DNA were depurinated by incubating in 0.1 N HCl for 17 hr at 37°C. The depurinated samples and standards were neutralized with 1.0 N NaOH, passed through a C_18_ SepPak cartridge (Millipore Corp., Bedford, MA), and taken to dryness in a speed-vac. The lyophylized samples and alkylated DNA were analyzed for *N*7-methylguanine (N7-mG) or *N*7-alkylguanine [i.e, *N*-(2-hydroxyethyl)-*N*-(2-(7-guaninyl)ethyl)-methylamine (GMOH)] DNA lesions by HPLC with electrochemical detection as previously described by Eizirik and Kisby (1995), Esclaire et al. (1999), and Kisby et al. (2000). Alkylated DNA was used to determine recovery (> 90%) of N7-mG and GMOH from the extraction process. N7-mG and GMOH levels were determined in samples and from a standard curve (*r* = 0.99) of CT-DNA alkylated with MAM or HN2, respectively. Values are expressed as fmoles N7-mG or GMOH per microgram DNA.

#### TUNEL labeling

Primary cerebellar neuronal cultures treated for 24 hr with MAM or HN2 were examined for DNA fragmentation using TUNEL with the NeuroTacs staining kit according to the manufacturer’s instructions (Trevigen, Gaithersburg, MD). After toxin treatment, the cells were fixed with 4% buffered paraformaldehyde, and the incorporation of biotinylated nucleotides was visualized by incubating the cells with NovaRed (Vector Labs, Inc, Burlingame, CA). Slides were lightly counterstained with methyl green and the cells examined by light microscopy as previously described (Kisby et al. 2004).

### Microarrays

We purchased 27,648 sequence-verified mouse cDNA clones from Research Genetics [Brain Molecular Anatomy Project (BMAP) clones; Invitrogen Corp.] and The National Institute of Aging (NIA clones; Bethesda, MD) as frozen bacterial stocks were used to create two individual arrays (13,824 genes/array) spanning nearly the entire mouse genome. Universal forward and reverse primers were amino modified with a 5′ C_12_ spacer. Polymerase chain reaction (PCR) products were purified using Telechem PCR clean-up plates, dried down, and resuspended in 20 μL Telechem spotting solution and printed on TeleChem SuperAldehyde substrates using a Cartesian Pixsys printer with quill pins from TeleChem International (Sunnyvale, CA).

#### RNA preparation

We isolated RNA from cerebellar neuronal cultures treated for 24 hr with 100 μM MAM or 1.0 μM HN2 using Tri-Reagent (Molecular Research Corp.) according to the manufacturer’s protocol. Because RNA concentrations were low (10–15 μg/well for 6-well plate), two wells were combined, and each combined sample (*n* = 3) was analyzed for gene expression using mouse cDNA microarrays. We used bromochloropropane for the initial phase separation. RNA was dissolved in water that had been treated with diethyl pyrocarbonate to ensure that it was RNAse free and quantitated based on optical density (OD)_260_.

#### Gene arrays processing

RNA (10 μg) was reverse transcribed using 2 μg of oligo dT primer (24mer) in the presence of 200 mM dNTP mixture (dATP, dGTP, dCTP), 100 mM dTTP, 100 mM 5-(3-aminoallyl)-2′-deoxyuridine-5′-triphosphate (Sigma Chemical Corp., St. Louis, MO) and 300 U of Superscript II (Invitrogen, Carlsbad, CA) to generate aminoallyl-modified cDNA probes. After hydrolysis of the original RNA, we used a Qiagen PCR cleanup kit (Qiagen, Valencia, CA) with a modified protocol to purify the cDNA product. The cDNA probe was then dried down and resuspended in 0.1 M NaCO_2_ buffer (pH 9.0) and coupled to *N*-hydroxysuccinimide ester cyanine 5 dye (GE Healthcare, Piscataway, NJ) in the presence of dimethylsulfoxide. The uncoupled dye was removed using a Qiagen PCR cleanup kit according to the the manufacturer’s protocol. The purified cDNA probe was lyophilized and resuspended in 70 μL of Ribohybe (Ventana, Tucson, AZ). Probe was added to the microarray using a lifterslip (Erie Scientific, Portsmouth, NH) and allowed to hybridize in a humidity chamber for 16 hr at 50°C. Each sample was hybridized separately to two arrays with distinct sets of cDNA probes (one set from the BMAP clones and one from the NIA clone set). The combined data from the two probe sets explore the variation in gene expression with 27,265 unique clones. Microarrays were washed with 2 × SSC [300 mM NaCl, 30 mM sodium citrate (pH 7.0)] on a rocker 2 × 10 min at room temperature followed by two washes for 10 min each in 0.2 × SSC at 50°C to remove unbound probe. Microarrays were dried by centrifugation. Tagged image file format (.tif) images were collected using a SA5000 fluorescence scanner (PerkinElmer, Wellesley, MA) and the data collected and analyzed with QuantArray data collection software (PerkinElmer). Signal extraction protocols exported the mean pixel intensity of the upper 65% of signal pixels and the mean pixel intensity of the lower 65% of background pixels.

### Data analysis

We adjusted mean signal intensity for local background by subtracting the mean background intensity. Data for each array set were exported to Arraystat statistical software (Imaging Research, version 1.0, revision 2.0; GE Healthcare). The Arraystat normalization parameters used were “proportional model with offsets, no outlier exclusion,” which log transforms the data (log_10_) and globally centers the transformed data within conditions by subtracting the array mean for all genes present on all arrays in the condition and adding the condition mean for all arrays in the condition. Condition means were globally centered by subtracting the median of the mean signal intensities for the condition and adding the median of the mean signal intensities across all conditions. Modified analysis of variance (ANOVA) (Arraystat F* tests) and significance of differences between means (*z*-tests) were determined using a pooled error model. Centered expression values and test results were exported to Microsoft Excel (Microsoft Corp., Redmond, WA). We converted normalized means and differences between means from log_10_ to log_2_ for ease of comparison with the literature. Data sets were merged and adjustment for multiple testing was conducted on the *p*-values of the statistical tests in the merged data set using the false discovery rate correction with the level of acceptable false positives set at 0.05 for each statistical test (Benjamini and Hochberg 1995). The full set of MAM- and HN2-targeted genes can be found online in Supplemental Material (http://www.ehponline.org/docs/2006/9073/suppl.pdf).

## Results

### Viability and DNA damage in immature neurons

**Figure 1:**
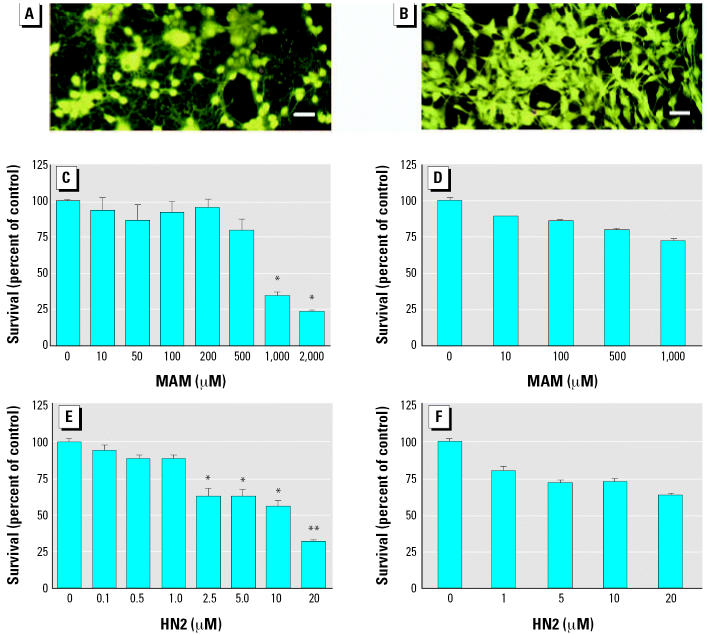
Comparative sensitivity of neurons and astrocytes to MAM or HN2. Representative epifluorescence micrographs of cultures of cerebellar neurons (*A*) and astrocytes (*B*). Bars = 50 μm (*A*) and 100 μm (*B*). Cultures of murine cerebellar granule cells (*C,E*) and astrocytes (*D,F*) were treated with various concentrations of MAM (10–1,000 μM) or HN2 (0.1–20 μM) for 24 hr, incubated with calcein acetoxymethyl ester and the cells examined for fluorescence. Values represent the mean percent survival of controls ± SE (*n* = 6/treatment, 2–3 experiments). Significantly different from control cells (*p < 0.01) or genotoxicant-treated astrocytes (**p < 0.01 by ANOVA).

In the present study, our goal was to determine the relationship between the sensitivity of immature postmitotic neurons to MAM or HN2 and their ability to damage DNA. For these studies, we treated neuronal cell cultures from the cerebellum of neonatal mice with various concentrations of MAM or HN2 for 24 hr and examined them for cell survival (Figure 1A,B). We also similarly treated astrocytes with MAM and HN2 to compare the vulnerability of different CNS cell types to genotoxicants. Survival of cerebellar neurons was significantly reduced with increasing concentrations of MAM (> 100 μM) or HN2 (> 5.0 μM). In contrast, astrocytes derived from the same set of animals were significantly less sensitive (*p* < 0.01) to MAM or HN2. These studies demonstrate that immature neurons are more sensitive to MAM or HN2 than astrocytes, which suggests that this CNS cell type would be preferentially targeted *in vivo* by these genotoxicants.

**Figure 2:**
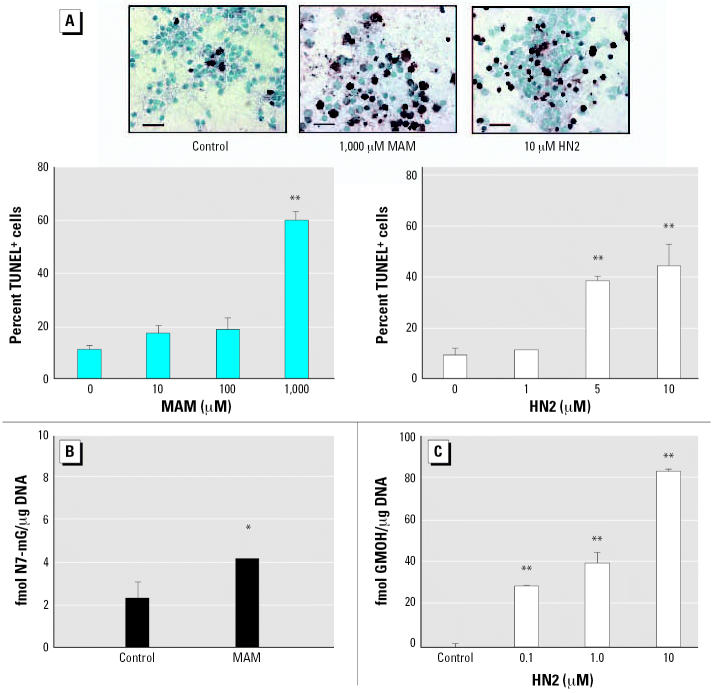
*In situ* DNA damage of cerebellar neurons treated with MAM or HN2. (*A–C*) Representative light micrographs of cerebellar neurons treated for 24 hr with various concentrations of MAM or HN2 and examined for the extent of DNA fragmentation by TUNEL labeling (*A*) or *N*7-alkylguanine DNA lesions induced by 100 μM MAM (*B*) or 0.1–10 μM HN2 (*C*). Note the extensive labeling of neurons treated with 10μM HN2 or 1,000 μM MAM. Bar = 50 μm. Significantly different from control-treated neurons (*p < 0.05 or **p < 0.01 by ANOVA).

Additional studies were conducted to determine if the increased sensitivity of neurons to MAM and HN2 was due to their genotoxic (i.e., DNA damaging) properties. DNA damage was assessed by measuring the level of N7-mG or GMOH, the two major DNA lesions formed by MAM and HN2 (Nagata and Matsumoto 1969; Osborne et al. 1995), or strand breaks (TUNEL labeling). There was a good correlation between the increased sensitivity of neurons to these genotoxicants and TUNEL labeling (Figure 2A) or the level of N7-mG and GMOH DNA lesions (Figure 2B,C). These studies demonstrate that the major DNA lesions formed by MAM or HN2 accumulate in immature neurons and that these cells are particularly inefficient at repairing these types of DNA lesions. Thus, N7-mG and GMOH are likely responsible for the neurotoxic effects of these genotoxicants observed in Figure 1. These findings are also consistent with previous *in vitro* and *in vivo* studies, demonstrating that the increased sensitivity of rat cerebellar neurons or differentiated human SY5Y neuroblastoma cell cultures to HN2 correlated with GMOH levels (Kisby et al. 2000) and N7-mG levels were elevated in the dystrophic cerebellum of neonatal or fetal mice injected with MAM (Kisby et al. 1999, 2005) or other alkylating agents (Buecheler and Kleihues 1977; Kleihues and Bucheler 1977).

### Genotoxicant-induced gene expression changes

**Figure 3:**
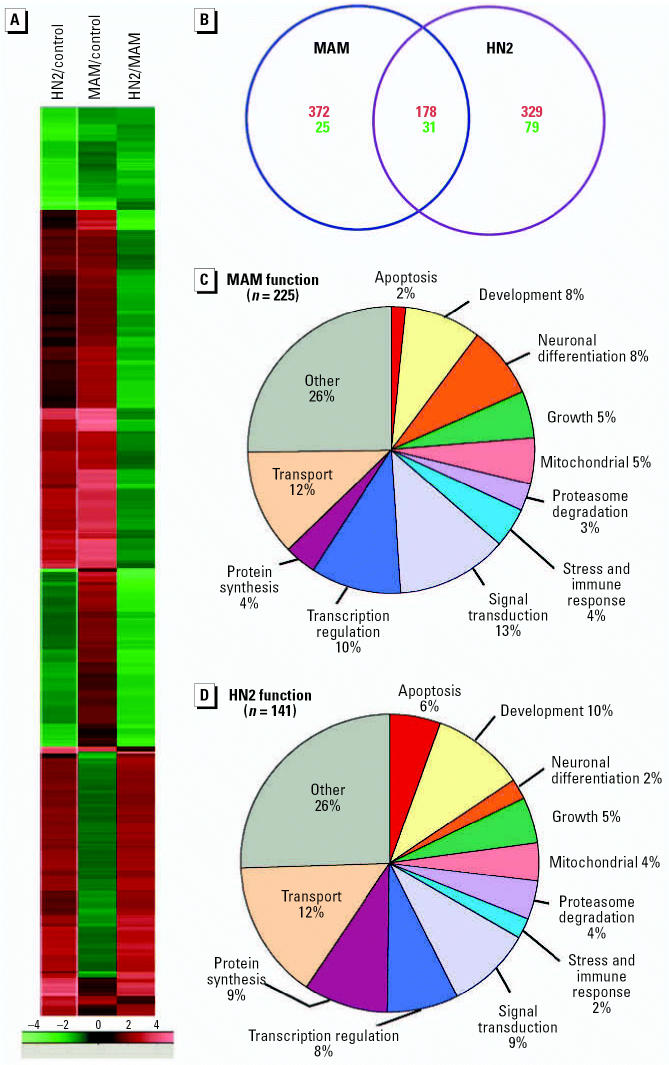
Effect of MAM and HN2 on global gene expression in cultured cerebellar neurons. Mouse cerebellar granule cell cultures were treated with MAM (100 μM) or HN2 (1.0 μM) for 24 hr. (*A*) Gene expression changes were induced by MAM or HN2. All genes with log_2_ MAM/control or HN2/control gene expression ratios > 1 or < –1 were normalized by the absolute valued of the maximum fold change for the gene and grouped by hierarchical clustering using Euclidean distances. (*n* = 606 genes for MAM and 617 genes for HN2). (*B*) Venn diagram depicting the overlap between MAM- and HN2-responsive genes. Up-regulated (*red*): numbers represent all genes with significant differences between MAM or HN2 and control-treated neurons and log_2_ (MAM or HN2/control) > 1. Down-regulated (*green*): significant differences between MAM or HN2 and control-treated neurons and log_2_ (MAM or HN2/control) < –1. (*C*) Functional classes of the genes influenced by MAM. (*D*) Functional classes of the genes influenced by HN2. Named genes with functional annotations in the Unigene database (http://www.ncbi.nlm.nih.gov/UniGene) were categorized by broad functional class.

Collectively, the studies described above and the previous work with these genotoxicants (Dacre and Goldman 1996; Matsumoto et al. 1972; Somani and Babu 1989) indicate that neuronal DNA is a sensitive intracellular target. Failure to repair these DNA lesions would be expected to interfere with transcription and translation (Scicchitano and Mellon 1997; Scicchitano et al. 2004), resulting in perturbed cell function and eventual death via an apoptotic or necrotic mechanism (Dabrowska et al. 1996; Hur et al. 1998; Meier and Millard 1998; Sun et al. 1999). To identify the specific molecular networks targeted by MAM or HN2, we examined genotoxicant-treated neurons for genomewide expression using high-density mouse cDNA microarrays (Figure 3). Our objective here was to determine if these genotoxicants induce a distinct pattern of gene expression at concentrations that are sublethal (Figure 1) and that induce DNA damage (Figure 2B,C). Using these criteria, we treated cerebellar neuronal cultures with 100 μM MAM or 1.0 μM HN2 for 24 hr and examined total RNA for gene expression changes using high-density microarrays. We then compared the gene expression profiles of MAM- and HN2-treated neurons to characterize the response of immature neurons to the two different genotoxicants.

We first used hierarchical clustering (Euclidean distance measure and centroid linkage) to group genes with similar expression levels. Several of these clusters are also specifically enriched with genes of known function. As shown in the heatmap (Figure 3A), we observed distinct clusters for MAM and HN2. The number of genes uniquely regulated by each genotoxicant and their overlap is shown in Figure 3B. The global expression patterns were analyzed further by functional classes of molecules such as DNA repair, cell signaling, proteasome degradation, apoptosis to find correlations among genes and gene-regulatory networks (Figure 3C,D). The global gene expression changes we observed after MAM (606 genes, 2.19%) and HN2 (617 genes, 2.23%) treatment were comparable. Of these global changes, 397 unique genes (64%) were altered by MAM, whereas a similar amount of unique genes (408 genes, 66%) were altered by HN2. Although comparable numbers of unique genes were up-regulated by either MAM or HN2, approximately 3 times as many were down-regulated by HN2 as by MAM (Figure 3B). Among the down-regulated genes, those involved in apoptosis (9.5%) and protein synthesis (4.8%) were targeted by HN2 (*n* = 21), whereas MAM (*n* = 10) primarily targeted those involved in signal transduction (30%), cell adhesion (20%), and growth and cell cycle (10%). These studies indicate that MAM and HN2 target distinct classes of genes in neurons even though both agents alkylate DNA (i.e., the *N*7 site on guanine) and induce a similar global effect on neuronal gene expression. The selective targeting of these functional classes of genes by HN2 and MAM may be related to the different types of DNA lesions generated by these two gentotoxicants; notably, HN2 induces lethal cross-links between opposing N7-alkylguanines (i.e., GMOH) (Osborne et al. 1995; Povirk and Shuker 1994; Tokuda and Bodell 1987), whereas MAM induces methylated DNA lesions (e.g., N7-mG and *O*^6^-mG) (Esclaire et al. 1999; Matsumoto and Higa 1966; Nagata and Matsumoto 1969). The insensitivity of cerebellar neurons to similar concentrations of 2-chloroethylamine (CEA; data not shown), a monofunctional analogue of HN2 that does not induce cross-links (Tokuda and Bodell 1987; Wijen et al. 2000) and the elevated levels of N7-mG DNA lesions in MAM-treated cortical neurons with disturbed tau gene expression (Esclaire et al. 1999) are consistent with this hypothesis.

### Functional classes targeted by MAM and HN2

Even though the majority of genes influenced by sublethal concentrations of MAM or HN2 were of unknown function (63 and 77%, respectively), analysis of the known genes perturbed by MAM (225 genes) or HN2 (141 genes) revealed prominent changes in several different categories (Figure 3C,D), indicating that the molecular networks targeted by these two genotoxicants are quite distinct. As shown in Figure 3C, MAM had a greater influence on genes involved in neuronal differentiation, the stress and immune response, signal transduction, and transcriptional regulation. In contrast, HN2 primarily targeted genes involved in apoptosis and protein synthesis. As expected, MAM had a predominant effect on neuronal differentiation, which was demonstrated by the targeting of a large number of genes that control the growth and maturation of neurons (Table 1). Genes that maintain the structural integrity of neurons (*Prfn2*, *Sdfr1, Catna1, Stmb2*), cellular transport (*Slc6a6*, *Kif1A*), protein degradation (*Usp5*, *Ufd1l*, *Usp2l*, *Psmd12*), or synaptic function (*Vamp4*, *Cplx2*) were specifically targeted by MAM. The increased expression of genes that activate the depolymerization of actin (*Prfn2*) and micro-tubules (*Stmb2*) (Grenningloh et al. 2004; Yarmola and Bubb 2006) is consistent with the ability of MAM to disrupt the outgrowth of axons (Hoffman et al. 1996) and to alter the inward and vertical migration of granule cells through the developing molecular and Purkinje cell layers of the neonatal cerebellum (Ferguson et al. 1996; Kisby et al. 2004). The strong up-regulation of the serine–threonine kinase *Ulk1* and the zeta isoform of protein kinase c (*Prkcz*), which are important regulators of neurite sprouting (Naik et al. 2000; Tomoda et al. 2004), is additional evidence of how this genotoxicant may impede the migration of immature neurons (Hatten 2002).

Although a majority of the genes targeted by MAM were involved in neuronal differentiation, the strongest response was observed for chromatin remodeling (*H3f3a*) (Frank et al. 2003) and energy metabolism (e.g., complex I, glycolytic enzymes) genes. The pronounced targeting of *H3f3a* suggests that MAM may influence transcription by disturbing the nucleosome structure through a chromatin remodeling mechanism (McKittrick et al. 2004). Therefore, the protein encoded by this histone gene may function to maintain chromatin integrity in immature neurons or might be involved with transcription or DNA repair. A corresponding increase in the expression of *Ezh2*, a gene that controls the expression of genes through methylation of H3 (Kirmizis et al. 2004), is consistent with this notion. Unexpectedly, MAM also produced a pronounced effect on the expression of two catalytic subunits (i.e., *Ndufc1*, *Ndufs5*) of complex I (Kirby et al. 2004; Loeffen et al. 1998) and several glycolytic enzymes (*Idh*, *Pk3*), indicating that this genotoxicant also disturbs energy metabolism. The influence of MAM on energy metabolism may explain how this genotoxicant induced lipid peroxidation in the colon and liver of rats (Deschner and Zedeck 1986) and why this effect was counteracted by pretreatment with the antioxidant quercetin (Deschner et al. 1991, 1993).

Even though MAM and HN2 both alkylated neuronal DNA, the genes specifically targeted by HN2 were quite distinct from those targeted by MAM. The most striking difference is that HN2 primarily targeted genes that regulate protein turnover and apoptosis (Figure 3D). Genes that influence the synthesis (*Metap2*, *Mobp*), modification (*Galnt9*), or degradation (*Psme3*) of neuronal proteins were down-regulated by HN2 (Table 2). The increased expression of apoptosis-inducing factor (*Pdcd8*), a flavoprotein that translocates from the mitochondrial intermembrane space to the nucleus to induce caspase-independent DNA fragmentation of cerebellar neurons (Slagsvold et al. 2003) and the targeting of several mitochondrial genes (*Cox7a2*) suggests that HN2-induced neuronal death results from disturbances in mitochondrial function. A concomitant increase in the proteasomal 19s lid component *Psmd7* (or RPN8), which has dual roles in both proteolysis and mitochondrial integrity (Rinaldi et al. 2004), is consistent with this mechanism. However, HN2 had the greatest influence on adenine deaminase (*Ampd3*), an enzyme that maintains steady-state levels of ATP in CNS neurons (Knecht et al. 2001). Because increased AMPD activity is associated with oxidative stress and disturbed calcium homeostasis (Ronquist et al. 2001), HN2 may also induce cell death by disturbing neuronal ATP pools. The concomitant influence of HN2 on Ca^2+^-dependent enzymes (*Calm1, Calm2*) may have contributed to the increased expression of AMPD (Mahnke and Sabina 2005).

Although MAM and HN2 targeted distinct neuronal genes, there were a number of genes that were common targets for both genotoxicants (Table 3). As shown in Table 3, a majority of the genes targeted by both MAM and HN2 were down-regulated. The functional classes of genes specifically targeted by both genotoxicants were also quite distinct from those targeted by each genotoxicant. The strongest response was observed for genes involved in transport (5.7%), development (2.9%), and transcription (2.9%). The targeting of these genes by both genotoxicants may be a signature of a generalized response of neurons to DNA-damaging agents.

### Transcriptional regulatory network analysis

**Figure 4:**
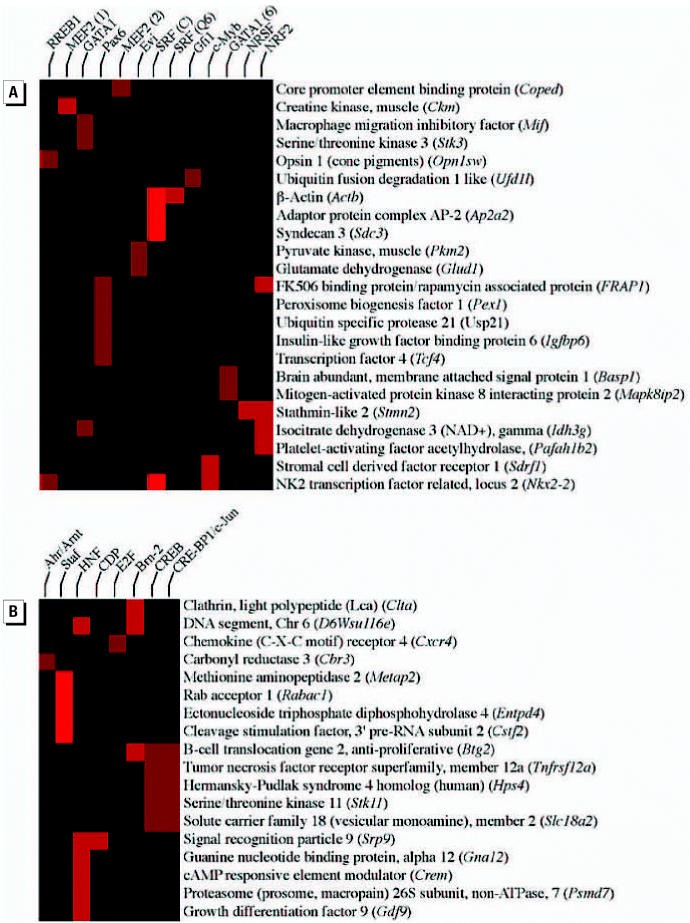
Analysis of the promoter regions of gene sets from MAM and HN2 treated cerebellar neurons for enriched transcriptional regulatory elements (TRE). The 5’-flanking regions (2 kb) of genes that were differentially regulated (factor > 2.0) in cerebellar neurons by MAM (*A*) or HN2 (*B*) were analyzed by PAINT v3.0 to identify overrepresented transcription regulatory elements (TREs). The genes (rows) and motifs (columns) were individually clustered and a subset of those that were found in > 5% of all promoters were used to generate an interaction matrix. Differences in color intensity (i.e., red) indicate the relative frequency of each TRE among the gene sets.

We further analyzed microarray data using the promoter analysis tool PAINT (promoter analysis and interaction network tool) (Vadigepalli et al. 2003) to identify the biologically relevant transcription factor binding sites within the regulatory regions of the genes targeted by HN2 and MAM. Using the unique genes differentially regulated by at least a factor of two after MAM (*n* = 115) or HN2 treatment (*n* = 136), we examined the 5′-flanking regions of these targeted genes (2000 bp upstream of the transcription start site) for enrichment of commonly expressed transcriptional regulatory elements (TRE). The total number of TREs among the unique genes targeted by MAM (*n* = 78) was greater than those targeted by HN2 (*n* = 60). Only TREs that were significantly enriched (*p* < 0.01) in either MAM- or HN2-targeted genes (Figure 4A and 4B, respectively) and occurring in at least 5% of the promoters are shown. Note that no overlap occurred between the TREs enriched in the promoter regions of genes targeted by MAM and HN2 (compare Figure 4A,B). Several MAM-targeted genes were highly enriched for *SRF*, *Nrf2, and Pax6*, whereas *Staf*, *HNF1* and *Cre-BP1* were primarily enriched in HN2-targeted genes. *SRF* is required for neuronal activity–induced gene expression and synaptic plasticity (Ramanan et al. 2005), *Nrf2* is a key regulator of oxidative stress and chemical carcinogen inducible genes (Motohashi and Yamamoto 2004) and *Pax6* controls the polarization and migration of CNS neurons (Yamasaki et al. 2001). Several genes involved in neuronal differentiation and migration (e.g., *Pafah1b2, Stmb2, Actb, Sdrf1, Pex1*) were highly enriched with these TREs, thereby suggesting that these regulatory regions may be important targets by which MAM disrupts cerebellar development. In contrast, *Staf*, *HNF1*, and *Cre-BP1* (or *ATF2*) were especially enriched in HN2-targeted genes involved in protein turnover (e.g., *Cstf2*), the cellular response to DNA damage (Ishiguchi et al. 2004), or cell death mechanisms (Pearson et al. 2005). The enrichment of distinct TREs within MAM- or HN2-targeted genes is additional evidence that these two genotoxicants exert their influence on gene expression in immature neurons by different mechanisms.

## Discussion

Increasing evidence indicates that biomarkers of genetic damage (including DNA lesions) occur in children and newborns exposed to environmental pollutants (Neri et al. 2006). A consistent finding among these studies is the frequent association between the level of DNA lesions and impaired growth during the pre-natal or postnatal period. The increased level of genetic damage reported in these children could also have important adverse health effects on the brain, especially during early development. Consistent with this hypothesis, we have recently shown that DNA damage (i.e., N7-mG) and the perturbation of developmentally regulated genes occurs well before the neurodevelopmental changes induced by the genotoxicant MAM (Kisby et al. 2005). These studies suggest that DNA damage may be responsible for the neurodevelopmental changes induced by the genotoxicant MAM. Thus, our focus in the present studies was to investigate the putative link between genotoxicant-induced DNA damage and neuronal function by identifying the genes in immature neurons specifically targeted by different genotoxicants (i.e., MAM, HN2).

As shown in previous *in vivo* studies (Kisby et al. 2005), we show here that immature cerebellar neurons (i.e., granule cells) are very sensitive to genotoxicants and that this effect was associated with the accumulation of DNA lesions (i.e., N7-mG, GMOH). Our studies also suggest that the DNA damage in the cerebellum of MAM-treated neonatal mice had accumulated in immature granule cells. The greater sensitivity of granule cells compared with astrocytes to either genotoxicant is evidence that neurons are especially vulnerable to genotoxicants and are inefficient at repairing DNA damage. This appears to be a characteristic response of cerebellar neurons to genotoxicants because granule cells are also very sensitive to chemotherapeutic agents that alkylate DNA (e.g., chloronitrosourea) or induce cross-links (e.g., cisplatin) (Fujimori et al. 1992; Jones and Gardner 1976; Wick et al. 2004), whereas glial cell (e.g., astrocytes) loss is not commonly found (Cattaneo et al. 1995; Necchi et al. 1997). This differential sensitivity to genotoxicants is also shared by immature neurons and astrocytes in other brain regions because N7-mG DNA lesions persisted in the cerebrum of neonatal rats after a single *in utero* injection of MAM (Kisby et al. 1999) or related alkylating agents (Buecheler and Kleihues 1977; Kleihues and Bucheler 1977), whereas glial changes were unremarkable (Eriksdotter-Nilsson et al. 1986). Thus, these *in vitro* studies complement previous *in vivo* work by demonstrating that the DNA of immature neurons appears to be an important target for genotoxicants. Moreover, the inefficient removal of DNA lesions in granule cells could also explain why the cerebellum is specifically targeted by genotoxicants (Fonnum and Lock 2000; Jirakulsomchok et al. 1982; Mehl et al. 2000; Singh et al. 1983; Smith et al. 1987) and why cerebellar function is disturbed in both neuro-developmental and DNA repair disorders (Fiore et al. 2004; Wallace et al. 2003).

As noted above, DNA lesions appear to persist in immature neurons of genotoxicant-treated animals. This could explain why the developing cerebellum is a prime target in several human neurodevelopmental disorders (Ahsgren et al. 2005; Bauman and Kemper 2005; Guerrini and Filippi 2005; Hatten 2002). Because DNA lesions (e.g., alkyl or bulky) can influence gene transcription either up or down, depending on the sequence context (Scicchitano et al. 2004), it is conceivable that the DNA lesions formed by MAM or HN2 profoundly influenced the expression of developmentally regulated neuronal genes. Like previous microarray studies of the cerebellum (Kisby et al. 2005), we show that MAM targeted a large number of critically important genes that control the maturation and differentiation of neurons. However, little overlap occurred between the genes targeted by HN2 and MAM, indicating that the different types of DNA lesions (methyl vs. cross-links) produced by these genotoxicants could have been an important contributing factor. This notion is consistent with the distinct gene expression profiles produced in murine cells after treatment with various classes of genotoxicants. In one study, methylating agents (e.g., methyl methane sulfonate), cross-linking agents (e.g., mitomycin C), or agents that form bulky DNA lesions (e.g., benzo[*a*]pyrene) were compared and found to induce gene expression profiles quite distinct from each other and other non-genotoxicants (Newton et al. 2004). Hu and colleagues (2004) reached similar conclusions after examining the gene expression profiles of murine lymphoma cells lines treated for 4 hr and 20 hr with similar classes of genotoxicants. Like the present study, they used concentrations of genotoxicants that induced minimal toxicity (10–30%) so as to avoid the activation of cell death pathways. Therefore, our data indicate that the distinct gene expression changes induced by MAM or HN2 may be due to the influence of DNA lesions produced by these genotoxicants on transcription. Recent microarray studies support this hypothesis by showing that the decline in gene expression within the aging human brain is associated with a corresponding increase in DNA lesions (i.e., 8-oxodexoyguanosine) within the promoter region of key genes involved in learning, memory, and neuronal survival (i.e., synaptic plasticity) (Lu et al. 2004).

Studies on human neuronal migration disorders indicate that defects in migration as well as in proliferation, survival, and differentiation may contribute to neurodevelopmental disorders (Ross and Walsh 2001). The molecular and genetic basis of neuronal migration disorders suggests that the key steps depend on proper actin, microtubule cytoskeletal alterations as well as proper transduction of extra-cellular signals by migrating neurons. One key finding of the present studies is that the molecular pathways controlling neuronal migration and maturation were predominantly targeted by MAM but not by the related genotoxicant HN2. More specifically, MAM had a significant influence on several genes that control the development of neuronal processes (i.e., axons, dendrites) that would markedly impair neuronal growth cone motility and its pathfinding ability (Hatten 1999). The preferential targeting of neuronal differentiation by MAM is also consistent with the ability of this genotoxicant to disrupt unique molecular networks during either fetal (Hoffman et al. 1996) or postnatal (Kisby et al. 2005) neuronal development. The unexpected strong influence of MAM on several genes involved with chromatin remodeling or energy metabolism suggests that these cellular processes may play an important role in the ensuing neurodevelopmental deficits. Consequently, early-life exposure to genotoxicants would be expected to have a pronounced influence on neuronal development and thus, induce long-term changes in CNS function.

In summary, the present studies demonstrate that immature neurons are especially vulnerable to genotoxicants and that this vulnerability is associated with the accumulation of specific DNA lesions and distinct alterations in gene expression. The preferential targeting of genes involved in such diverse functions such as differentiation, stress and immune response, cell signaling, transcriptional regulation by MAM and apoptosis and protein synthesis by HN2 suggests that genotoxicants target distinct neuronal networks and they are likely to induce completely different effects on the developing brain. This is supported by the increased vulnerability of mature neurons to HN2 (Sullivan et al. 1982) but not to MAM (Sullivan-Jones et al. 1994). The preferential targeting of apoptotic networks by HN2 suggests that cross-links (formed between two opposing GMOH DNA lesions) are more likely to activate cell death mechanisms. Consequently, the targeting of specific molecular networks by different gentoxicants may explain the differential response of the developing CNS to different genotoxicants.

## Figures and Tables

**Table 1:** Selected MAM-responsive genes in cerebellar neurons.

GenBank accession no.	Gene name	Gene symbol	Fold change (MAM/control)^a^	Summary function
Highest response to MAM				
AI846799	H3 histone, family 3A	*H3f3a*	3.77	Replacement histone
AI841944	protein kinase C, zeta	*Prkcz*	3.74	Neurite extension
AI850194	Unc-51 like kinase 1	*Ulk1*	3.47	Granule cell axon extension
AI847913	profilin 2	*Pfn2*	3.10	Actin polymerization
Neuronal function				
AI836607	vesicle-associated membrane protein 4	*Vamp4*	2.40	Vesicular trafficing
AI847695	kinesin heavy chain member 1A	*Kif1a*	2.35	Molecular motor
BG085187	neurochondrin	*Ncdn*	2.32	Dendritic outgrowth
AI854735	complexin 2	*Cplx2*	2.08	Synaptic vesicular release
Development				
A1839566	stromal derived factor receptor	*Sdfr1*	2.80	Axonal elongation
AI838741	platelet-activating factor acetylhydrolase, isoform 1b, alpha2 subunit	*Pafah1b2*	2.79	Neuronal migration
AI838754	insulin-like growth factor binding protein 6	*Igfbp6*	2.60	Cerebellar folia
AI842688	stathmin-like 2	*Stmb2*	2.45	Microtuble stability
AI839303	zinc finger protein of the cerebellum 4	*Zic4*	2.25	Neurogenesis
Apoptosis control				
BG077775	tumor necrosis factor receptor superfamily, member 23	*Tnfrsf23*	3.13	Apoptosis control
AI834850	amino-terminal enhancer of split	*Aes*	2.71	NF-kappaB co-repressor
Ubiquitin-proteasome pathway				
AI838669	proteasome (prosome, macropain) 26S subunit, non-ATPase, 12	*Psmd12*	2.75	19S lid component (RPN5)
AI847905	ubiquitin specific protease 5 (isopeptidase T)	*Usp5*	2.60	Deubiqutinating enzyme
AI850551	ubiquitin fusion degradation 1 like	*Ufd1l*	2.43	Polyubiquitin binding
AI843395	ubiquitin specific protease 21	*Usp21*	2.07	Deubiquitinating enzyme
Growth and cell cycle control				
AI841459	diazepam binding inhibitor	*Dbi*	2.53	Lipid metabolism
AI836597	microtubule-associated protein, RP/EB family	*Mapre2*	2.06	Mitotic microtubules
AI323871	cyclin D3	*Ccnd3*	2.06	Neurite outgrowth
AI846429	U7 snRNP-specific Sm-like protein	*Lsm10*	2.02	Histone mRNA processing
Miscellaneous genes of interest				
AI849325	isocitrate dehydrogenase 3 (NAD+), gamma	*Idh3g*	2.97	Mitochondrial respiration
AI840067	NADH dehydrogenase (ubiquinone) 1, subcomplex unknown, 1	*Ndufc1*	2.67	Mitochondrial respiration
AI836137	pyruvate kinase 3	*Pk3*	2.57	Glycolysis
AI838954	catenin alpha 1	*Catna1*	2.35	Axonal reorganization
AI853920	NADH dehydrogenase (ubiquinone) Fe-S protein 5	*Ndufs5*	2.33	Mitochondrial respiration
AI839652	t-complex protein 1, related sequence 1	*Tcp1-rs1*	2.33	Chaperonin protein
AI839531	solute carrier family 25, member 12	*Slc25a12*	2.01	Mitochondrial Asp/Glu transporter
AI323840	enhancer of zeste homolog 2	*Ezh2*	2.01	Histone lysine methyltransfease

NCBI GenBank database (http://www.ncbi.nlm.nih.gov/) was used to obtain gene name, gene symbol, and summary function.

a The fold changes between MAM- and control-treated neurons were statistically significant at fals discovery rate of 0.05 after adjustment for multiple comparisons.

**Table 2:** Selected HN2-responsive genes in cerebellar neurons.

GenBank accession no.	Gene name	Gene symbol	Fold change (HN2/control)^a^	Summary function
Highest response to HN2				
BG080773	AMP deaminase 3	*Ampd3*	4.10	Purine metabolism
BG066562	proteasome (prosome, macropain) 26S subunit, non-ATPase, 7	*Psmd7*	3.86	Protein degradation
C87546	serine/threonine kinase 11	*Stk11*	3.83	Cell cycle and polarity
BG086264	polymerase (RNA) II, DNA directed	*Polr2*	–3.27	RNA synthesis
Neuronal function				
AI850277	neuromedin	*Nmu*	2.03	Locomotor and stress response
A1848307	staufen homolog 2	*Stauf2*	–2.14	RNA transport
AI847890	proteolipid protein	*Plp*	–2.16	Myelination
Development				
BG088163	split hand/foot deleted gene 1	*Shfdg1*	3.08	DNA repair
AU021923	jagged 1	*Jag1*	2.61	Oligodendrocyte development
BG063365	chemokine (C-X-C motif) receptor 4	*Cxcr4*	2.39	Neural progenitors
AI847007	NCK-associated protein 1	*Nckap1*	–2.02	Cell motility
AI843136	N-myc downstream regulated 2	*Ndr2*	–2.23	Neural differentiation
Apoptosis control				
C85471	programmed cell death 8	*Pdcd8*	2.69	Apoptosis control
BG086831	programmed cell death 4	*Pdcd4*	2.13	Apoptosis control
AI853558	tumor necrosis factor receptor superfamily, member 12a	*Tnfrsf12a*	–2.11	Nuclear factor-kappaB activation
Ubiquitin-proteasome pathway				
BG085363	proteasome (prosome, macropain) 26S subunit, non-ATPase, 11	*Psmd11*	2.71	Proteasome (19S Lid)
AI843127	huntingtin interacting protein 2	*Hip2*	2.11	Ubiqutiin-conjugating enzyme
AU020960	proteaseome (prosome, macropain) 28 subunit, 3	*Psme3*	–2.41	Proteasome (20S alpha subunit)
Growth and cell cycle control				
C86021	growth differentiation factor 9	*Gdf9*	3.61	Cell growth
AI853288	ras homolog gene family, member U	*Arhu*	2.33	Signal transduction
BG072244	calmodulin 1	*Calm1*	2.13	Cell cycle
AI843756	calmodulin 2	*Calm2*	–2.16	Cell cycle
Miscellaneous genes of interest				
AI851097	H1 histone family, member 2	*H1f2*	–2.36	Chromatin compaction
AI849019	myelin-associated oligodendrocytic basic protein	*Mobp*	–2.71	Stuctural components of myelin

GenBank database (http://www.ncbi.nlm.nih.gov/) was used to obtain gene name, gene symbol, and summary function.

a The fold changes between HN2- and control-treated neurons were statistically significant at false discovery rate of 0.05 after adjustment for multiple comparisons.

**Table 3:** Selected MAM- and HN2-responsive genes in mouse cerebellar neurons.

			Fold change^a^		
GenBank accession no.	Gene name	Gene symbol	MAM/control	HN2/control	Summary function
Highest response to MAM and HN2					
AI836491	heat shock 10 kDa protein 1 (chaperonin 10)	*Hspe1*	4.03	2.99	Mitochondrial chaperone
AI843553	heat shock 70kD protein 5 (glucose-regulated protein, 78kD)	*Hspa5*	3.11	2.21	ER stress response
BG088092	solute carrier family 14, member 1	*Slc14a1*	–2.70	–2.10	Urea transport
AI847514	solute carrier family 1, member 3	*Slc1a3*	–2.86	–2.17	Glial glutamate transport
Development					
AI841643	platelet derived growth factor, B polypeptide	*Pdgfb*	3.01	2.54	Neuronal migration
AI846342	membrane-type frizzled-related protein	*Mfrp*	2.58	2.39	Tissue polarity
AI838959	actin, alpha 2, smooth muscle, aorta	*Acta2*	2.56	2.14	Cytoskeleton organization
Signal transduction/transport					
AI835905	ferritin heavy chain	*Fth*	2.63	2.19	Iron storage factor
AI836589	ATP synthase, H^+^ transporting mitochondrial F1 complex, beta subunit	*Atp5b*	2.54	2.46	Mitochondrial transport
AI843291	synbindin	*Sbdn*	2.41	2.16	Vesicular transport
AI842821	phospholipase C-like 2	*Plcl2*	2.15	2.12	Vesicular transport
Transcription					
AI837833	zinc finger protein 95	*Zfp95*	2.87	2.86	Transcription regulator
AI845485	four and a half LIM domains 4	*Fhl4*	2.65	2.07	Transcriptional co-activator
AI835325	kelch-like ECH-associated protein 1	*Keap1*	2.40	2.02	Transcription regulator
AI842684	interferon regulatory factor 3	*Irf3*	–2.01	–2.10	Transcription regulator
Miscellaeneous genes of interest					
AI841630	ATP citrate lyase	*Acly*	2.51	2.19	Acetyl-CoA synthesis
AI839804	CDC-like kinase 2	*Clk2*	2.02	2.08	Synaptic reorganization
BG081218	DNA cross-link repair 1A, PSO2 homolog (S. cerevisiae)	*Dclre1a*	–2.13	–2.27	DNA cross-link repair
BG069818	ubiquitin specific protease 3	*Usp3*	–2.43	–2.55	Deubiquitinating enzyme
BG075881	tyrosine 3-monooxygenase/tryptophan 5-monooxygenase activation protein, zeta polypeptide	*Ywhaz*	–2.48	–2.44	Cell adhesion

GenBank database (http://www.ncbi.nlm.nih.gov/) was used to obtain gene name, gene symbol, and summary function.

a The fold changes between MAM- and control-treated and HN2- and control-treated neurons were statistically significant at false discovery rate of 0.05 after adjustment for multiple comparisons.
