# Supplementary material for: Genotoxicants Target Distinct Molecular Networks in Neonatal Neurons
Source: Environ Health Perspect. 2006 Sep 7;114(11):1703–12. doi: 10.1289/ehp.9073 (PMC1665395; doi:10.1289/ehp.9073)
Supplement: Supplemental Figures and Tables [file ehp0114-001703s1.pdf]

Table S1 Changes in Gene Expression Associated with MAM treatment in Cultured Mouse Neurons*

Accession ID	Gene Name	Gene Symbol	MAM / Control				Summary Function
			Ratio (Log2)	Fold change	[illegible] (z test using pooled error estimate)	statistically significant (adjusted for multiple comparisons)	
AI528522	protein kinase C, theta	*Prkcq*	1.02	2.03	2.85E-04	YES	protein modification
BG075444	ESTs		-1.04	-2.06	2.07E-04	YES	
AI327089	homer, neuronal immediate early gene, 3	*Homer3-pending*	1.36	2.57	1.26E-06	YES	signal transducer
AI854307	RIKEN cDNA 2700071E21 gene	*2700071E21Rik*	1.28	2.43	4.84E-06	YES	
AI854626	lymphocyte antigen 6 complex, locus A	*Ly6a*	1.25	2.37	9.14E-06	YES	other
AW555822			1.08	2.12	1.12E-04	YES	
AW555902			1.37	2.58	1.13E-06	YES	
AI850899	NK2 transcription factor related, locus 2 (Drosophila)	*Nkx2-2*	1.55	2.93	3.33E-08	YES	development
AI853881	Mus musculus, similar to programmed cell death 6 interacting protein, clone MGC:40682 IMAGE:3498026, mRNA, complete cds		1.51	2.84	8.06E-08	YES	
AW559004			1.15	2.21	4.36E-05	YES	
AI837067	hypothetical protein (B2 element) - mouse [M.musculus]		1.52	2.86	6.60E-08	YES	
AI837840			1.68	3.19	2.38E-09	YES	
AI840161	acetyl-Coenzyme A acetyltransferase 1	*Acat1*	1.63	3.11	5.70E-09	YES	mitochondria
AI836765	EST		1.19	2.28	2.20E-05	YES	
AI838008	glyoxylate reductase/hydroxypyruvate reductase	*Grhpr*	1.73	3.32	6.75E-10	YES	other
AI839520	guanylate kinase 1	*Guk1*	1.63	3.10	6.04E-09	YES	other
AI835905	ferritin heavy chain	*Fth*	1.40	2.63	6.62E-07	YES	transport
AI836319	EST		2.12	4.36	3.75E-14	YES	
AI836814	EST		1.80	3.48	1.46E-10	YES	
AI838917	EST		1.20	2.30	1.81E-05	YES	
AI838922	EST		1.84	3.59	5.20E-11	YES	
AI839395	RIKEN cDNA 2900055D03 gene	*2900055D03Rik*	1.14	2.20	4.97E-05	YES	mitochondria
AI845896	follistatin-like 3	*Fstl3*	1.67	3.19	2.53E-09	YES	unknown
AI845823	(protein for IMAGE:3610257), clone MGC:37326 IMAGE:4975562, mRNA, complete cds		1.73	3.32	6.87E-10	YES	
AI842725	integrin alpha 6	*Itga6*	1.22	2.33	1.37E-05	YES	cell adhesion
AI841106	cyclin-dependent kinase 5	*Cdk5*	-1.05	-2.07	1.86E-04	YES	cell cycle

AI838959	actin, alpha 2, smooth muscle, aorta	*Acta2*	1.36	2.56	1.38E-06	YES	cytoskeleton organization
AI840342	EST		1.67	3.18	2.63E-09	YES	
AI837442			1.71	3.28	1.04E-09	YES	
AI836538	EST		1.47	2.77	1.63E-07	YES	
AI838727	EST		1.60	3.03	1.17E-08	YES	
AI838731	DNA segment, Chr 17, Wayne State University 92, expressed	*D17Wsu92e*	1.05	2.07	1.79E-04	YES	
AI839746	lysophospholipase 3	*Lypla3*	1.12	2.17	6.56E-05	YES	other
AI839792	ESTs		1.53	2.88	5.47E-08	YES	
AI835809	RIKEN cDNA 2810432L12 gene	*2810432L12Rik*	1.10	2.14	9.01E-05	YES	
AI840067	acetylglucosaminyltransferase 1	*Mgat1*	1.41	2.66	4.71E-07	YES	other
AI838763	expressed sequence AI838763	*AI838763*	1.41	2.65	5.46E-07	YES	
AI838773	expressed sequence AI838773	*AI838773*	1.62	3.07	7.87E-09	YES	
AI839566	stromal cell derived factor receptor 1	*Sdfr1*	1.49	2.80	1.19E-07	YES	unknown
AI841636	RIKEN cDNA 1810021J13 gene	*1810021J13Rik*	1.22	2.33	1.43E-05	YES	unknown
AI841642	retinoic acid receptor, gamma	*Rarg*	1.28	2.42	5.45E-06	YES	signal transducer
AI840852	EST		1.66	3.16	3.19E-09	YES	
AI846298	EST		1.00	2.00	3.64E-04	YES	
AI840864	ESTs		1.01	2.02	3.05E-04	YES	
AI843344	ESTs		1.71	3.27	1.11E-09	YES	
AI843586	[illegible] kDa)	*Sfrs9*	1.78	3.44	2.12E-10	YES	RNA processing
AI843670	RIKEN cDNA A230106A15 gene	*A230106A15Rik*	1.83	3.56	7.03E-11	YES	
AI840542			2.13	4.39	2.89E-14	YES	
AI843240	apoptosis-associated speck-like protein containing a CARD	*Asc-pending*	1.37	2.59	9.91E-07	YES	apoptosis control
AI846342	membrane-type frizzled-related protein	*Mfrp*	1.37	2.58	1.15E-06	YES	development
AI837122	EST		1.43	2.69	3.56E-07	YES	
AI843257	hypothetical protein MGC36831	*MGC36831*	1.60	3.04	1.14E-08	YES	
AI842842	RIKEN cDNA 1110051M15 gene	*1110051M15Rik*	1.28	2.43	5.23E-06	YES	development
AI842511			1.85	3.60	4.45E-11	YES	
AI837833	zinc finger protein 95	*Zfp95*	1.52	2.87	5.76E-08	YES	transcription regulator
AI835706	RAB3D, member RAS oncogene family	*Rab3d*	1.51	2.85	7.06E-08	YES	signal transducer
AI840192	ESTs, Weakly similar to S19560 proline-rich protein MP4 - mouse [M.musculus]		1.01	2.02	3.04E-04	YES	
AI835726	RIKEN cDNA 1810060D16 gene	*1810060D16Rik*	1.20	2.30	1.81E-05	YES	transcription
AI836764	similar to hypothetical protein FLJ21919	*LOC229543*	1.27	2.40	6.49E-06	YES	

AI837886	ATP-binding cassette, sub-family E (OABP), member 1	*Abce1*	1.14	2.20	5.23E-05	YES	transport
AI838001	hypothetical protein MGC25509	*MGC25509*	1.04	2.06	2.09E-04	YES	
AI841895	NR1D1 (V-erbA related protein EAR-1) (Rev-erbA-alpha)	*LOC217166*	-1.00	-2.00	3.63E-04	YES	signal transducer
AI841920	expressed sequence AL022641	*AL022641*	-1.38	-2.60	9.43E-07	YES	transport
AI839531	solute carrier family 25 (mitochondrial carrier, Aralar), member 12	*Slc25a12*	1.00	2.01	3.47E-04	YES	transport
AI837396	EST		1.84	3.57	5.92E-11	YES	
AI839397	RIKEN cDNA 4833424K13 gene	*4833424K13Rik*	1.76	3.39	3.48E-10	YES	
AI840334	EST, Weakly similar to beta-tubulin cofactor C; tubulin-specific chaperone c [Homo sapiens] [H.sapiens]		1.78	3.44	2.13E-10	YES	
AI841385	Noelin precursor (Neuronal olfactomedin-related ER localized protein) (Olfactomedin 1) (Pancortin) [M.musculus]		1.38	2.61	8.46E-07	YES	
AI845897	hypothetical protein MGC7807	*MGC7807*	1.29	2.44	4.54E-06	YES	
AI846635	ESTs		1.16	2.24	3.46E-05	YES	
AI842684	interferon regulatory factor 3	*Irf3*	-1.00	-2.01	3.43E-04	YES	transcription regulator
AI845582	sparc/osteonectin, cwcv and kazal-like domains proteoglycan 1	*Spock1*	1.67	3.18	2.76E-09	YES	unknown
AI845829	asialoglycoprotein receptor 1	*Asgr1*	1.54	2.90	4.52E-08	YES	signal transducer
AI837820	hypothetical protein MGC38208	*MGC38208*	1.01	2.02	3.02E-04	YES	other
AI845839	RIKEN cDNA 4930447D24 gene	*4930447D24Rik*	1.18	2.26	2.68E-05	YES	unknown
AI840919	DNA-damage inducible transcript 3	*Ddit3*	1.45	2.74	2.24E-07	YES	apoptosis control
AI845634	RIKEN cDNA 4930586I02 gene	*4930586I02Rik*	1.07	2.10	1.38E-04	YES	
AI845870	EST		1.54	2.90	4.46E-08	YES	
AI840807	beta-spectrin 4	*Spnb4*	1.84	3.59	5.09E-11	YES	scytoskeleton
AI836047	ESTs		1.60	3.03	1.27E-08	YES	
AI837935	EST		1.65	3.15	3.79E-09	YES	
AI839702	dismutase	*Ccs*	1.27	2.40	6.52E-06	YES	chaperone
AI837458	RIKEN cDNA 4933405K21 gene	*4933405K21Rik*	1.02	2.03	2.61E-04	YES	
AI840047	EST		1.58	2.98	1.95E-08	YES	
AI839815	EST		1.39	2.63	6.84E-07	YES	
AI839834	heat shock protein, 30 kDa	*Hsp30-pending*	1.12	2.17	7.02E-05	YES	heat shock response
AI838772	RIKEN cDNA 2810481F14 gene	*2810481F14Rik*	1.02	2.03	2.60E-04	YES	
AI838774	similar to hypothetical protein MGC2494	*LOC214917*	1.09	2.13	1.05E-04	YES	
AI835878	EST		1.39	2.61	7.88E-07	YES	

AI840113	peroxiredoxin 2	*Prdx2*	1.71	3.27	1.08E-09	YES	apoptosis control
AI841630	ATP citrate lyase	*Acly*	1.33	2.51	2.26E-06	YES	other
AI841633	ESTs		1.18	2.27	2.56E-05	YES	
AI841643	polypeptide	*Pdgfb*	1.59	3.01	1.51E-08	YES	cell cycle
AI843335	expressed sequence AU040105	*AU040105*	1.04	2.06	2.03E-04	YES	
AI843538	ESTs		1.52	2.87	6.03E-08	YES	
AI843248	RIKEN cDNA 5730405I09 gene	*5730405I09Rik*	1.83	3.55	7.13E-11	YES	cell cycle
AI842510	TRB-2	*TRB-2*	1.53	2.89	5.11E-08	YES	
AI843286			1.69	3.23	1.71E-09	YES	
AI843291	synbindin	*Sbdn*	1.27	2.41	5.93E-06	YES	transport
AI843073	RIKEN cDNA 5430411C10 gene	*5430411C10Rik*	1.30	2.46	3.86E-06	YES	
AI845395	transmembrane protein 2	*Tmem2*	1.25	2.38	8.69E-06	YES	
AI842552	RIKEN cDNA C030036K04 gene	*C030036K04Rik*	1.12	2.18	6.30E-05	YES	
AI323840	enhancer of zeste homolog 2 (Drosophila)	*Ezh2*	1.00	2.01	3.48E-04	YES	transcription regulator
BG076138	ESTs		1.87	3.67	2.41E-11	YES	
AI413648	ESTs		-1.25	-2.39	7.84E-06	YES	
BG088381	RIKEN cDNA 2010100O12 gene	*2010100O12Rik*	1.17	2.25	3.03E-05	YES	unknown
AI327246	ESTs, Weakly similar to S20900 titin - mouse (fragment) [M.musculus]		-1.15	-2.21	4.36E-05	YES	
BG067308	5730493B19 [Mus musculus] [M.musculus]		-1.07	-2.10	1.34E-04	YES	
AI854139	ESTs, Moderately similar to T00388 hypothetical protein KIAA0616 - human (fragment) [H.sapiens]		1.25	2.38	8.14E-06	YES	
AI854015	syndecan 3	*Sdc3*	1.07	2.10	1.38E-04	YES	neuronal function
AI853588	ubiquitin-like 4	*Ubl4*	-1.10	-2.14	9.30E-05	YES	transport
AI853599	B lymphocyte gene 1	*Bce1-pending*	-1.03	-2.04	2.58E-04	YES	transcription regulator
AI853621	DNA segment, Chr 4, Wayne State University 114, expressed	*D4Wsu114e*	-1.19	-2.28	2.21E-05	YES	
AI323833	GPI-anchored membrane protein 1	*Gpiap1*	1.46	2.75	2.01E-07	YES	
AI851062	RIKEN cDNA 1300009F09 gene	*1300009F09Rik*	-1.17	-2.25	2.99E-05	YES	transport
AI323847	macrophage migration inhibitory factor	*Mif*	1.23	2.35	1.17E-05	YES	signal transducer
BG076146	secretory carrier membrane protein 5	*Scamp5*	1.05	2.07	1.87E-04	YES	other
AI414844	RIKEN cDNA 2310035L15 gene	*2310035L15Rik*	1.31	2.48	3.18E-06	YES	
BG075859	Nedd4 WW binding protein 4	*N4wbp4-pending*	-1.21	-2.31	1.63E-05	YES	unknown

BG075875	ESTs		-1.61	-3.05	9.92E-09	YES	
BG075881	tyrosine 3-monooxygenase/tryptophan 5-monooxygenase activation protein, zeta polypeptide	*Ywhaz*	-1.31	-2.48	3.07E-06	YES	cell adhesion
AI854735	complexin 2	*Cplx2*	1.05	2.08	1.73E-04	YES	neuronal function
AI851591	brain chitinase like protein 2	*Bclp2*	1.03	2.04	2.53E-04	YES	
AI894122	ESTs, Highly similar to I49561 C33/R2/IA4 - mouse [M.musculus]		1.14	2.21	4.61E-05	YES	unknown
AI854037	membrane protein, palmitoylated 3 (MAGUK p55 subfamily member 3)	*Mpp3*	-1.22	-2.33	1.42E-05	YES	signal transducer
AI853442	RIKEN cDNA 4930404J24 gene	*4930404J24Rik*	1.33	2.52	2.08E-06	YES	
AI854302	clone IMAGE:3491602, mRNA, partial cds		1.58	3.00	1.67E-08	YES	
AI324268	creatine kinase, muscle	*Ckmm*	1.05	2.06	1.94E-04	YES	other
AI853920	NADH dehydrogenase (ubiquinone) Fe-S protein 5	*Ndufs5*	1.22	2.33	1.40E-05	YES	
BG075742	RIKEN cDNA 1110059E24 gene	*1110059E24Rik*	-1.07	-2.09	1.47E-04	YES	
AI854277	serine/threonine kinase 23	*Stk23*	1.16	2.23	3.68E-05	YES	other
AI854068	RIKEN cDNA 1110018M03 gene	*1110018M03Rik*	1.52	2.87	6.26E-08	YES	unknown
AI854292	transcription factor 4	*Tcf4*	1.31	2.48	2.98E-06	YES	development
AI854294	expressed sequence AA986363	*AA986363*	1.18	2.26	2.65E-05	YES	
AI854072	nucleoporin 210	*Nup210*	1.28	2.43	4.88E-06	YES	other
AW556082	aladin	*LOC223921*	1.02	2.02	2.98E-04	YES	
AW556086			1.15	2.21	4.45E-05	YES	
AI851141	ESTs		1.43	2.70	3.32E-07	YES	
AI414793	RIKEN cDNA 2010003K11 gene	*2010003K11Rik*	-1.10	-2.14	8.82E-05	YES	
BG076082	peroxisome biogenesis factor 1	*Pex1*	-1.03	-2.04	2.49E-04	YES	other
AI835656			1.18	2.27	2.59E-05	YES	
AI839988	hypothetical protein MGC38419	*MGC38419*	1.47	2.77	1.67E-07	YES	transport
AI840245	ESTs, Highly similar to T00370 hypothetical protein KIAA0659 - human (fragment) [H.sapiens]		1.63	3.10	6.23E-09	YES	
AI839148			1.06	2.09	1.56E-04	YES	
AI840263	NADH dehydrogenase (ubiquinone) 1, subcomplex unknown, 1	*Ndufc1*	1.42	2.67	4.61E-07	YES	unknown
AI839178	retinal S-antigen	*Sag*	1.34	2.53	1.87E-06	YES	signal transducer
AI839182	Mus musculus, Similar to cyclic GMP stimulated phosphodiesterase, clone IMAGE:3598413, mRNA, partial cds		1.13	2.19	5.37E-05	YES	
AI838564	expressed sequence AL024016	*AL024016*	1.20	2.30	1.91E-05	YES	
AI838592	expressed sequence AL024016	*AL024016*	1.30	2.47	3.43E-06	YES	
AI838600	EST		1.69	3.23	1.66E-09	YES	

AI840024	apolipoprotein D	*Apod*	1.22	2.34	1.30E-05	YES	transport
AI843449	RIKEN cDNA 4921531G14 gene	*4921531G14Rik*	1.03	2.05	2.35E-04	YES	
AI845559	RIKEN cDNA 2210021K23 gene	*2210021K23Rik*	1.08	2.12	1.11E-04	YES	
AI846765	expressed sequence AW048546	*AW048546*	1.27	2.42	5.66E-06	YES	
AI836642	EST		1.13	2.19	5.79E-05	YES	
AI836953			1.61	3.06	8.87E-09	YES	
AI838656			1.30	2.46	3.53E-06	YES	
AI838691	sorting nexin 4	*Snx4*	1.23	2.35	1.13E-05	YES	transport
AI838366	bromodomain-containing 4	*Brd4*	1.42	2.67	4.43E-07	YES	unknown
AI836470	Sjogren's syndrome/scleroderma autoantigen 1 homolog (human)	*Sssca1*	1.17	2.26	2.87E-05	YES	
AI841825	RIKEN cDNA 6330580J24 gene	*6330580J24Rik*	1.70	3.26	1.25E-09	YES	
AI838890	RIKEN cDNA 2810484M10 gene	*2810484M10Rik*	1.06	2.09	1.52E-04	YES	unknown
AI836488	enoyl Coenzyme A hydratase, short chain, 1, mitochondrial	*Echs1*	1.13	2.19	5.84E-05	YES	mitochondria
AI836491	10)	*Hspe1*	2.01	4.03	7.99E-13	YES	chaperone
AI841766	hypothetical protein DKFZp761A132	*DKFZp761A132*	1.15	2.23	3.91E-05	YES	
AI841777	similar to pyridoxal kinase [Rattus norvegicus]	*BC027745*	1.17	2.24	3.29E-05	YES	
AI841459	diazepam binding inhibitor	*Dbi*	1.34	2.53	1.89E-06	YES	other
AI836010	RIKEN cDNA 1110013B16 gene	*1110013B16Rik*	2.10	4.30	6.75E-14	YES	unknown
AI839303	zinc finger protein of the cerebellum 4	*Zic4*	1.17	2.25	3.07E-05	YES	neurogenesis
AI841463	NK6 transcription factor related, locus 2 (Drosophila)	*Nkx6-2*	2.12	4.34	4.42E-14	YES	transcription regulator
AI846799	H3 histone, family 3A	*H3f3a*	1.91	3.77	9.35E-12	YES	
AI846484	DNA segment, Chr 1, ERATO Doi 396, expressed	*D1Ertd396e*	1.61	3.06	9.09E-09	YES	
AI846429	LSM10	*Lsm10-pending*	1.02	2.02	2.96E-04	YES	RNA processing
AI841315	EST		1.83	3.57	6.27E-11	YES	
AI846467	ESTs		1.08	2.11	1.26E-04	YES	
AI843621	expressed sequence AI836659	*AI836659*	1.76	3.38	3.73E-10	YES	
AI835657	EST		1.55	2.93	3.22E-08	YES	
AI839979	expressed sequence AI839979	*AI839979*	1.01	2.01	3.26E-04	YES	
AI839850	ESTs		1.60	3.04	1.10E-08	YES	
AI835325	kelch-like ECH-associated protein 1	*Keap1*	1.27	2.40	6.49E-06	YES	transcription regulator
AI836605	inhibitor of growth family, member 4	*Ing4*	1.97	3.90	2.54E-12	YES	transcription regulator

AI836607	vesicle-associated membrane protein 4	*Vamp4*	1.26	2.40	6.65E-06	YES	kinesin complex
AI838301	RIKEN cDNA A930040G15 gene	*A930040G15Rik*	1.22	2.33	1.36E-05	YES	
AI839136	EST		1.32	2.49	2.71E-06	YES	
AI839139	septin 9	*sep9*	1.60	3.03	1.19E-08	YES	DNA repair
AI840241			1.56	2.95	2.78E-08	YES	
AI836925	G3P_MOUSE Glyceraldehyde 3-phosphate dehydrogenase (GAPDH) [M.musculus]		1.53	2.89	4.75E-08	YES	
AI836137	pyruvate kinase 3	*Pk3*	1.36	2.57	1.21E-06	YES	other
AI839628			1.77	3.41	2.86E-10	YES	
AI840292	RIKEN cDNA 1300002C13 gene	*1300002C13Rik*	1.49	2.81	1.14E-07	YES	other
AI836141	adenosine A2b receptor	*Adora2b*	1.42	2.68	3.90E-07	YES	signal transducer
AI839633	KH domain containing, RNA binding, signal transduction associated 1	*Khdrbs1*	1.05	2.07	1.78E-04	YES	transcription regulator
AI841944	protein kinase C, zeta	*Prkcz*	1.90	3.74	1.23E-11	YES	signal transducer
AI840487	expressed sequence C80612	*C80612*	1.83	3.55	7.24E-11	YES	other
AI840036	RIKEN cDNA 2610301B20 gene	*2610301B20Rik*	1.53	2.89	4.71E-08	YES	
AI843761	RIKEN cDNA 2610041P16 gene	*2610041P16Rik*	1.01	2.01	3.21E-04	YES	mitochondria
AI837324	cDNA sequence BC021769	*BC021769*	1.44	2.72	2.78E-07	YES	
AI845464	RIKEN cDNA 1700042O10 gene	*1700042O10Rik*	2.14	4.40	2.69E-14	YES	
AI837600	expressed sequence AI850305	*AI850305*	1.54	2.92	3.74E-08	YES	
AI845502	eukaryotic translation initiation factor 3	*Eif3*	2.29	4.89	4.44E-16	YES	protein synthesis
AI843795	6-phosphogluconolactonase	*Pgls*	1.03	2.04	2.57E-04	YES	other
AI843797	hemoglobin alpha, adult chain 1	*Hba-a1*	1.45	2.74	2.17E-07	YES	transport
AI837602	EST		1.65	3.13	4.37E-09	YES	
AI845510	ESTs		2.15	4.45	1.71E-14	YES	
AI837644	RIKEN cDNA 2900002K07 gene	*2900002K07Rik*	2.26	4.78	8.88E-16	YES	unknown
AI846726	EST		1.92	3.78	7.83E-12	YES	
AI842595	ESTs		1.57	2.97	2.18E-08	YES	
AI842597	transcription factor 4	*Tcf4*	1.76	3.39	3.38E-10	YES	development
AI846760	opsin 1 (cone pigments), short-wave-sensitive (color blindness, tritan)	*Opn1sw*	1.24	2.37	9.41E-06	YES	signal transducer
AI846764	biglycan	*Bgn*	1.41	2.65	5.38E-07	YES	other
AI846777			1.06	2.09	1.58E-04	YES	
AI836947	EST		1.69	3.23	1.74E-09	YES	
AI836949	[illegible] dismutase	*Ccs*	1.16	2.23	3.81E-05	YES	chaperone
AI839330	neuron specific gene family member 1	*Nsg1*	1.34	2.54	1.70E-06	YES	neurogenesis

AI836952	BM88 antigen	*Bm88-pending*	1.46	2.75	1.99E-07	YES	unknown
AI836958	zinc finger protein 263	*Zfp263*	1.60	3.02	1.28E-08	YES	transcription regulator
AI836990	pituitary tumor-transforming 1	*Pttg1*	1.46	2.75	2.04E-07	YES	unknown
AI839367	ESTs		1.29	2.44	4.56E-06	YES	unknown
AI838874	protein phosphatase 2a, catalytic subunit, beta isoform	*Ppp2cb*	1.14	2.20	5.25E-05	YES	protein modification
AI836057	RIKEN cDNA 1110012M11 gene	*1110012M11Rik*	1.38	2.60	9.18E-07	YES	
AI841793	dystonin	*Dst*	1.53	2.89	4.72E-08	YES	apoptosis control
AI841235			1.39	2.63	6.81E-07	YES	
AI842648	RIKEN cDNA 5430410K23 gene	*5430410K23Rik*	1.22	2.32	1.49E-05	YES	
AI841241	galactosidase, beta 1	*Glb1*	1.76	3.38	3.98E-10	YES	other
AI843549	DNA segment, Chr 12, ERATO Doi 482, expressed	*D12Ertd482e*	1.08	2.11	1.25E-04	YES	
AI846120	neuropathy target esterase	*Nte*	1.51	2.84	8.08E-08	YES	
AI835542			1.13	2.19	5.71E-05	YES	
AI836097	EST		1.45	2.73	2.36E-07	YES	
AI840172	myelin protein zero	*Mpz*	1.11	2.16	7.78E-05	YES	neuronal function
AI839010			1.43	2.69	3.66E-07	YES	
AI834850	amino-terminal enhancer of split	*Aes*	1.44	2.71	3.08E-07	YES	development
AI835551	RIKEN cDNA 1300017E09 gene	*1300017E09Rik*	1.22	2.33	1.40E-05	YES	
AI837859	RIKEN cDNA 1810036J22 gene	*1810036J22Rik*	2.21	4.62	3.77E-15	YES	
AI836772	EST		1.02	2.03	2.74E-04	YES	
AI839508	1-acylglycerol-3-phosphate O-acyltransferase 3	*Agpat3*	1.21	2.31	1.72E-05	YES	other
AI836782	chondroitin sulfate proteoglycan 5	*Cspg5*	1.11	2.16	7.31E-05	YES	
AI836786	complement component 1, q subcomponent, alpha polypeptide	*C1qa*	1.19	2.27	2.41E-05	YES	immune response
AI841880	molecule	*Esam-pending*	1.53	2.90	4.64E-08	YES	cell adhesion
AI841883			2.06	4.16	2.43E-13	YES	
AI837370	EST, Weakly similar to JW0059 mtprd protein - mouse [M.musculus]		1.82	3.53	9.34E-11	YES	
AI839535	ESTs		1.51	2.85	7.15E-08	YES	
AI838908	secretory carrier membrane protein 5	*Scamp5*	1.48	2.79	1.28E-07	YES	
AI838914	Mus musculus, Similar to RIKEN cDNA 3110039L19 gene, clone IMAGE:4238005, mRNA, partial cds		1.23	2.35	1.16E-05	YES	
AI839548			1.98	3.94	1.90E-12	YES	
AI837434	RIKEN cDNA 4933428G20 gene	*4933428G20Rik*	1.39	2.62	7.41E-07	YES	

AI838946	Mus musculus, clone IMAGE:4527484, mRNA, partial cds		1.61	3.05	9.57E-09	YES	
AI845846	ESTs		1.51	2.86	6.90E-08	YES	
AI845613	RIKEN cDNA 4833415N24 gene	*4833415N24Rik*	1.11	2.15	8.21E-05	YES	
AI845665			1.79	3.45	1.95E-10	YES	
AI840835	EST		1.21	2.31	1.61E-05	YES	
AI843530			1.21	2.32	1.55E-05	YES	
AI836199	protein kinase, cAMP dependent regulatory, type I, alpha	*Prkar1a*	1.80	3.47	1.54E-10	YES	development
AI836856	ESTs, Moderately similar to T08781 hypothetical protein DKFZp586L1220.1 - human (fragment) [H.sapiens]		1.48	2.79	1.36E-07	YES	
AI837467	RIKEN cDNA 1110021E09 gene	*1110021E09Rik*	1.68	3.21	1.96E-09	YES	
AI836532	cysteine rich protein 1	*Csrp1*	1.53	2.90	4.54E-08	YES	cytoskeleton organization
AI840050	short coiled coil protein	*Scoc*	1.07	2.09	1.47E-04	YES	
AI836541	ribosomal protein L19	*Rpl19*	1.94	3.85	4.38E-12	YES	protein synthesis
AI838741	platelet-activating factor acetylhydrolase, isoform 1b, alpha2 subunit	*Pafah1b2*	1.48	2.79	1.27E-07	YES	other
AI839767	RIKEN cDNA 9130011J04 gene	*9130011J04Rik*	1.11	2.15	8.10E-05	YES	unknown
AI835784	sphingosine-1-phosphate phosphatase 1	*Spph1-pending*	1.37	2.58	1.10E-06	YES	
AI839779	expressed sequence AI839779	*AI839779*	1.07	2.10	1.34E-04	YES	
AI835847	ESTs, Highly similar to NUMM_MOUSE NADH-ubiquinone oxidoreductase 13 kDa-A subunit (Complex I-13KD-A) (CI-13KD-A) [M.musculus]		2.01	4.03	8.04E-13	YES	
AI839755	insulin receptor substrate 1	*Irs1*	1.77	3.40	3.03E-10	YES	signal transducer
AI838775	Mus musculus, clone IMAGE:4196918, mRNA		1.22	2.34	1.27E-05	YES	
AI835864	RIKEN cDNA 2510009N07 gene	*2510009N07Rik*	1.39	2.63	6.95E-07	YES	
AI836574	RIKEN cDNA 2210417O06 gene	*2210417O06Rik*	1.45	2.73	2.46E-07	YES	
AI838798	Mus musculus, clone IMAGE:4952607, mRNA		1.72	3.29	9.16E-10	YES	other
AI835645	formin binding protein 4	*Fnbp4*	1.66	3.16	3.31E-09	YES	
AI839968			1.37	2.59	1.00E-06	YES	
AI843592	ESTs		1.03	2.05	2.30E-04	YES	
AI837089	EST		1.04	2.06	1.98E-04	YES	
AI837136			1.71	3.27	1.09E-09	YES	
AI843395	ubiquitin specific protease 21	*Usp21*	1.05	2.07	1.81E-04	YES	ubiquitin pathway
AI843264	ESTs		1.18	2.26	2.68E-05	YES	

AI842497	RIKEN cDNA 1810060J02 gene	*1810060J02Rik*	1.73	3.32	6.90E-10	YES	
AI837260	expressed sequence AI646709	*AI646709*	1.01	2.01	3.26E-04	YES	
AI843308	RIKEN cDNA 4833447E04 gene	*4833447E04Rik*	1.27	2.41	6.33E-06	YES	
AI837292	phosphodiesterase 1B, Ca2+-calmodulin dependent, 63 kDa	*Pde1b*	1.14	2.20	5.21E-05	YES	signal transducer
AI845440	RIKEN cDNA C330007D15 gene	*C330007D15Rik*	1.39	2.62	7.10E-07	YES	
AI835541	ESTs, Weakly similar to Y37H9A.3.p [Caenorhabditis elegans] [C.elegans]		1.38	2.60	8.63E-07	YES	
AI836096	glial fibrillary acidic protein	*Gfap*	1.22	2.33	1.44E-05	YES	neurogenesis
AI836098	EST		1.57	2.97	2.21E-08	YES	
AI840173	RIKEN cDNA 4632428N05 gene	*4632428N05Rik*	1.42	2.68	4.05E-07	YES	unknown
AI835313	EST		1.18	2.26	2.70E-05	YES	
AI835731	syndecan 4	*Sdc4*	1.20	2.30	1.93E-05	YES	
AI840311	EST		1.98	3.94	1.83E-12	YES	
AI840312			1.06	2.09	1.55E-04	YES	
AI841876	hemoglobin, beta adult minor chain	*Hbb-b2*	1.99	3.98	1.22E-12	YES	transport
AI840316	EST		1.11	2.16	7.18E-05	YES	
AI837996	GRP1 (general receptor for phosphoinositides 1)-associated scaffold protein	*Grasp*	1.61	3.04	1.07E-08	YES	signal transducer
AI841881	RIKEN cDNA 2410008M22 gene	*2410008M22Rik*	1.92	3.79	7.71E-12	YES	
AI837371	RIKEN cDNA 1810005K14 gene	*1810005K14Rik*	1.89	3.72	1.50E-11	YES	
AI837378	ESTs		1.08	2.11	1.21E-04	YES	
AI839541	EST		1.06	2.09	1.52E-04	YES	
AI837380	hypothetical protein MGC36388	*MGC36388*	2.18	4.52	9.10E-15	YES	mitochondria
AI840335	EST		1.32	2.50	2.53E-06	YES	
AI838940	RIKEN cDNA 1110057L18 gene	*1110057L18Rik*	1.98	3.94	1.81E-12	YES	
AI839445	EST, Weakly similar to A42817 proline-rich protein precursor - rat [R.norvegicus]		1.64	3.11	5.25E-09	YES	
AI835968	Ras and Rab interactor 2	*Rin2*	1.15	2.21	4.42E-05	YES	unknown
AI840337			1.11	2.16	7.26E-05	YES	
AI838954	catenin alpha 1	*Catna1*	1.23	2.35	1.17E-05	YES	cell adhesion
AI841536			1.75	3.37	4.29E-10	YES	
AI842688	stathmin-like 2	*Stmb2*	1.29	2.45	4.29E-06	YES	signal transducer
AI845844	striatin, calmodulin binding protein 3	*Strn3*	1.50	2.83	8.50E-08	YES	kinesin complex
AI841136	ESTs		1.22	2.32	1.46E-05	YES	
AI845619	expressed sequence AI845619	*AI845619*	1.13	2.19	5.87E-05	YES	
AI845855	zinc finger protein 289	*Zfp289*	1.57	2.96	2.46E-08	YES	

AI845858	FK506 binding protein 12-rapamycin associated protein 1	*Frap1*	1.42	2.67	4.25E-07	YES	development
AI845861	syntaxin 6	*Stx6*	1.70	3.24	1.47E-09	YES	transport
AI845882	RIKEN cDNA 1110039O14 gene	*1110039O14Rik*	1.61	3.06	8.92E-09	YES	other
AI847181	phosphodiesterase 6G, cGMP-specific, rod, gamma	*Pde6g*	1.35	2.55	1.49E-06	YES	other
AI845888	calcium binding protein P22	*Chp-pending*	1.46	2.76	1.83E-07	YES	unknown
AI843529	EST		1.05	2.07	1.87E-04	YES	
AI835607			1.36	2.57	1.19E-06	YES	
AI840347	EST		1.95	3.87	3.36E-12	YES	
AI838985	cold inducible RNA binding protein	*Cirbp*	1.21	2.31	1.62E-05	YES	
AI839733	guanine nucleotide binding protein, alpha inhibiting 2	*Gnai2*	1.06	2.08	1.65E-04	YES	signal transducer
AI839804	CDC-like kinase 2	*Clk2*	1.01	2.02	3.09E-04	YES	other
AI839739	EST, Weakly similar to TBA1_MOUSE Tubulin alpha-1 chain [M.musculus]		1.49	2.80	1.15E-07	YES	
AI836548	Mus musculus, clone IMAGE:5102170, mRNA, partial cds		1.25	2.38	7.96E-06	YES	other
AI838735	RIKEN cDNA 1810024J13 gene	*1810024J13Rik*	1.18	2.27	2.50E-05	YES	
AI838742	basic FGF repressed, Zic binding protein	*Bfzb-pending*	1.01	2.01	3.41E-04	YES	
AI839766	RIKEN cDNA 5330422J23 gene	*5330422J23Rik*	1.60	3.04	1.15E-08	YES	
AI835779	EST, Weakly similar to BASI_MOUSE Basigin precursor (Basic immunoglobulin superfamily) (Membrane glycoprotein GP42) [M.musculus]		1.86	3.64	3.23E-11	YES	
AI838754	[illegible] 6	*Igfbp6*	1.38	2.60	9.06E-07	YES	growth
AI839777	glycoprotein m6a	*Gpm6a*	1.05	2.07	1.88E-04	YES	
AI839759	vesicle transport through interaction with t-SNAREs 1B homolog	*Vti1b*	1.69	3.23	1.61E-09	YES	transport
AI839760	mitochondrial carrier homolog 1	*Mtch1-pending*	1.41	2.65	5.44E-07	YES	mitochondria
AI839797	RIKEN cDNA 1700008C22 gene	*1700008C22Rik*	1.36	2.57	1.24E-06	YES	
AI835639	ESTs		1.28	2.43	5.22E-06	YES	
AI841660	zinc finger protein 403	*Zfp403-pending*	1.52	2.87	6.12E-08	YES	development
AI839967	expressed sequence AW490886	*AW490886*	1.24	2.35	1.07E-05	YES	neuronal function
AI843597	RIKEN cDNA 4921519L13 gene	*4921519L13Rik*	1.52	2.86	6.64E-08	YES	
AI846328	RIKEN cDNA A230020K05 gene	*A230020K05Rik*	1.07	2.10	1.37E-04	YES	
AI837129	ESTs		1.90	3.74	1.26E-11	YES	
AI842530	expressed sequence AW060394	*AW060394*	1.46	2.76	1.82E-07	YES	
AI845434	EST		1.04	2.06	2.02E-04	YES	

AI845438	RIKEN cDNA 2400007G07 gene	*2400007G07Rik*	1.24	2.36	9.80E-06	YES	
AI845443	numb gene homolog (Drosophila)	*Numb*	1.05	2.07	1.79E-04	YES	neurogenesis
AI838353	ESTs, Weakly similar to MAPB_MOUSE Microtubule-associated protein 1B (MAP 1B) (MAP1.2) (MAP1(X)) [Contains: MAP1 light chain LC1] [M.musculus]		1.31	2.47	3.25E-06	YES	
AI836589	ATP synthase, H+ transporting mitochondrial F1 complex, beta subunit	*Atp5b*	1.34	2.54	1.68E-06	YES	transport
AI839121	EST		1.65	3.13	4.45E-09	YES	
AI835315	Mus musculus, clone IMAGE:3586350, mRNA, partial cds		1.57	2.96	2.36E-08	YES	
AI836597	microtubule-associated protein, RP/EB family, member 2	*Mapre2*	1.04	2.06	2.06E-04	YES	cell cycle
AI839857	similar to hypothetical protein FLJ12949	*LOC215194*	1.26	2.39	7.39E-06	YES	
AI836940	EST		1.51	2.84	8.12E-08	YES	
AI838397	EST, Weakly similar to COP9 subunit 6 (MOV34 homolog, 34 kD) [Homo sapiens] [H.sapiens]		1.44	2.71	2.91E-07	YES	
AI840430	DNA segment, Chr 10, Wayne State University 52, expressed	*D10Wsu52e*	1.09	2.13	1.01E-04	YES	
AI840468	ESTs		1.58	2.99	1.83E-08	YES	
AI835378	ESTs		1.33	2.52	1.97E-06	YES	
AI838630	RIKEN cDNA 1110021H02 gene	*1110021H02Rik*	1.18	2.27	2.54E-05	YES	
AI839652	t-complex protein 1, related sequence 1	*Tcp1-rs1*	1.22	2.33	1.43E-05	YES	
AI839320	EST		1.20	2.30	1.78E-05	YES	
AI839323	EST		1.08	2.12	1.13E-04	YES	
AI837335	EST		1.78	3.42	2.53E-10	YES	
AI837337	hypothetical protein MGC37950	*MGC37950*	1.07	2.10	1.37E-04	YES	
AI845489	fatty acid binding protein 7, brain	*Fabp7*	1.12	2.18	6.44E-05	YES	transport
AI845521	myosin VIIA and Rab interacting protein	*Myrip*	1.62	3.08	7.37E-09	YES	transport
AI845533	RIKEN cDNA 2310003C10 gene	*2310003C10Rik*	1.32	2.50	2.53E-06	YES	cell cycle
AI834863	ribosomal protein L26	*Rpl26*	1.81	3.50	1.18E-10	YES	protein synthesis
AI846741	EST		1.11	2.16	7.28E-05	YES	
AI846790			1.03	2.04	2.49E-04	YES	
AI836669	RIKEN cDNA 6330527O06 gene	*6330527O06Rik*	1.12	2.17	6.50E-05	YES	
AI839874	Carbonic anhydrase 8	*Car8*	1.56	2.95	2.78E-08	YES	carbonate metabolism
AI838406			1.38	2.61	8.19E-07	YES	
AI838412	clathrin, heavy polypeptide (Hc)	*Cltc*	1.01	2.01	3.32E-04	YES	

AI835993	EST		1.79	3.46	1.84E-10	YES	
AI836866	ESTs		1.23	2.35	1.12E-05	YES	
AI836072	ESTs		1.17	2.25	3.04E-05	YES	
AI841812	expressed sequence AI043088	*AI043088*	1.94	3.83	5.13E-12	YES	
AI834934	RIKEN cDNA 1700084G18 gene	*1700084G18Rik*	1.47	2.76	1.74E-07	YES	
AI842966	RIKEN cDNA 1110019I12 gene	*1110019I12Rik*	1.93	3.82	5.51E-12	YES	
AI846449	EST		1.36	2.56	1.30E-06	YES	
AI842979	RIKEN cDNA 2810430J06 gene	*2810430J06Rik*	1.52	2.87	6.12E-08	YES	
AI842981	RIKEN cDNA 2810407B07 gene	*2810407B07Rik*	1.26	2.39	7.39E-06	YES	
AI840930	ESTs		1.94	3.83	5.05E-12	YES	
AI843570	ESTs		1.65	3.13	4.59E-09	YES	
AI835689	expressed sequence AI835689	*AI835689*	1.22	2.32	1.49E-05	YES	neurogenesis
AI839854	2	*Copz2*	1.32	2.50	2.42E-06	YES	transport
AI836600	stimulated by retinoic acid 13	*Stra13*	1.05	2.07	1.89E-04	YES	stress response
AI838290	tumors	*Armet*	1.49	2.80	1.15E-07	YES	
AI838295	RIKEN cDNA 3010033P07 gene	*3010033P07Rik*	1.12	2.18	6.38E-05	YES	protein synthesis
AI839125	RIKEN cDNA 3100001N19 gene	*3100001N19Rik*	1.32	2.50	2.44E-06	YES	protein synthesis
AI839134	eukaryotic translation initiation factor 3, subunit 3 (gamma, 40kD)	*Eif3s3*	1.44	2.72	2.65E-07	YES	protein synthesis
AI838328	EST		1.21	2.32	1.58E-05	YES	
AI840275	protein COII [Mus musculus] [M.musculus]		1.58	2.98	1.93E-08	YES	
AI836220	expressed sequence AI848120	*AI848120*	1.12	2.17	7.02E-05	YES	
AI839634			1.56	2.95	2.56E-08	YES	
AI840425	EST		1.70	3.25	1.41E-09	YES	
AI838591	actin, beta, cytoplasmic	*Actb*	1.11	2.15	8.17E-05	YES	cytoskeleton organization
AI836623	RIKEN cDNA 1100001F19 gene	*1100001F19Rik*	1.53	2.88	5.44E-08	YES	ubiquitin pathway
AI845753	RIKEN cDNA 2810422J05 gene	*2810422J05Rik*	1.97	3.92	2.16E-12	YES	
AI843781	protein phosphatase 3, catalytic subunit, alpha isoform	*Ppp3ca*	2.09	4.25	1.01E-13	YES	
AI845764	EST		1.38	2.60	8.85E-07	YES	
AI845485	four and a half LIM domains 4	*Fhl4*	1.40	2.65	5.59E-07	YES	transport
AI845491	microsomal glutathione S-transferase 1	*Mgst1*	1.31	2.49	2.88E-06	YES	mitochondria
AI845532	gene family, member C (S. cerevisiae), clone IMAGE:4950601, mRNA, partial cds		1.72	3.29	9.59E-10	YES	other
AI843221	tweety homolog 2 (Drosophila)	*Ttyh2*	1.33	2.51	2.25E-06	YES	
AI842586	troponin T2, cardiac	*Tnnt2*	1.31	2.48	2.88E-06	YES	

AI846743	transgelin	*Tagln*	1.72	3.29	9.28E-10	YES	development
AI846795	EST		1.75	3.36	4.72E-10	YES	
AI836665	5730493B19 [Mus musculus] [M.musculus]		1.17	2.25	3.08E-05	YES	
AI838669	proteasome (prosome, macropain) 26S subunit, non-ATPase, 12	*Psmd12*	1.46	2.75	2.08E-07	YES	proteasome
AI838428			1.43	2.70	3.25E-07	YES	
AI838866	EST		1.26	2.40	7.04E-06	YES	
AI836471	EST		1.12	2.18	6.47E-05	YES	
AI837029	reticulocalbin	*Rcn*	1.74	3.35	5.13E-10	YES	
AI841447			1.14	2.21	4.78E-05	YES	
AI837051	RIKEN cDNA 2310012I15 gene	*2310012I15Rik*	1.07	2.10	1.36E-04	YES	transport
AI841802	seryl-aminoacyl-tRNA synthetase 1	*Sars1*	1.42	2.67	4.24E-07	YES	other
AI840133	ESTs		1.42	2.67	4.25E-07	YES	signal transducer
AI841809	SMT3 (supressor of mif two, 3) homolog 1 (S. cerevisiae)	*Smt3h1*	1.17	2.25	2.96E-05	YES	
AI839067	RAN binding protein 1	*Ranbp1*	1.51	2.85	7.04E-08	YES	signal transducer
AI837689	mitochondrial ribosomal protein L12	*Mrpl12*	1.20	2.30	1.95E-05	YES	mitochondria
AI846518	transferrin receptor	*Trfr*	1.18	2.26	2.74E-05	YES	signal transducer
AI843553	heat shock 70kD protein 5 (glucose-regulated protein, 78kD)	*Hspa5*	1.64	3.11	5.47E-09	YES	heat shock response
AI843557	EST		1.17	2.25	3.00E-05	YES	
AI842099	protein kinase C, alpha	*Prkca*	1.59	3.01	1.48E-08	YES	signal transducer
AI844802	zinc finger protein 238	*Zfp238*	1.01	2.01	3.27E-04	YES	transcription regulator
AI847888	kinesin light chain 2	*Klc2*	1.24	2.36	9.68E-06	YES	neuronal function
AI844942	ras homolog gene family, member U	*Arhu*	1.23	2.34	1.19E-05	YES	cell cycle
AI852203	expressed sequence AW552276	*AW552276*	1.04	2.05	2.22E-04	YES	
AI852227	Mus musculus, clone IMAGE:3584069, mRNA, partial cds		1.14	2.21	4.62E-05	YES	
AI853523	RIKEN cDNA 0710008D09 gene	*0710008D09Rik*	1.27	2.41	6.13E-06	YES	mitochondria
AI852526	G-protein coupled receptor 88	*Gpr88*	1.05	2.07	1.78E-04	YES	signal transducer
AI848082	ESTs, Weakly similar to T42731 atrophin-1 related protein - rat [R.norvegicus]		1.19	2.29	2.15E-05	YES	
AI848402	histocompatibility 2, T region locus 22	*H2-T22*	1.37	2.59	9.90E-07	YES	immune response
AI848093	RIKEN cDNA 1110005A03 gene	*1110005A03Rik*	1.02	2.03	2.71E-04	YES	
AI846131	RIKEN cDNA 1110002L01 gene	*1110002L01Rik*	1.07	2.10	1.43E-04	YES	
AI842141	RIKEN cDNA 0610013I17 gene	*0610013I17Rik*	1.19	2.28	2.22E-05	YES	transcription regulator
AI848454	RIKEN cDNA 2010012C09 gene	*2010012C09Rik*	1.06	2.09	1.58E-04	YES	

AI848468	associated with Prkcl1	*Awp1-pending*	1.04	2.06	2.12E-04	YES	signal transducer
AI842433	myeloid-associated differentiation marker	*Myadm*	1.22	2.33	1.43E-05	YES	
AI849487	EST		1.71	3.27	1.09E-09	YES	
AI852556	expressed sequence AW495222	*AW495222*	1.13	2.18	5.89E-05	YES	
AI850771	EST		1.41	2.65	5.43E-07	YES	
AI851985	brain abundant, membrane attached signal protein 1	*Basp1*	1.51	2.84	7.95E-08	YES	transcription regulator
AI852023	RIKEN cDNA 6330415M09 gene	*6330415M09Rik*	1.09	2.13	1.00E-04	YES	signal transducer
AI850565	pleiotrophin	*Ptn*	1.39	2.62	7.03E-07	YES	growth
AI847366	nuclear factor, erythroid derived 2,-like 1	*Nfe2l1*	1.44	2.71	2.86E-07	YES	transcription regulator
AI844503	GTP cyclohydrolase 1	*Gch*	1.23	2.34	1.22E-05	YES	transport
AI848724			1.05	2.07	1.91E-04	YES	
AI847553	RIKEN cDNA 6530406A20 gene	*6530406A20Rik*	1.13	2.19	5.79E-05	YES	
AI852649	dynactin 5	*Dctn5*	1.31	2.48	3.09E-06	YES	
AI852521	endothelial differentiation, sphingolipid G-protein-coupled receptor, 8	*Edg8*	2.01	4.03	7.77E-13	YES	signal transducer
AI853528	ESTs, Weakly similar to R04E5.8.p [Caenorhabditis elegans] [C.elegans]		1.40	2.64	5.88E-07	YES	
AI834942	ESTs		1.30	2.47	3.41E-06	YES	
AI834945	mesoderm specific transcript	*Mest*	1.54	2.92	3.70E-08	YES	xenobiotic metabolism
AI848094	DnaJ (Hsp40) homolog, subfamily C, member 8	*Dnajc8*	1.01	2.02	3.07E-04	YES	
AI846133	expressed sequence AI846133	*AI846133*	1.29	2.45	4.26E-06	YES	
AI846834	pleckstrin homology domain-containing, family A (phosphoinositide binding specific) member 3	*Plekha3*	1.56	2.94	2.88E-08	YES	phospholipid metabolism
AI846847	Mus musculus, Similar to CGI-49 protein, clone MGC:36578 IMAGE:5100552, mRNA, complete cds		1.47	2.77	1.55E-07	YES	
AI851954	mitochondrial ribosomal protein L49	*Mrpl49*	1.32	2.49	2.71E-06	YES	
AI852315			1.02	2.02	2.92E-04	YES	
AI848921	ESTs		-1.51	-2.85	7.26E-08	YES	
AI844003	RIKEN cDNA 4930566A11 gene	*4930566A11Rik*	1.66	3.15	3.62E-09	YES	
AI850654	immediate early response, erythropoietin 1	*Ierepo1-pending*	1.01	2.02	2.99E-04	YES	transcription regulator
AI851828	EST		1.06	2.08	1.67E-04	YES	
AI854433	pregnancy upregulated non-ubiquitously expressed CaM kinase	*Pnck*	1.33	2.51	2.23E-06	YES	other

AI850250	Splicing factor, proline-and glutamine-rich (Polypyrimidine tract-binding protein-associated splicing factor) (PTB-associated splicing factor) (PSF) (DNA-binding P52/P100 complex, 100 kDa subunit) [H.sapiens]		1.05	2.07	1.87E-04	YES	
AI850252	RIKEN cDNA 6720463E02 gene	*6720463E02Rik*	1.06	2.08	1.62E-04	YES	other
AI844955			1.10	2.14	9.19E-05	YES	
AI850305	expressed sequence AI850305	*AI850305*	1.15	2.22	4.06E-05	YES	
AI847913	profilin 2	*Pfn2*	1.63	3.10	6.10E-09	YES	cytoskeleton organization
AI848282	expressed sequence AI848282	*AI848282*	1.06	2.08	1.67E-04	YES	
AI852781	expressed sequence AW556797	*AW556797*	1.71	3.27	1.09E-09	YES	transport
AI852921	metal response element binding transcription factor 1	*Mtf1*	1.29	2.44	4.63E-06	YES	transcription regulator
AI846231	peptidyl arginine deiminase, type II	*Pdi2*	1.04	2.05	2.20E-04	YES	other
AI848623	solute carrier family 6 (neurotransmitter transporter, taurine), member 6	*Slc6a6*	1.08	2.12	1.12E-04	YES	neuronal function
AI844243	f-box and WD-40 domain protein 5	*Fbxw5*	-1.06	-2.08	1.59E-04	YES	ubiquitin pathway
AI854434	ribosomal protein L13a	*Rpl13a*	2.25	4.75	1.11E-15	YES	development
AI854437	RIKEN cDNA 2010317E03 gene	*2010317E03Rik*	1.06	2.08	1.66E-04	YES	other
AI850253	Mus musculus, clone IMAGE:4457493, mRNA		1.05	2.07	1.80E-04	YES	
AI852095	RIKEN cDNA 5730536A07 gene	*5730536A07Rik*	1.70	3.25	1.34E-09	YES	
AI847845	protocadherin alpha 1	*Pcdha1*	-1.05	-2.06	1.96E-04	YES	cell adhesion
AI847865	transcription factor 1	*Tcf1*	1.14	2.20	5.10E-05	YES	transcription regulator
AI842821	phospholipase C-like 2	*Plcl2*	1.10	2.15	8.55E-05	YES	signal transducer
AI853984	expressed sequence C76391	*C76391*	-1.28	-2.43	5.22E-06	YES	
AI853987	expressed sequence AI593252	*AI593252*	1.02	2.02	2.90E-04	YES	
AI853996	RAB11a, member RAS oncogene family	*Rab11a*	-1.11	-2.16	7.72E-05	YES	signal transducer
AI849757			-1.27	-2.41	6.24E-06	YES	
AI852475	[illegible] D	*Hnrpd*	1.09	2.13	1.01E-04	YES	RNA processing
AI844560	mitogen activated protein binding protein interacting protein	*Mapbpip-pending*	1.46	2.74	2.16E-07	YES	other
AI852301	glutamate dehydrogenase	*Glud*	1.02	2.03	2.85E-04	YES	mitochondria
AI850099	RIKEN cDNA 2900002L20 gene	*2900002L20Rik*	1.09	2.13	9.86E-05	YES	unknown
AI850551	ubiquitin fusion degradation 1 like	*Ufd1l*	1.28	2.43	5.18E-06	YES	ubiquitin pathway
AI850598	hypothetical protein MGC37551	*MGC37551*	1.19	2.28	2.30E-05	YES	
AI848163	ESTs		1.80	3.47	1.58E-10	YES	

AI844450	hypothetical protein, I54	*X61497*	1.26	2.40	6.96E-06	YES	
AI842820	RIKEN cDNA 5730456K23 gene	*5730456K23Rik*	-1.20	-2.30	1.83E-05	YES	
AI842822	RIKEN cDNA 1110003F06 gene	*1110003F06Rik*	1.94	3.84	4.64E-12	YES	
AI844481	Mus musculus, Similar to hypothetical protein, clone MGC:27918 IMAGE:3582119, mRNA, complete cds		1.21	2.32	1.51E-05	YES	unknown
AI846978	leucine rich repeat (in FLII) interacting protein 2	*Lrrfip2*	-1.56	-2.96	2.48E-08	YES	contains DNA repair motif
AI847514	solute carrier family 1, member 3	*Slc1a3*	-1.52	-2.86	6.57E-08	YES	transport
AI847521	ESTs		-1.33	-2.51	2.27E-06	YES	
AI849136	EST		1.07	2.10	1.41E-04	YES	
AI853516	expressed sequence 2 embryonic lethal	*Es2el*	1.13	2.18	6.04E-05	YES	
AI845987	RIKEN cDNA 0610009E20 gene	*0610009E20Rik*	1.16	2.24	3.51E-05	YES	unknown
AI844569	RIKEN cDNA 1100001J08 gene	*1100001J08Rik*	1.07	2.10	1.44E-04	YES	
AI848803	FK506 binding protein 8 (38 kDa)	*Fkbp8*	1.17	2.25	2.98E-05	YES	unknown
AI846869	neuraminidase 1	*Neu1*	1.08	2.12	1.17E-04	YES	carbohydrate metabolism
AI852570	expressed sequence AI450326	*AI450326*	1.08	2.11	1.18E-04	YES	
AI849956	EST		1.18	2.26	2.76E-05	YES	
AI852261	NS1-associated protein 1	*Nsap1-pending*	1.10	2.14	9.31E-05	YES	RNA processing
AI851896	protein tyrosine phosphatase, receptor type, L	*Ptprl*	-1.22	-2.34	1.29E-05	YES	cell adhesion
AI850599	transcriptional regulating protein 132, isoform 2; rapa-2; rapa-1 [Homo sapiens] [H.sapiens]		1.05	2.07	1.83E-04	YES	
AI847692	EST		1.06	2.08	1.69E-04	YES	
AI847694	mitogen-activated protein kinase 8 interacting protein 2	*Mapk8ip2*	1.42	2.68	4.10E-07	YES	signal transducer
AI849361	RIKEN cDNA 1700056O17 gene	*1700056O17Rik*	1.72	3.29	9.24E-10	YES	
AI848275	RIKEN cDNA 1810045K07 gene	*1810045K07Rik*	1.01	2.02	3.09E-04	YES	unknown
AI844073	hypothetical protein MGC38847	*MGC38847*	1.04	2.05	2.19E-04	YES	unknown
AI853386	EST		1.95	3.85	4.09E-12	YES	
AI851815	RIKEN cDNA 0610025L15 gene	*0610025L15Rik*	1.07	2.09	1.46E-04	YES	
AI850723	calmodulin 2	*Calm2*	1.39	2.61	7.85E-07	YES	cell cycle
AI851852	ESTs		1.76	3.39	3.46E-10	YES	
AI852958	ESTs, Weakly similar to pregnancy upregulated non-ubiquitously expressed CaM kinase; calcium/calmodulin-dependent protein kinase 1, beta [Mus musculus] [M.musculus]		1.31	2.48	3.05E-06	YES	

AI847398	expressed sequence AW049283	*AW049283*	1.33	2.52	2.01E-06	YES	unknown
AI849672	expressed sequence AI414849	*AI414849*	1.00	2.01	3.44E-04	YES	
AI848053	expressed sequence AL024256	*AL024256*	1.03	2.04	2.48E-04	YES	
AI848056	RIKEN cDNA 2700079K05 gene	*2700079K05Rik*	1.29	2.45	4.05E-06	YES	
AI845223	fibroblast growth factor 12	*Fgf12*	1.07	2.10	1.40E-04	YES	neuronal function
AI847905	(isopeptidase T)	*Usp5*	1.38	2.60	9.43E-07	YES	ubiquitin pathway
AI847695	kinesin heavy chain member 1A	*Kif1a*	1.23	2.35	1.08E-05	YES	neuronal function
AI848239	kinase 2	*Srpk2*	1.05	2.08	1.72E-04	YES	other
AI849325	gamma	*Idh3g*	1.57	2.97	2.26E-08	YES	mitochondria
AI847697	RIKEN cDNA 2010110M21 gene	*2010110M21Rik*	1.26	2.40	7.02E-06	YES	unknown
AI848266	transgelin 3	*Tagln3*	1.10	2.14	9.13E-05	YES	neurogenesis
AI849357	expressed sequence AI413391	*AI413391*	1.43	2.69	3.79E-07	YES	
AI844129	cDNA sequence BC017158	*BC017158*	1.18	2.26	2.82E-05	YES	
AI850648	RIKEN cDNA 2210403N08 gene	*2210403N08Rik*	1.68	3.21	1.95E-09	YES	
AI850692	isoprenylcysteine carboxyl methyltransferase	*Icmt*	1.10	2.14	9.01E-05	YES	other
AI850194	Unc-51 like kinase 1 (C. elegans)	*Ulk1*	1.80	3.47	1.58E-10	YES	protein modification
AI848392	expressed sequence AI848392	*AI848392*	1.12	2.17	6.73E-05	YES	
AI847438	ESTs, Moderately similar to S12207 hypothetical protein (B2 element) - mouse [M.musculus]		1.05	2.07	1.81E-04	YES	
AI854481	EST		1.28	2.42	5.40E-06	YES	
AI851613	expressed sequence AI851613	*AI851613*	1.17	2.25	3.13E-05	YES	
AI851636	formin 2	*Fmn2*	1.22	2.33	1.39E-05	YES	development
AI853573	ESTs, Highly similar to HAPP_RAT Huntingtin-associated protein-interacting protein (DUO protein) (Kalirin) (PAM COOH-terminal interactor protein 10) (P-CIP10) [R.norvegicus]		1.03	2.05	2.28E-04	YES	
AI528658	DEAD/H (Asp-Glu-Ala-Asp/His) box polypeptide 13 (RNA helicase A)	*Ddx24*	1.09	2.13	1.00E-04	YES	RNA processing
AI854523	RIKEN cDNA 3110006P09 gene	*3110006P09Rik*	1.15	2.23	3.94E-05	YES	unknown
AI323871	cyclin D3	*Ccnd3*	1.05	2.06	1.96E-04	YES	cell cycle
AI851386	EST		-1.41	-2.65	5.49E-07	YES	
BG088344	RIKEN cDNA 4930586I02 gene	*4930586I02Rik*	-1.13	-2.19	5.48E-05	YES	
BG076127	expressed sequence AU021063	*AU021063*	1.25	2.37	8.99E-06	YES	
BG088348	cDNA sequence BC032200	*BC032200*	1.22	2.33	1.36E-05	YES	
BG075824	ESTs, Weakly similar to S24407 formin isoform IV - mouse [M.musculus]		-1.19	-2.28	2.37E-05	YES	

BG088369	RIKEN cDNA 2010004M01 gene	*2010004M01Rik*	-1.06	-2.08	1.60E-04	YES	
AW556391	ribosomal protein L32	*Rpl32*	1.20	2.30	1.84E-05	YES	protein synthesis
BG075844	ESTs		-1.30	-2.46	3.83E-06	YES	
BG075544	ESTs		-1.10	-2.15	8.65E-05	YES	
BG088092	solute carrier family 14 (urea transprorter), member 1	*Slc14a1*	-1.43	-2.70	3.24E-07	YES	transport
BG088094	succinate-Coenzyme A ligase, GDP-forming, beta subunit	*Suclg2*	-1.44	-2.72	2.71E-07	YES	mitochondria
BG076200	DAZ associated protein 1	*Dazap1*	1.27	2.41	5.95E-06	YES	mitochondria
AI854124	RIKEN cDNA 1810010H24 gene	*1810010H24Rik*	1.20	2.30	1.86E-05	YES	
AI854696	RIKEN cDNA 2610002J23 gene	*2610002J23Rik*	1.20	2.30	1.79E-05	YES	
AI854698	ESTs		-1.03	-2.05	2.26E-04	YES	
BG075908	epithelial protein lost in neoplasm	*Eplin-pending*	1.56	2.95	2.67E-08	YES	unknown
AI327009	RIKEN cDNA 0610025G13 gene	*0610025G13Rik*	1.18	2.26	2.83E-05	YES	protein synthesis
AI415231	ESTs, Weakly similar to F-box protein FBL2 [Rattus norvegicus] [R.norvegicus]		1.10	2.14	9.34E-05	YES	
AI854856	ESTs		-1.09	-2.13	1.01E-04	YES	
AI854892	RIKEN cDNA 2900009I07 gene	*2900009I07Rik*	1.27	2.41	5.89E-06	YES	
AI852836	RIKEN cDNA 5530600M07 gene	*5530600M07Rik*	1.10	2.14	9.33E-05	YES	
AI326150	centrin 2	*Cetn2*	1.15	2.21	4.48E-05	YES	cell cycle
AI854692	Mus musculus, clone IMAGE:3964267, mRNA		1.12	2.17	6.59E-05	YES	signal transducer
AI850919	ESTs		1.06	2.08	1.59E-04	YES	
AI323923	carbonyl reductase 1	*Cbr1*	1.17	2.24	3.30E-05	YES	other
BG085134	CD24a antigen	*Cd24a*	1.52	2.87	8.39E-10	YES	immune response
BG072306	5730493B19 [Mus musculus] [M.musculus]		1.56	2.96	2.89E-10	YES	
BG072622	ESTs		1.06	2.09	1.84E-05	YES	
BG072324	ESTs, Highly similar to EVI1_MOUSE Ecotropic virus integration 1 site protein [M.musculus]		1.58	2.99	1.86E-10	YES	development
BG072348	ESTs, Weakly similar to MAP-1 protein [Mus musculus] [M.musculus]		1.60	3.03	1.09E-10	YES	transport
BG085187	neurochondrin	*Ncdn-pending*	1.21	2.32	1.00E-06	YES	(purkinje cell specific)
BG087265	hepatoma-derived growth factor, related protein 3	*Hdgfrp3*	1.33	2.52	7.79E-08	YES	cell proliferation
AW554918	expressed sequence AW554918	*AW554918*	1.00	2.00	5.46E-05	YES	

BG072986	RIKEN cDNA 3110080J08 gene	*3110080J08Rik*	1.02	2.03	3.74E-05	YES	
BG065423	ESTs		-1.06	-2.09	1.90E-05	YES	
BG076807	RIKEN cDNA 1810029F08 gene	*1810029F08Rik*	1.47	2.76	3.29E-09	YES	
BG085385	hypothetical protein MGC37079	*MGC37079*	-1.29	-2.45	1.86E-07	YES	
BG073897	Mus musculus, clone IMAGE:3371882, mRNA, partial cds		-1.00	-2.01	5.13E-05	YES	
BG074001	ESTs		-1.07	-2.11	1.46E-05	YES	
BG065758	Riken clone E330007O21	*RIKE330007O21*	1.16	2.23	3.15E-06	YES	unknown
BG066243	SET and MYND domain containing 1	*Smyd1*	1.13	2.20	4.72E-06	YES	development
BG064399	RIKEN cDNA 0610007L01 gene	*0610007L01Rik*	1.14	2.20	4.61E-06	YES	
AW544570	carbonic anhydrase 2	*Car2*	1.18	2.27	1.83E-06	YES	other
AW544597	Mus musculus, clone IMAGE:3482828, mRNA		1.45	2.73	5.08E-09	YES	
BG077775	tumor necrosis factor receptor superfamily, member 23	*Tnfrsf23*	1.65	3.13	2.99E-11	YES	transport
BG077779	copine III	*Cpne3*	1.69	3.22	1.04E-11	YES	transport
AW544721	RIKEN cDNA 1500004D14 gene	*1500004D14Rik*	1.66	3.15	2.36E-11	YES	
AW544734	expressed sequence AU021107	*AU021107*	1.46	2.76	3.52E-09	YES	
AW539454			1.09	2.13	1.03E-05	YES	
BG067079	expressed sequence AU015422	*AU015422*	-1.20	-2.30	1.29E-06	YES	
BG070057	ESTs		-1.07	-2.09	1.74E-05	YES	
BG069433	ESTs		1.28	2.43	2.24E-07	YES	
BG080632	Scm-related gene containing four mbt domains	*Sfmbt*	-1.16	-2.23	2.88E-06	YES	
BG069818	ubiquitin specific protease 3	*Usp3*	-1.28	-2.43	2.27E-07	YES	ubiquitin pathway
BG069820	RIKEN cDNA 5330431K02 gene	*5330431K02Rik*	-1.37	-2.58	3.57E-08	YES	
BG069824	expressed sequence AU021092	*AU021092*	-1.05	-2.07	2.33E-05	YES	
BG069826	expressed sequence AW557704	*AW557704*	-1.52	-2.88	7.99E-10	YES	
BG067062	ESTs, Moderately similar to S12207 hypothetical protein (B2 element) - mouse [M.musculus]		-1.36	-2.56	4.53E-08	YES	
BG080472	WD repeat domain 5	*Wdr5*	-1.64	-3.11	4.24E-11	YES	development
BG081218	DNA cross-link repair 1A, PSO2 homolog (S. cerevisiae)	*Dclre1a*	-1.09	-2.13	1.13E-05	YES	DNA repair
BG069248	trans-acting transcription factor 6	*Sp6*	1.07	2.10	1.60E-05	YES	transcription regulator
BG081126	Mus musculus, Similar to FAT tumor suppressor (Drosophila) homolog, clone IMAGE:4954893, mRNA		-1.11	-2.16	7.83E-06	YES	

BG072723	Mus musculus, clone IMAGE:1230948, mRNA, partial cds	1.10	2.14	9.71E-06	YES	
AW546889		-1.01	-2.02	4.25E-05	YES	

a NCBI GenBank database (http://www.ncbi.nlm.nih.gov/) was used to obtain gene name, gene symbol, and summary function

Table S2 Changes in Gene Expression Associated with HN2 treatment in Cultured Mouse Neurons*							
			HN2/Control				
Accession ID	Gene Name	Gene Symbol	Ratio (Log2)	Fold change	unadjusted p (z test using pooled error estimate)	Statistically significant (adjusted for multiple comparisons)	Summary Function
AI854261	calcium channel, voltage-dependent, alpha2/delta subunit 1	*Cacna2d1*	-1.32	-2.49	1.39E-06	YES	transport
BG075976	ESTs		1.28	2.44	2.49E-06	YES	
AI415416	solute carrier family 20, member 2	*Slc20a2*	1.01	2.01	2.18E-04	YES	signal transducer
AI853048	ESTs		1.01	2.01	2.22E-04	YES	
AI854758	ribosomal protein L27	*Rpl27*	-1.16	-2.24	2.05E-05	YES	protein synthesis
BG088908	RIKEN cDNA 3110038L01 gene	*3110038L01Rik*	1.09	2.14	6.06E-05	YES	
AI414701	ESTs		1.09	2.13	6.17E-05	YES	
AW559151			1.11	2.16	4.70E-05	YES	
AI854256	UDP-N-acetyl-alpha-D-galactosamine:polypeptide N-acetylgalactosaminyltransferase 9	*Galnt9*	-1.21	-2.32	9.00E-06	YES	other
AW555822			1.36	2.57	6.13E-07	YES	
BG075669	RIKEN cDNA 2700023B17 gene	*2700023B17Rik*	1.13	2.18	3.70E-05	YES	
AW557790			1.05	2.07	1.15E-04	YES	
AW555902			1.63	3.10	2.29E-09	YES	
AW556849			1.10	2.15	5.34E-05	YES	
BG088532	RIKEN cDNA 2610313E07 gene	*2610313E07Rik*	1.00	2.01	2.33E-04	YES	unknown
AI415710	expressed sequence AI120141	*AI120141*	-1.04	-2.05	1.44E-04	YES	unknown
AI851097	expressed sequence AW060654	*AW060654*	-1.24	-2.36	5.59E-06	YES	unknown
AW559004			1.37	2.58	5.32E-07	YES	

AW558121	RIKEN cDNA 2310058A11 gene	*2310058A11Rik*	1.12	2.17	4.27E-05	YES	
AW558174	EST		1.33	2.51	1.10E-06	YES	
AI838008	glyoxylate reductase/hydroxypyruvate reductase	*Grhpr*	1.20	2.30	1.09E-05	YES	other
AI839520	guanylate kinase 1	*Guk1*	1.12	2.18	3.84E-05	YES	other
AI835905	ferritin heavy chain	*Fth*	1.13	2.19	3.58E-05	YES	transport
AI836319	EST		1.79	3.45	6.02E-11	YES	
AI835438	ESTs		1.09	2.13	6.66E-05	YES	
AI836814	EST		1.60	3.03	4.70E-09	YES	
AI838922	EST		1.18	2.27	1.48E-05	YES	
AI845823	Mus musculus, similar to Unknown (protein for IMAGE:3610257), clone MGC:37326 IMAGE:4975562, mRNA, complete cds		1.27	2.41	3.27E-06	YES	
AI838959	actin, alpha 2, smooth muscle, aorta	*Acta2*	1.10	2.14	5.52E-05	YES	cytoskeleton organization
AI840342	EST		1.37	2.58	5.18E-07	YES	
AI837442			1.32	2.50	1.25E-06	YES	
AI836538	EST		1.21	2.31	9.15E-06	YES	
AI838727	EST		1.18	2.27	1.45E-05	YES	
AI838763	expressed sequence AI838763	*AI838763*	1.24	2.36	5.42E-06	YES	
AI838773	expressed sequence AI838773	*AI838773*	1.22	2.32	8.40E-06	YES	
AI841636	RIKEN cDNA 1810021J13 gene	*1810021J13Rik*	1.11	2.16	4.74E-05	YES	unknown
AI840852	EST		1.41	2.65	2.49E-07	YES	
AI843543	RIKEN cDNA 2610037M15 gene	*2610037M15Rik*	1.09	2.13	6.44E-05	YES	unknown
AI843670	RIKEN cDNA A230106A15 gene	*A230106A15Rik*	1.25	2.38	4.64E-06	YES	
AI840542			1.78	3.43	7.28E-11	YES	
AI846342	membrane-type frizzled-related protein	*Mfrp*	1.26	2.39	3.94E-06	YES	development

AI843257	hypothetical protein MGC36831	*MGC36831*	1.29	2.45	2.16E-06	YES	
AI846359	cAMP responsive element modulator	*Crem*	1.23	2.34	7.02E-06	YES	transcription regulator
AI842511			1.06	2.08	1.08E-04	YES	
AI837833	zinc finger protein 95	*Zfp95*	1.51	2.86	2.83E-08	YES	transcription regulator
AI835726	RIKEN cDNA 1810060D16 gene	*1810060D16Rik*	1.03	2.05	1.51E-04	YES	transcription
AI838024	Mus musculus, clone IMAGE:1447438, mRNA, partial cds		1.05	2.07	1.24E-04	YES	
AI841920	expressed sequence AL022641	*AL022641*	-1.25	-2.37	4.85E-06	YES	transport
AI837396	EST		1.62	3.08	2.76E-09	YES	
AI840334	EST, Weakly similar to beta-tubulin cofactor C; tubulin-specific chaperone c [Homo sapiens] [H.sapiens]		1.42	2.68	1.87E-07	YES	
AI842684	interferon regulatory factor 3	*Irf3*	-1.07	-2.10	8.87E-05	YES	transcription regulator
AI845582	sparc/osteonectin, cwcv and kazal-like domains proteoglycan 1	*Spock1*	1.07	2.11	8.20E-05	YES	unknown
AI845621	striatin, calmodulin binding protein 4	*Strn4*	1.05	2.07	1.24E-04	YES	other
AI840919	DNA-damage inducible transcript 3	*Ddit3*	1.33	2.51	1.13E-06	YES	apoptosis control
AI845870	EST		1.38	2.60	4.30E-07	YES	
AI840807	beta-spectrin 4	*Spnb4*	1.26	2.40	3.85E-06	YES	scytoskeleton
AI836047	ESTs		1.30	2.47	1.78E-06	YES	
AI837935	EST		1.58	2.99	7.35E-09	YES	
AI840047	EST		1.34	2.53	8.78E-07	YES	
AI839815	EST		1.35	2.55	7.65E-07	YES	
not yet submitted			1.10	2.14	5.68E-05	YES	
AI840113	peroxiredoxin 2	*Prdx2*	1.25	2.38	4.67E-06	YES	apoptosis control

AI841630	ATP citrate lyase	*Acly*	1.13	2.19	3.56E-05	YES	other
AI841643	platelet derived growth factor, B polypeptide	*Pdgfb*	1.35	2.54	8.05E-07	YES	cell cycle
AI843335	expressed sequence AU040105	*AU040105*	1.15	2.22	2.53E-05	YES	
AI843538	ESTs		1.11	2.16	4.66E-05	YES	
AI843248	RIKEN cDNA 5730405I09 gene	*5730405I09Rik*	1.17	2.24	1.92E-05	YES	cell cycle
AI843286			1.39	2.62	3.56E-07	YES	
AI843291	synbindin	*Sbdn*	1.11	2.16	4.73E-05	YES	transport
AI843127	huntingtin interacting protein 2	*Hip2*	1.08	2.11	7.68E-05	YES	ubiquitin pathway
AI843131	RIKEN cDNA 3110040D16 gene	*3110040D16Rik*	1.88	3.69	4.99E-12	YES	
AI842901	Mus musculus, Similar to fatty acid hydroxylase, clone IMAGE:4208853, mRNA, partial cds		1.11	2.15	5.03E-05	YES	
AI528719	guanosine diphosphate (GDP) dissociation inhibitor 3	*Gdi3*	1.29	2.44	2.39E-06	YES	signal transducer
AI854089			-2.15	-4.44	3.33E-15	YES	
AI854901	RIKEN cDNA 2510006D16 gene	*2510006D16Rik*	-1.44	-2.72	1.24E-07	YES	
BG076138	ESTs		1.12	2.18	3.76E-05	YES	
AI413648	ESTs		-1.19	-2.28	1.27E-05	YES	
BG088381	RIKEN cDNA 2010100O12 gene	*2010100O12Rik*	1.45	2.74	9.89E-08	YES	unknown
AI327246	ESTs, Weakly similar to S20900 titin - mouse (fragment) [M.musculus]		-1.01	-2.02	2.03E-04	YES	
AI854139	ESTs, Moderately similar to T00388 hypothetical protein KIAA0616 - human (fragment) [H.sapiens]		1.43	2.69	1.75E-07	YES	

AI853288	ras homolog gene family, member U	*Arhu*	1.22	2.33	8.16E-06	YES	cell cycle
AI415047	cDNA sequence BC026432	*BC026432*	-1.09	-2.13	6.21E-05	YES	
BG088163	split hand/foot deleted gene 1	*Shfdg1*	1.62	3.08	2.78E-09	YES	development
AW558457			1.24	2.36	5.61E-06	YES	
BG075941	RIKEN cDNA 4833415E20 gene	*4833415E20Rik*	1.06	2.09	9.74E-05	YES	
AW558617			1.12	2.18	3.94E-05	YES	
AI851062	RIKEN cDNA 1300009F09 gene	*1300009F09Rik*	-1.21	-2.31	9.91E-06	YES	transport
AI414844	RIKEN cDNA 2310035L15 gene	*2310035L15Rik*	1.26	2.40	3.72E-06	YES	
BG075859	Nedd4 WW binding protein 4	*N4wbp4-pending*	-1.12	-2.18	3.83E-05	YES	unknown
BG075875	ESTs		-1.70	-3.26	4.32E-10	YES	
AI327242	RIKEN cDNA 1500032L24 gene	*1500032L24Rik*	-1.08	-2.12	7.37E-05	YES	unknown
BG075881	tyrosine 3-monooxygenase/tryptophan 5-monooxygenase activation protein, zeta polypeptide	*Ywhaz*	-1.29	-2.44	2.28E-06	YES	cell adhesion
BG088790	DNA segment, Chr 11, ERATO Doi 99, expressed	*D11Ertd99e*	1.01	2.02	2.02E-04	YES	unknown
AI853442	RIKEN cDNA 4930404J24 gene	*4930404J24Rik*	1.02	2.03	1.80E-04	YES	
BG076308			1.41	2.66	2.26E-07	YES	
AI325974	hypothetical protein MGC12070	*MGC12070*	1.39	2.61	3.86E-07	YES	
AW558832			1.03	2.04	1.68E-04	YES	
BG075691			1.17	2.26	1.69E-05	YES	
AW556069	hypothetical protein MGC37588	*MGC37588*	1.61	3.06	3.36E-09	YES	
AW557947			1.02	2.02	1.95E-04	YES	

BG067146			-1.24	-2.36	5.67E-06	YES	
AW559135			1.39	2.62	3.42E-07	YES	
AW557813			1.13	2.19	3.35E-05	YES	
AW557861			1.09	2.13	6.45E-05	YES	
BG088558	cDNA sequence BC003993	*BC003993*	1.16	2.24	2.12E-05	YES	
AW558971	ESTs		-1.00	-2.01	2.34E-04	YES	
AW556082	aladin	*LOC223921*	1.36	2.57	6.26E-07	YES	
AW556086			1.29	2.45	2.08E-06	YES	
BI076864			1.36	2.57	6.20E-07	YES	
AI414793	RIKEN cDNA 2010003K11 gene	*2010003K11Rik*	-1.01	-2.01	2.19E-04	YES	
AI413970	EST		1.11	2.16	4.66E-05	YES	
AI835656			1.35	2.56	6.98E-07	YES	
AI839988	hypothetical protein MGC38419	*MGC38419*	1.12	2.17	4.26E-05	YES	transport
AI838564	expressed sequence AL024016	*AL024016*	1.27	2.41	3.38E-06	YES	
AI838600	EST		1.36	2.56	6.79E-07	YES	
AI836953			1.19	2.28	1.34E-05	YES	
AI838366	bromodomain-containing 4	*Brd4*	1.06	2.09	1.01E-04	YES	unknown
AI836491	heat shock 10 kDa protein 1 (chaperonin 10)	*Hspe1*	1.58	2.99	7.11E-09	YES	chaperone
AI837036	Rab acceptor 1 (prenylated)	*Rabac1*	1.19	2.28	1.33E-05	YES	other
AI836010	RIKEN cDNA 1110013B16 gene	*1110013B16Rik*	1.30	2.46	1.90E-06	YES	unknown
AI841463	NK6 transcription factor related, locus 2 (Drosophila)	*Nkx6-2*	1.74	3.34	1.83E-10	YES	transcription regulator
AI842640	ESTs, Moderately similar to hypothetical protein FLJ11305 [Homo sapiens] [H.sapiens]		-1.28	-2.43	2.70E-06	YES	

AI846484	DNA segment, Chr 1, ERATO Doi 396, expressed	*D1Ertd396e*	1.03	2.04	1.66E-04	YES	
AI841315	EST		1.45	2.74	1.01E-07	YES	
AI843621	expressed sequence AI836659	*AI836659*	1.06	2.09	9.80E-05	YES	
AI839850	ESTs		1.09	2.14	6.07E-05	YES	
AI835325	kelch-like ECH-associated protein 1	*Keap1*	1.01	2.02	2.02E-04	YES	transcription regulator
AI836605	inhibitor of growth family, member 4	*Ing4*	1.22	2.32	8.21E-06	YES	transcription regulator
AI840241			1.18	2.27	1.41E-05	YES	
AI836925	ESTs, Moderately similar to G3P_MOUSE Glyceraldehyde 3-phosphate dehydrogenase (GAPDH) [M.musculus]		1.01	2.01	2.20E-04	YES	
AI839628			1.26	2.39	3.96E-06	YES	
AI845462	pyruvate dehydrogenase kinase, isoenzyme 3	*Pdk3*	-1.15	-2.22	2.39E-05	YES	other
AI845464	RIKEN cDNA 1700042O10 gene	*1700042O10Rik*	1.35	2.55	7.46E-07	YES	
AI837600	expressed sequence AI850305	*AI850305*	1.05	2.07	1.18E-04	YES	
AI845502	eukaryotic translation initiation factor 3	*Eif3*	1.50	2.83	4.00E-08	YES	protein synthesis
AI837602	EST		1.08	2.12	7.34E-05	YES	
AI845510	ESTs		1.32	2.49	1.38E-06	YES	
AI837644	RIKEN cDNA 2900002K07 gene	*2900002K07Rik*	1.74	3.34	1.90E-10	YES	unknown
AI846726	EST		1.43	2.70	1.48E-07	YES	
AI842597	transcription factor 4	*Tcf4*	1.39	2.63	3.25E-07	YES	development
AI839330	neuron specific gene family member 1	*Nsg1*	1.22	2.32	8.32E-06	YES	neurogenesis
AI839375	ESTs		1.46	2.75	8.87E-08	YES	

AI839382	lysosomal apyrase-like 1	*Lysal1*	1.55	2.92	1.46E-08	YES	other
AI841241	galactosidase, beta 1	*Glb1*	1.16	2.24	2.07E-05	YES	other
AI836097	EST		1.38	2.61	4.04E-07	YES	
AI839010			1.32	2.49	1.40E-06	YES	
AI837859	RIKEN cDNA 1810036J22 gene	*1810036J22Rik*	1.82	3.53	2.70E-11	YES	
AI841880	endothelial cell-selective adhesion molecule	*Esam-pending*	1.19	2.29	1.23E-05	YES	cell adhesion
AI841883			1.79	3.45	5.81E-11	YES	
AI837370	EST, Weakly similar to JW0059 mtprd protein - mouse [M.musculus]		1.76	3.38	1.20E-10	YES	
AI835930			-1.63	-3.09	2.33E-09	YES	
AI839548			1.80	3.48	4.34E-11	YES	
AI837434	RIKEN cDNA 4933428G20 gene	*4933428G20Rik*	1.03	2.05	1.54E-04	YES	
AI838946	Mus musculus, clone IMAGE:4527484, mRNA, partial cds		1.26	2.39	4.24E-06	YES	
AI845665			1.89	3.70	4.60E-12	YES	
AI840835	EST		1.50	2.83	3.92E-08	YES	
AI836199	protein kinase, cAMP dependent regulatory, type I, alpha	*Prkar1a*	1.01	2.02	2.12E-04	YES	development
AI839716	RIKEN cDNA 4931420H10 gene	*4931420H10Rik*	1.48	2.78	6.22E-08	YES	
AI836541	ribosomal protein L19	*Rpl19*	1.54	2.91	1.58E-08	YES	protein synthesis
AI835847	ESTs, Highly similar to NUMM_MOUSE NADH-ubiquinone oxidoreductase 13 kDa-A subunit (Complex I-13KD-A) (CI-13KD-A) [M.musculus]		1.57	2.97	8.52E-09	YES	
AI839755	insulin receptor substrate 1	*Irs1*	1.12	2.18	3.94E-05	YES	signal transducer

AI835645	formin binding protein 4	*Fnbp4*	1.13	2.19	3.35E-05	YES	
AI842497	RIKEN cDNA 1810060J02 gene	*1810060J02Rik*	1.54	2.91	1.58E-08	YES	
AI845440	RIKEN cDNA C330007D15 gene	*C330007D15Rik*	1.11	2.16	4.87E-05	YES	
AI843756	calmodulin 2	*Calm2*	-1.11	-2.16	4.79E-05	YES	cell cycle
AI836098	EST		1.37	2.58	5.58E-07	YES	
AI840311	EST		1.58	2.99	7.36E-09	YES	
AI841876	hemoglobin, beta adult minor chain	*Hbb-b2*	1.50	2.82	4.28E-08	YES	transport
AI837996	GRP1 (general receptor for phosphoinositides 1)-associated scaffold protein	*Grasp*	1.08	2.11	7.91E-05	YES	signal transducer
AI841881	RIKEN cDNA 2410008M22 gene	*2410008M22Rik*	1.12	2.17	4.15E-05	YES	
AI835909	ribosomal protein S3	*Rps3*	1.22	2.32	8.29E-06	YES	protein synthesis
AI837371	RIKEN cDNA 1810005K14 gene	*1810005K14Rik*	1.17	2.25	1.85E-05	YES	
AI837380	hypothetical protein MGC36388	*MGC36388*	1.35	2.55	7.76E-07	YES	mitochondria
AI838940	RIKEN cDNA 1110057L18 gene	*1110057L18Rik*	1.42	2.67	2.04E-07	YES	
AI840337			1.04	2.05	1.41E-04	YES	
AI841536			1.46	2.76	8.01E-08	YES	
AI845861	syntaxin 6	*Stx6*	1.01	2.02	2.02E-04	YES	transport
AI835607			1.03	2.04	1.59E-04	YES	
AI840347	EST		1.33	2.52	1.03E-06	YES	
AI839804	CDC-like kinase 2	*Clk2*	1.06	2.08	1.05E-04	YES	other
AI839739	EST, Weakly similar to TBA1_MOUSE Tubulin alpha-1 chain [M.musculus]		1.20	2.30	1.08E-05	YES	

AI835779	EST, Weakly similar to BASI_MOUSE Basigin precursor (Basic immunoglobulin superfamily) (Membrane glycoprotein GP42) [M.musculus]		1.09	2.12	6.90E-05	YES	
AI839797	RIKEN cDNA 1700008C22 gene	*1700008C22Rik*	1.10	2.14	5.69E-05	YES	
AI835639	ESTs		1.28	2.42	2.83E-06	YES	
AI843597	RIKEN cDNA 4921519L13 gene	*4921519L13Rik*	1.02	2.03	1.80E-04	YES	
AI837129	ESTs		1.39	2.63	3.24E-07	YES	
AI843136	N-myc downstream regulated 2	*Ndr2*	-1.15	-2.23	2.34E-05	YES	development
AI845449	cDNA sequence BC020156	*BC020156*	-1.32	-2.49	1.36E-06	YES	
AI838353	ESTs, Weakly similar to MAPB_MOUSE Microtubule-associated protein 1B (MAP 1B) (MAP1.2) (MAP1(X)) [Contains: MAP1 light chain LC1] [M.musculus]		1.02	2.02	1.99E-04	YES	
AI836589	ATP synthase, H+ transporting mitochondrial F1 complex, beta subunit	*Atp5b*	1.30	2.46	1.98E-06	YES	transport
AI835315	Mus musculus, clone IMAGE:3586350, mRNA, partial cds		1.46	2.75	9.28E-08	YES	
AI839857	similar to hypothetical protein FLJ12949	*LOC215194*	1.15	2.22	2.46E-05	YES	
AI836940	EST		1.17	2.25	1.76E-05	YES	

AI838397	EST, Weakly similar to COP9 subunit 6 (MOV34 homolog, 34 kD) [Homo sapiens] [H.sapiens]		1.60	3.03	4.40E-09	YES	
AI835378	ESTs		1.13	2.18	3.64E-05	YES	
AI837335	EST		1.94	3.85	1.03E-12	YES	
AI845521	myosin VIIA and Rab interacting protein	*Myrip*	1.22	2.33	7.99E-06	YES	transport
AI834863	ribosomal protein L26	*Rpl26*	1.31	2.48	1.53E-06	YES	protein synthesis
AI839202	EST		1.05	2.07	1.21E-04	YES	
AI839265	EST		1.27	2.40	3.50E-06	YES	
AI836361	G-protein coupled receptor 88	*Gpr88*	1.04	2.05	1.41E-04	YES	signal transducer
AI835993	EST		1.24	2.37	5.31E-06	YES	
AI841812	expressed sequence AI043088	*AI043088*	1.58	3.00	6.60E-09	YES	
AI842966	RIKEN cDNA 1110019I12 gene	*1110019I12Rik*	1.54	2.91	1.60E-08	YES	
AI840930	ESTs		1.56	2.95	1.11E-08	YES	
AI846549	RIKEN cDNA 2610019F03 gene	*2610019F03Rik*	-1.07	-2.10	8.54E-05	YES	
AI843570	ESTs		1.35	2.54	8.08E-07	YES	
AI839125	RIKEN cDNA 3100001N19 gene	*3100001N19Rik*	1.08	2.12	7.17E-05	YES	protein synthesis
AI839134	eukaryotic translation initiation factor 3, subunit 3 (gamma, 40kD)	*Eif3s3*	1.09	2.13	6.29E-05	YES	protein synthesis
AI840275	EST, Moderately similar to 0806162D protein COII [Mus musculus] [M.musculus]		1.31	2.47	1.67E-06	YES	
AI839634			1.42	2.67	2.11E-07	YES	
AI840425	EST		1.24	2.37	5.10E-06	YES	
AI836708			-1.22	-2.32	8.41E-06	YES	

AI845753	RIKEN cDNA 2810422J05 gene	*2810422J05Rik*	1.46	2.74	9.40E-08	YES	
AI843781	protein phosphatase 3, catalytic subunit, alpha isoform	*Ppp3ca*	1.32	2.50	1.22E-06	YES	
AI845485	four and a half LIM domains 4	*Fhl4*	1.05	2.07	1.18E-04	YES	transport
AI845532	Mus musculus, Similar to SEC24 related gene family, member C (S. cerevisiae), clone IMAGE:4950601, mRNA, partial cds		1.24	2.37	5.17E-06	YES	other
AI846743	transgelin	*Tagln*	1.41	2.65	2.48E-07	YES	development
AI846795	EST		1.37	2.58	5.58E-07	YES	
AI837029	reticulocalbin	*Rcn*	1.05	2.07	1.19E-04	YES	
AI841447			1.38	2.60	4.34E-07	YES	
AI841802	seryl-aminoacyl-tRNA synthetase 1	*Sars1*	1.04	2.05	1.47E-04	YES	other
BG086264	Polymerase (RNA) II, DNA directed	*Polr2*	-1.71	-3.27	5.76E-06	YES	RNA synthesis
AI843553	heat shock 70kD protein 5 (glucose-regulated protein, 78kD)	*Hspa5*	1.15	2.21	2.70E-05	YES	heat shock response
AI842099	protein kinase C, alpha	*Prkca*	1.05	2.08	1.14E-04	YES	signal transducer
AI842759	glycine C-acetyltransferase (2-amino-3-ketobutyrate-coenzyme A ligase)	*Gcat*	-1.09	-2.13	6.43E-05	YES	mitochondria
AI849019	myelin-associated oligodendrocytic basic protein	*Mobp*	-1.32	-2.50	1.26E-06	YES	
AI847890	proteolipid protein (myelin)	*Plp*	-1.11	-2.16	4.72E-05	YES	neuronal function
AI848720			-1.34	-2.52	9.82E-07	YES	
AI849605	ESTs		-1.18	-2.26	1.63E-05	YES	

AI853494	expressed sequence AI853494	*AI853494*	-1.06	-2.08	1.08E-04	YES	cell adhesion
AI834974	cDNA sequence BC002292	*BC002292*	1.37	2.59	4.82E-07	YES	
AI849487	EST		1.69	3.22	6.38E-10	YES	
AI849816	RIKEN cDNA 2310066E14 gene	*2310066E14Rik*	-1.05	-2.07	1.19E-04	YES	
AI850771	EST		1.05	2.07	1.18E-04	YES	
AI853558	tumor necrosis factor receptor superfamily, member 12a	*Tnfrsf12a*	-1.08	-2.11	7.54E-05	YES	apoptosis control
AI849240	RIKEN cDNA 2900002L20 gene	*2900002L20Rik*	-1.35	-2.55	7.78E-07	YES	
AI848921	ESTs		-1.51	-2.84	3.40E-08	YES	
AI844003	RIKEN cDNA 4930566A11 gene	*4930566A11Rik*	1.16	2.23	2.18E-05	YES	
AI844250	RIKEN cDNA 1210002E11 gene	*1210002E11Rik*	1.02	2.02	1.94E-04	YES	
AI849086	contactin 1	*Cntn1*	-1.15	-2.22	2.39E-05	YES	cell adhesion
AI850252	RIKEN cDNA 6720463E02 gene	*6720463E02Rik*	1.01	2.02	2.04E-04	YES	other
AI844955			1.53	2.88	2.18E-08	YES	
AI850305	expressed sequence AI850305	*AI850305*	1.13	2.19	3.55E-05	YES	
AI848307	staufen (RNA binding protein) homolog 2 (Drosophila)	*Stau2*	-1.10	-2.14	5.50E-05	YES	
AI848669	expressed sequence AI848669	*AI848669*	-1.04	-2.06	1.37E-04	YES	
AI850662	EST		-1.13	-2.19	3.58E-05	YES	
AI853761	proline rich protein expressed in brain	*Prtb*	-1.14	-2.21	2.81E-05	YES	
AI850716	ESTs, Weakly similar to I58401 protein-tyrosine kinase (EC 2.7.1.112) JAK3 - mouse [M.musculus]		-1.09	-2.12	6.85E-05	YES	

AI849100	DNA segment, Chr 6, Wayne State University 116, expressed	*D6Wsu116e*	-1.16	-2.23	2.19E-05	YES	
AI854434	ribosomal protein L13a	*Rpl13a*	1.13	2.19	3.47E-05	YES	development
AI852095	RIKEN cDNA 5730536A07 gene	*5730536A07Rik*	1.04	2.06	1.35E-04	YES	
AI850277	neuromedin	*Nmu*	1.02	2.03	1.84E-04	YES	neuronal function
AI842821	phospholipase C-like 2	*Plcl2*	1.08	2.12	7.19E-05	YES	signal transducer
AI849546	ESTs		1.12	2.17	4.02E-05	YES	
AI852498	EST		1.03	2.04	1.67E-04	YES	
AI845098	tRNA nucleotidyl transferase, CCA-adding, 1	*Trnt1*	1.19	2.29	1.24E-05	YES	mitochondria
AI850099	RIKEN cDNA 2900002L20 gene	*2900002L20Rik*	1.07	2.11	8.24E-05	YES	unknown
AI842775	RIKEN cDNA 2210008F15 gene	*2210008F15Rik*	-1.12	-2.18	3.92E-05	YES	
AI848163	ESTs		1.74	3.34	1.80E-10	YES	
AI842783	RIKEN cDNA 2210403N23 gene	*2210403N23Rik*	-1.17	-2.24	1.96E-05	YES	unknown
AI842820	RIKEN cDNA 5730456K23 gene	*5730456K23Rik*	-1.09	-2.13	6.63E-05	YES	
AI842822	RIKEN cDNA 1110003F06 gene	*1110003F06Rik*	1.20	2.30	1.08E-05	YES	
AI846978	leucine rich repeat (in FLII) interacting protein 2	*Lrrfip2*	-1.39	-2.62	3.58E-07	YES	contains DNA repair motif
AI847514	solute carrier family 1, member 3	*Slc1a3*	-1.12	-2.17	4.34E-05	YES	transport
AI847007	NCK-associated protein 1	*Nckap1*	-1.01	-2.02	2.06E-04	YES	apoptosis control
AI847015	RIKEN cDNA 0710001P18 gene	*0710001P18Rik*	-1.01	-2.02	2.05E-04	YES	
AI847521	ESTs		-1.06	-2.09	9.89E-05	YES	
AI849136	EST		1.12	2.17	4.12E-05	YES	
AI844569	RIKEN cDNA 1100001J08 gene	*1100001J08Rik*	1.07	2.09	9.34E-05	YES	

AI851981	growth hormone inducible transmembrane protein	*Ghitm*	-1.37	-2.58	5.25E-07	YES	unknown
AI850599	ESTs, Moderately similar to transcriptional regulating protein 132, isoform 2; rapa-2; rapa-1 [Homo sapiens] [H.sapiens]		1.17	2.25	1.80E-05	YES	
AI847692	EST		1.09	2.13	6.67E-05	YES	
AI842319	RIKEN cDNA 1300014E15 gene	*1300014E15Rik*	1.04	2.06	1.39E-04	YES	
AI849361	RIKEN cDNA 1700056O17 gene	*1700056O17Rik*	1.29	2.44	2.40E-06	YES	
AI847090	serine protease inhibitor, Kunitz type 2	*Spint2*	1.17	2.25	1.74E-05	YES	other
AI853386	EST		2.26	4.78	2.22E-16	YES	
AI850680	Mus musculus adult male medulla oblongata cDNA, RIKEN full-length enriched library, clone:6330407D06:ornithine decarboxylase antizyme 3, full insert sequence		1.22	2.32	8.40E-06	YES	other
AI851852	ESTs		1.34	2.53	9.48E-07	YES	
AI852958	ESTs, Weakly similar to pregnancy upregulated non-ubiquitously expressed CaM kinase; calcium/calmodulin-dependent protein kinase 1, beta [Mus musculus] [M.musculus]		1.34	2.53	8.89E-07	YES	

AI848056	RIKEN cDNA 2700079K05 gene	*2700079K05Rik*	1.25	2.38	4.46E-06	YES	
AI847909	RIKEN cDNA 1700030A21 gene	*1700030A21Rik*	-1.22	-2.33	7.43E-06	YES	
AI848830	isocitrate dehydrogenase 2 (NADP+), mitochondrial	*Idh2*	-1.13	-2.19	3.46E-05	YES	mitochondria
AI844129	cDNA sequence BC017158	*BC017158*	1.56	2.95	1.11E-08	YES	
AI853788	ESTs		-1.33	-2.52	1.08E-06	YES	
AI854455	ESTs, Weakly similar to A41234 melanocyte-specific protein Pmel-17 precursor - human [H.sapiens]		-1.02	-2.03	1.81E-04	YES	
AI852742	RIKEN cDNA B430305P08 gene	*B430305P08Rik*	-1.04	-2.06	1.39E-04	YES	
AI851386	EST		-1.27	-2.40	3.53E-06	YES	
AI851519	RIKEN cDNA 6430411K14 gene	*6430411K14Rik*	-1.03	-2.04	1.57E-04	YES	
AI851532	ESTs		-1.03	-2.04	1.65E-04	YES	
BG076127	expressed sequence AU021063	*AU021063*	1.34	2.52	9.79E-07	YES	
AW556296	MLN51 protein	*Mln51-pending*	1.16	2.24	2.01E-05	YES	
BG088348	cDNA sequence BC032200	*BC032200*	1.33	2.52	1.00E-06	YES	
BG075824	ESTs, Weakly similar to S24407 formin isoform IV - mouse [M.musculus]		-1.04	-2.05	1.46E-04	YES	
BG075826	RIKEN cDNA 5430400P17 gene	*5430400P17Rik*	1.24	2.36	5.54E-06	YES	
AW556391	ribosomal protein L32	*Rpl32*	1.39	2.62	3.66E-07	YES	protein synthesis
BG075844	ESTs		-1.18	-2.26	1.54E-05	YES	
AW557368			1.10	2.14	5.48E-05	YES	

BG088092	solute carrier family 14 (urea transprorter), member 1	*Slc14a1*	-1.07	-2.10	8.57E-05	YES	transport
BG088094	succinate-Coenzyme A ligase, GDP-forming, beta subunit	*Suclg2*	-1.23	-2.35	6.11E-06	YES	mitochondria
BG076199	ESTs		1.40	2.63	3.15E-07	YES	
BG076200	DAZ associated protein 1	*Dazap1*	1.13	2.19	3.53E-05	YES	mitochondria
BG075908	epithelial protein lost in neoplasm	*Eplin-pending*	1.05	2.08	1.13E-04	YES	unknown
BG076279	ESTs		1.27	2.42	3.02E-06	YES	
AI854856	ESTs		-1.13	-2.20	3.21E-05	YES	
AI323874	EST		1.06	2.08	1.05E-04	YES	
AI852836	RIKEN cDNA 5530600M07 gene	*5530600M07Rik*	1.08	2.12	7.11E-05	YES	
BG067231	ESTs, Weakly similar to RIKEN cDNA 5730493B19 [Mus musculus] [M.musculus]		-1.07	-2.09	9.27E-05	YES	
BG088708	nuclear factor, erythroid derived 2,-like 1	*Nfe2l1*	1.42	2.68	1.88E-07	YES	transcription regulator
BG088727	villin 2	*Vil2*	1.03	2.04	1.58E-04	YES	cytoskeleton
BG088764	RIKEN cDNA 1810024K12 gene	*1810024K12Rik*	1.14	2.20	3.12E-05	YES	unknown
BG085369			1.01	2.02	3.35E-04	YES	
BG071585			1.00	2.01	3.69E-04	YES	
AW550291			-1.83	-3.56	8.11E-11	YES	
BG073921	ESTs, Weakly similar to cofactor required for Sp1 transcriptional activation subunit 2 (150 kDa) [Mus musculus] [M.musculus]		-1.01	-2.02	3.30E-04	YES	
BG072622	ESTs		1.18	2.27	2.64E-05	YES	

BG073321			1.32	2.50	2.76E-06	YES	
BI076595			1.37	2.59	1.11E-06	YES	
BI076595			1.61	3.05	1.18E-08	YES	
BI076595			1.36	2.56	1.47E-06	YES	
BG084405	baculoviral IAP repeat-containing 2	*Birc2*	1.29	2.45	4.45E-06	YES	apoptosis control
BG072917	ESTs		1.05	2.07	2.04E-04	YES	
BG085378	myristoylated alanine rich protein kinase C substrate	*Marcks*	1.29	2.45	4.51E-06	YES	
AU045439	EST, Weakly similar to S12207 hypothetical protein (B2 element) - mouse [M.musculus]		1.12	2.18	6.85E-05	YES	
BG086908			2.22	4.65	3.77E-15	YES	
BG075200			1.63	3.10	6.76E-09	YES	
BG072323	ESTs, Moderately similar to S12207 hypothetical protein (B2 element) - mouse [M.musculus]		1.24	2.37	1.04E-05	YES	
BG072325	ESTs, Weakly similar to RIKEN cDNA 5730493B19 [Mus musculus] [M.musculus]		1.08	2.11	1.36E-04	YES	
BG071669	ESTs, Weakly similar to RIKEN cDNA 5730493B19 [Mus musculus] [M.musculus]		1.17	2.25	3.49E-05	YES	
BG072375	ESTs, Weakly similar to RIKEN cDNA 5730493B19 [Mus musculus] [M.musculus]		1.25	2.38	8.92E-06	YES	

BG073944	ESTs, Weakly similar to I58401 protein-tyrosine kinase (EC 2.7.1.112) JAK3 - mouse [M.musculus]		1.86	3.62	4.55E-11	YES	
BG074985	ESTs		1.01	2.01	3.64E-04	YES	
BG064262	ESTs		-1.07	-2.10	1.51E-04	YES	
BG066020	DNA segment, Chr 5, ERATO Doi 66, expressed	*D5Ertd66e*	-1.08	-2.11	1.37E-04	YES	
BG065423	ESTs		-1.54	-2.90	5.15E-08	YES	
BG063324	DNA segment, Chr 11, Wayne State University 173, expressed	*D11Wsu173e*	-1.09	-2.13	1.14E-04	YES	
C77806			1.08	2.11	1.28E-04	YES	
BG066813	expressed sequence C80446	*C80446*	1.28	2.43	5.35E-06	YES	
BG084605	2,3-bisphosphoglycerate mutase	*Bpgm*	-1.02	-2.02	3.14E-04	YES	other
BG073394	adducin 3 (gamma)	*Add3*	-1.23	-2.35	1.22E-05	YES	cytoskeleton
BG073422	nuclear distribution gene C homolog (Aspergillus)	*Nudc*	-1.09	-2.13	1.07E-04	YES	unknown
BG073663	ESTs, Weakly similar to S12207 hypothetical protein (B2 element) - mouse [M.musculus]		1.05	2.07	2.01E-04	YES	
AW555131	EST, Weakly similar to RL15_MOUSE 60S ribosomal protein L15 [M.musculus]		1.19	2.28	2.42E-05	YES	
BG075043			1.11	2.16	8.05E-05	YES	
BG074735	ESTs, Weakly similar to S26689 hypothetical protein hc1 - mouse (fragment) [M.musculus]		1.80	3.49	1.63E-10	YES	

BG074418	hypothetical protein MGC11659	*MGC11659*	1.29	2.45	4.76E-06	YES	transcription regulator
BG074421	ESTs		1.20	2.30	1.96E-05	YES	
BG074423	RIKEN cDNA 1700057K18 gene	*1700057K18Rik*	-2.46	-5.50	0.00E+00	YES	RNA processing
BG072130	ESTs		1.14	2.20	5.55E-05	YES	
BG073135	ESTs		1.74	3.33	7.55E-10	YES	
BG071454			1.09	2.13	1.09E-04	YES	
BG071830	ESTs, Weakly similar to RIKEN cDNA 5730493B19 [Mus musculus] [M.musculus]		1.17	2.25	3.23E-05	YES	
BG087422			1.50	2.82	1.14E-07	YES	
BG072400	ESTs, Weakly similar to RIKEN cDNA 5730493B19 [Mus musculus] [M.musculus]		1.07	2.10	1.52E-04	YES	
BG071400	ESTs, Weakly similar to RIKEN cDNA 5730493B19 [Mus musculus] [M.musculus]		1.26	2.39	8.18E-06	YES	
AU041361			1.28	2.42	5.89E-06	YES	
BG072740	actin related protein 2/3 complex, subunit 3 (21 kDa)	*Arpc3*	1.19	2.29	2.35E-05	YES	cytoskeleton
BG072449	ESTs		1.01	2.01	3.46E-04	YES	
BG072763	expressed sequence AI120128	*AI120128*	1.01	2.01	3.43E-04	YES	
BG073099			1.22	2.32	1.64E-05	YES	
BG072469			1.32	2.50	2.85E-06	YES	
BG072115			1.19	2.28	2.55E-05	YES	
BG086759			1.23	2.34	1.36E-05	YES	
BG086772	RIKEN cDNA 1810060K07 gene	*1810060K07Rik*	1.48	2.79	1.53E-07	YES	

BG074063	cleavage stimulation factor, 3' pre-RNA subunit 2, 64 kDa	*Cstf2*	1.51	2.84	8.88E-08	YES	
BG073438	RIKEN cDNA 0610025G13 gene	*0610025G13Rik*	1.04	2.06	2.22E-04	YES	protein synthesis
BG086475	ribosomal protein S29	*Rps29*	1.08	2.11	1.30E-04	YES	protein synthesis
BG074079	DNA segment, Chr 7, ERATO Doi 152, expressed	*D7Ertd152e*	1.30	2.45	4.33E-06	YES	transport
BG074083	ESTs		1.46	2.76	2.06E-07	YES	
BG086812	RIKEN cDNA 6720407C15 gene	*6720407C15Rik*	1.35	2.55	1.73E-06	YES	signal transducer
BG086818	ribosomal protein S14	*Rps14*	1.58	3.00	1.94E-08	YES	protein synthesis
BG071452			1.03	2.04	2.56E-04	YES	
BG071795	ESTs, Weakly similar to nuclear localization signals binding protein 1 [Mus musculus] [M.musculus]		1.03	2.05	2.48E-04	YES	
AU042949	ESTs, Weakly similar to RIKEN cDNA 5730493B19 [Mus musculus] [M.musculus]		1.09	2.13	1.08E-04	YES	
BG071808	ESTs, Moderately similar to S12207 hypothetical protein (B2 element) - mouse [M.musculus]		1.06	2.08	1.73E-04	YES	
BG072159	ESTs		1.36	2.56	1.50E-06	YES	
AU042956			1.05	2.08	1.83E-04	YES	
AU041764	RIKEN cDNA 2510001K09 gene	*2510001K09Rik*	1.08	2.12	1.19E-04	YES	
BG073788	ESTs		1.10	2.14	1.02E-04	YES	
BG074143	RIKEN cDNA 2010003I19 gene	*2010003I19Rik*	1.04	2.05	2.38E-04	YES	
BG073855			1.42	2.68	4.38E-07	YES	

BG085056	signal recognition particle 9 kDa	*Srp9*	1.52	2.87	6.86E-08	YES	other
BG085958	ribosomal protein S26	*Rps26*	1.16	2.24	3.86E-05	YES	protein synthesis
BG072887	ESTs		1.30	2.46	4.01E-06	YES	
BG073225	ESTs		1.20	2.29	2.22E-05	YES	
BG072567	ESTs		1.27	2.41	6.45E-06	YES	
BG085978	mitogen activated protein kinase kinase kinase 3	*Map3k3*	1.37	2.59	1.17E-06	YES	signal transducer
BG072571	phosphatidylinositol 4-kinase type 2 alpha	*Pi4k2a-pending*	1.22	2.33	1.47E-05	YES	unknown
BG072912	cytochrome c oxidase, subunit VIIa 2	*Cox7a2*	1.58	2.98	2.29E-08	YES	mitochondria
BG072585	ESTs		-1.03	-2.05	2.51E-04	YES	
BG085383	RIKEN cDNA 2310036I02 gene	*2310036I02Rik*	1.33	2.51	2.53E-06	YES	mitochondria
BG085385	hypothetical protein MGC37079	*MGC37079*	-1.13	-2.19	6.25E-05	YES	
BG072935	RIKEN cDNA 0610039D01 gene	*0610039D01Rik*	1.06	2.09	1.62E-04	YES	
BG085732	ESTs		1.11	2.16	7.90E-05	YES	
AW550294			-1.27	-2.41	6.57E-06	YES	
AW550368			-1.25	-2.37	9.94E-06	YES	
BG086313	guanine nucleotide binding protein, alpha 12	*Gna12*	-1.44	-2.71	3.23E-07	YES	signal transducer
BG086961	methionine aminopeptidase 2	*Metap2*	-1.22	-2.33	1.53E-05	YES	other
AW552752	RIKEN cDNA 1110030M18 gene	*1110030M18Rik*	-1.08	-2.11	1.32E-04	YES	
BG073336	ESTs, Moderately similar to S12207 hypothetical protein (B2 element) - mouse [M.musculus]		1.14	2.21	4.93E-05	YES	
BG074626			1.18	2.26	3.07E-05	YES	

BG071871	ESTs, Weakly similar to RIKEN cDNA 5730493B19 [Mus musculus] [M.musculus]		1.20	2.30	2.01E-05	YES	
BG071873	ESTs, Weakly similar to RIKEN cDNA 5730493B19 [Mus musculus] [M.musculus]		1.10	2.14	9.58E-05	YES	
BG072219	ESTs		1.37	2.59	1.14E-06	YES	
BG085341	ribosomal protein S12	*Rps12*	1.48	2.79	1.51E-07	YES	protein synthesis
BG071878	ESTs		1.38	2.61	9.60E-07	YES	
BG072244	calmodulin 1	*Calm1*	1.09	2.13	1.12E-04	YES	cell cycle
BG071557	metastasis associated 3	*Mta3*	1.11	2.16	8.27E-05	YES	unknown
BG085363	proteasome (prosome, macropain) 26S subunit, non-ATPase, 11	*Psmd11*	1.44	2.71	3.50E-07	YES	proteasome
BG072586	ESTs		1.41	2.66	5.43E-07	YES	
BG072934	ESTs, Weakly similar to I58401 protein-tyrosine kinase (EC 2.7.1.112) JAK3 - mouse [M.musculus]		1.55	2.92	4.00E-08	YES	
AW551791			-1.25	-2.37	1.01E-05	YES	
BG074900			1.60	3.04	1.28E-08	YES	
BG072366	RIKEN cDNA 1700013B03 gene	*1700013B03Rik*	1.04	2.06	2.25E-04	YES	
BG065238	Mus musculus, Similar to hypothetical protein FLJ22479, clone IMAGE:4487274, mRNA, partial cds		-1.11	-2.16	8.41E-05	YES	

BG064891	eukaryotic translation initiation factor 3, subunit 6 interacting protein	*Eif3s6ip*	1.37	2.58	1.19E-06	YES	protein synthesis
BG076874	expressed sequence AA959601	*AA959601*	1.04	2.05	2.29E-04	YES	
BG078797	Mus musculus, Similar to hypothetical protein FLJ20718, clone IMAGE:5372218, mRNA, partial cds		1.23	2.34	1.37E-05	YES	
AW537827	transforming growth factor beta 1 induced transcript 4	*Tgfb1i4*	1.18	2.27	2.64E-05	YES	transcription regulator
BG078816	3-hydroxy-3-methylglutaryl-Coenzyme A reductase	*Hmgcr*	1.37	2.59	1.13E-06	YES	
BG065911			1.15	2.22	4.61E-05	YES	
BG076914	adenylosuccinate lyase	*Adsl*	1.02	2.03	3.03E-04	YES	other
BG063238	RIKEN cDNA 2310002J21 gene	*2310002J21Rik*	1.05	2.07	2.03E-04	YES	apoptosis control
BG066276			1.17	2.25	3.25E-05	YES	
BG066591			1.28	2.42	6.02E-06	YES	
BG066166	DNA segment, Chr 17, ERATO Doi 165, expressed	*D17Ertd165e*	1.12	2.17	7.06E-05	YES	
BG066789	expressed sequence AI317193	*AI317193*	1.09	2.13	1.12E-04	YES	
BG064721	RIKEN cDNA 2810482I07 gene	*2810482I07Rik*	1.11	2.16	8.16E-05	YES	transport
BG063365	chemokine (C-X-C motif) receptor 4	*Cxcr4*	1.26	2.39	8.13E-06	YES	development

BG062998	ESTs, Weakly similar to PMXB_MOUSE PAIRED MESODERM HOMEOBOX PROTEIN 2B (PAIRED-LIKE HOMEOBOX 2B) (PHOX2B HOMEODOMAIN PROTEIN) (NEUROBLASTOMA PHOX) (NBPHOX) [M.musculus]	1.08	2.12	1.27E-04	YES	
BG066122		1.07	2.09	1.55E-04	YES	
BG065847	ESTs, Weakly similar to RIKEN cDNA 5730493B19 [Mus musculus] [M.musculus]	1.16	2.23	4.06E-05	YES	
BG066523		1.26	2.39	8.29E-06	YES	
AA408425	ESTs	-1.00	-2.00	3.76E-04	YES	
BG064514		1.22	2.33	1.49E-05	YES	
BG064947	ESTs, Highly similar to S12207 hypothetical protein (B2 element) - mouse [M.musculus]	1.26	2.39	8.01E-06	YES	
BG065622		1.37	2.59	1.10E-06	YES	
BG066581	ESTs, Weakly similar to S12207 hypothetical protein (B2 element) - mouse [M.musculus]	1.46	2.75	2.19E-07	YES	
BG066583	ESTs, Highly similar to hypothetical brain protein similar to X96994 BR-1 protein (Helix pomatia) [Mus musculus] [M.musculus]	1.42	2.68	4.72E-07	YES	

C79973	DNA segment, Chr 8, ERATO Doi 294, expressed	*D8Ertd294e*	1.36	2.56	1.46E-06	YES	
BG076878	lipoprotein lipase	*Lpl*	1.02	2.03	2.87E-04	YES	transport
BG063860			1.10	2.14	9.85E-05	YES	
BG063478	peroxiredoxin 1	*Prdx1*	1.00	2.00	3.85E-04	YES	other
BG080034	transient receptor potential cation channel, subfamily M, member 1	*Trpm1*	1.00	2.00	3.78E-04	YES	transport
BG065928	hepatoma up-regulated protein	*Hurp-pending*	1.08	2.11	1.27E-04	YES	
BI076530	ESTs		1.15	2.22	4.53E-05	YES	
BG064517	Wiskott-Aldrich syndrome homolog (human)	*Was*	1.13	2.19	6.06E-05	YES	
BG077934	ESTs		1.11	2.16	8.22E-05	YES	
BG066978	ESTs		1.40	2.63	7.38E-07	YES	
BG066560	ESTs		1.79	3.47	1.99E-10	YES	
BG066562	proteasome (prosome, macropain) 26S subunit, non-ATPase, 7	*Psmd7*	1.95	3.86	4.88E-12	YES	proteasome
BG066991	expressed sequence C81269	*C81269*	1.47	2.77	1.92E-07	YES	
BG065627	ESTs		1.14	2.21	4.99E-05	YES	
C79876	ESTs		1.00	2.01	3.65E-04	YES	
BG066997	expressed sequence C81285	*C81285*	1.10	2.15	9.10E-05	YES	
BG066999	expressed sequence C81296	*C81296*	1.38	2.60	1.05E-06	YES	
BG065645	DNA segment, Chr 16, ERATO Doi 6, expressed	*D16Ertd6e*	1.42	2.68	4.44E-07	YES	
BG079828	Mus musculus, Similar to hypothetical protein FLJ20509, clone IMAGE:3489119, mRNA, partial cds		1.17	2.25	3.43E-05	YES	

BG066582	ESTs, Highly similar to S12207 hypothetical protein (B2 element) - mouse [M.musculus]		1.17	2.25	3.22E-05	YES	
BG067014	carbonyl reductase 3	*Cbr3*	1.44	2.71	3.36E-07	YES	other
BG065653	ESTs		1.15	2.22	4.61E-05	YES	
BG066328	expressed sequence C78977	*C78977*	1.63	3.10	6.91E-09	YES	
BG066330	ESTs		1.08	2.11	1.30E-04	YES	
BG079273	RIKEN cDNA 1110018O12 gene	*1110018O12Rik*	1.27	2.41	6.86E-06	YES	
BG066009	expressed sequence C77691	*C77691*	1.42	2.68	4.61E-07	YES	
BG066010	expressed sequence C77681	*C77681*	1.19	2.28	2.42E-05	YES	
BG065676	RIKEN cDNA 6230410L23 gene	*6230410L23Rik*	1.03	2.04	2.71E-04	YES	
BG067442			1.38	2.59	1.08E-06	YES	
BG084049	ESTs		1.07	2.09	1.57E-04	YES	
BG083644	P450 (cytochrome) oxidoreductase	*Por*	1.07	2.10	1.41E-04	YES	other
C85250			1.21	2.31	1.87E-05	YES	
BG067162	ESTs, Weakly similar to RIKEN cDNA 5730493B19 [Mus musculus] [M.musculus]		1.54	2.92	4.32E-08	YES	
BG068598	ESTs, Weakly similar to RIKEN cDNA 5730493B19 [Mus musculus] [M.musculus]		1.28	2.43	5.33E-06	YES	
BG071170	ESTs		1.02	2.03	3.02E-04	YES	
BG071191	ESTs, Weakly similar to RIKEN cDNA 5730493B19 [Mus musculus] [M.musculus]		1.23	2.35	1.23E-05	YES	

BG071198	ESTs		1.32	2.50	2.79E-06	YES	
C87546	serine/threonine kinase 11	*Stk11*	1.94	3.83	6.21E-12	YES	protein modification
BG070317	DNA segment, Chr 7, ERATO Doi 680, expressed	*D7Ertd680e*	1.32	2.50	2.71E-06	YES	
BG070319	expressed sequence AU015581	*AU015581*	1.38	2.60	1.05E-06	YES	
BG070321	ESTs		1.43	2.69	4.15E-07	YES	
BG070059	ESTs, Weakly similar to RIKEN cDNA 5730493B19 [Mus musculus] [M.musculus]		1.09	2.13	1.11E-04	YES	
BG068215	expressed sequence AU022320	*AU022320*	1.11	2.16	7.98E-05	YES	
BG068964	ESTs		1.04	2.06	2.13E-04	YES	
BG067545	f-box and leucine-rich repeat protein 12	*Fbxl12*	1.51	2.86	7.95E-08	YES	ubiquitin pathway
BG068625	ESTs, Moderately similar to S12207 hypothetical protein (B2 element) - mouse [M.musculus]		1.39	2.62	8.14E-07	YES	
BG067561	expressed sequence C86727	*C86727*	1.55	2.94	3.57E-08	YES	
BG067565	expressed sequence C86753	*C86753*	1.81	3.50	1.51E-10	YES	
BG069687	DNA segment, Chr 16, ERATO Doi 780, expressed	*D16Ertd780e*	1.14	2.21	4.96E-05	YES	
BG070797	expressed sequence AU017169	*AU017169*	1.01	2.02	3.27E-04	YES	transport
BG070896	ESTs		1.30	2.46	4.14E-06	YES	
BG071173	ESTs		1.05	2.07	2.03E-04	YES	

BG070918	ESTs, Weakly similar to RIKEN cDNA 5730493B19 [Mus musculus] [M.musculus]		1.01	2.02	3.23E-04	YES	
BG071194	RAB27A, member RAS oncogene family	*Rab27a*	1.17	2.24	3.52E-05	YES	signal transducer
BG080727	nucleotide binding protein 1	*Nubp1*	-1.31	-2.47	3.56E-06	YES	unknown
BG080327	clathrin, light polypeptide (Lca)	*Clta*	-1.03	-2.05	2.42E-04	YES	transport
BG067273	ESTs		-1.06	-2.08	1.78E-04	YES	
BG068344	ESTs, Weakly similar to RIKEN cDNA 5730493B19 [Mus musculus] [M.musculus]		1.25	2.38	8.96E-06	YES	
BG080772	RIKEN cDNA 2510008M08 gene	*2510008M08Rik*	-1.12	-2.17	7.10E-05	YES	
BG080632	Scm-related gene containing four mbt domains	*Sfmbt*	-1.17	-2.25	3.30E-05	YES	
BG069797	expressed sequence AU020745	*AU020745*	-1.35	-2.55	1.59E-06	YES	
BG069818	ubiquitin specific protease 3	*Usp3*	-1.35	-2.55	1.69E-06	YES	ubiquitin pathway
BG069820	RIKEN cDNA 5330431K02 gene	*5330431K02Rik*	-1.45	-2.74	2.59E-07	YES	
BG069824	expressed sequence AU021092	*AU021092*	-1.21	-2.31	1.91E-05	YES	
BG069826	expressed sequence AW557704	*AW557704*	-1.71	-3.28	1.27E-09	YES	
BG069382	ESTs		-1.27	-2.41	6.94E-06	YES	
BG069844	expressed sequence C86045	*C86045*	-1.11	-2.16	8.18E-05	YES	
AU020960	proteaseome (prosome, macropain) 28 subunit, 3	*Psme3*	-1.27	-2.41	6.54E-06	YES	proteasome

BG071272			1.19	2.28	2.40E-05	YES	
BG069152			1.27	2.41	7.00E-06	YES	
BG070274	ESTs, Weakly similar to RIKEN cDNA 5730493B19 [Mus musculus] [M.musculus]		1.05	2.07	1.92E-04	YES	
BG070663	ESTs, Weakly similar to RIKEN cDNA 5730493B19 [Mus musculus] [M.musculus]		1.13	2.18	6.58E-05	YES	
BG080773	AMP deaminase 3	*Ampd3*	2.04	4.10	5.01E-13	YES	other
BG068351	DNA segment, Chr 3, ERATO Doi 552, expressed	*D3Ertd552e*	1.04	2.05	2.39E-04	YES	other
BG069803	ESTs, Weakly similar to RIKEN cDNA 5730493B19 [Mus musculus] [M.musculus]		1.10	2.14	9.47E-05	YES	
BG071246			1.63	3.10	7.41E-09	YES	
BG070195	expressed sequence AU015148	*AU015148*	1.01	2.02	3.21E-04	YES	
BG071273			1.37	2.58	1.22E-06	YES	
BG068757	Mus musculus, clone MGC:37615 IMAGE:4989784, mRNA, complete cds		1.03	2.04	2.64E-04	YES	
AU024687	ESTs		1.29	2.45	4.51E-06	YES	
BG082154	expressed sequence AU022238	*AU022238*	1.39	2.62	8.57E-07	YES	
BG068436	EST, Weakly similar to RIKEN cDNA 5730493B19 [Mus musculus] [M.musculus]		1.13	2.19	5.92E-05	YES	
BG069517	ESTs		1.50	2.82	1.09E-07	YES	

BG070969	RIKEN cDNA 2410081M15 gene	*2410081M15Rik*	1.42	2.68	4.38E-07	YES	unknown
BG071323			1.33	2.51	2.60E-06	YES	
BG071349	ESTs		1.19	2.29	2.26E-05	YES	
BG071355	ESTs, Weakly similar to I58401 protein-tyrosine kinase (EC 2.7.1.112) JAK3 - mouse [M.musculus]		1.39	2.62	7.98E-07	YES	
BG067062	ESTs, Moderately similar to S12207 hypothetical protein (B2 element) - mouse [M.musculus]		-1.59	-3.00	1.84E-08	YES	
BG080472	WD repeat domain 5	*Wdr5*	-1.59	-3.02	1.63E-08	YES	development
BG067088	DNA segment, Chr 2, ERATO Doi 391, expressed	*D2Ertd391e*	-1.22	-2.33	1.57E-05	YES	
BG081218	DNA cross-link repair 1A, PSO2 homolog (S. cerevisiae)	*Dclre1a*	-1.18	-2.27	2.67E-05	YES	DNA repair
BG081566			1.47	2.77	1.90E-07	YES	
AU021462	natural killer tumor recognition sequence	*Nktr*	-1.01	-2.02	3.28E-04	YES	chaperone
BG069650	expressed sequence AU020147	*AU020147*	-1.31	-2.48	3.44E-06	YES	
BG067912	ESTs, Weakly similar to I58401 protein-tyrosine kinase (EC 2.7.1.112) JAK3 - mouse [M.musculus]		1.02	2.03	2.82E-04	YES	
BG067918	B-cell translocation gene 2, anti-proliferative	*Btg2*	1.24	2.36	1.16E-05	YES	
BG069970	ESTs, Weakly similar to C17F3.3.p [Caenorhabditis elegans] [C.elegans]		1.20	2.29	2.23E-05	YES	

BG070788	ESTs		1.42	2.67	5.14E-07	YES	
BG070913	ESTs, Weakly similar to I58401 protein-tyrosine kinase (EC 2.7.1.112) JAK3 - mouse [M.musculus]		1.14	2.21	5.05E-05	YES	
BG071184	ESTs		1.16	2.23	3.94E-05	YES	
BG071206			1.57	2.96	2.72E-08	YES	
AU024060	ESTs		1.55	2.93	3.88E-08	YES	
BG068542	ESTs		1.06	2.09	1.69E-04	YES	
BG069255	ESTs		1.24	2.37	1.04E-05	YES	
BG069631	RIKEN cDNA 3110021P21 gene	*3110021P21Rik*	1.10	2.15	9.29E-05	YES	
BG070721	expressed sequence AU015738	*AU015738*	1.11	2.16	8.32E-05	YES	
BG070091	ESTs, Moderately similar to RIKEN cDNA 5730493B19 [Mus musculus] [M.musculus]		1.29	2.44	5.08E-06	YES	
BG083661	ESTs, Moderately similar to interleukin enhancer binding factor 3 [Mus musculus] [M.musculus]		1.04	2.06	2.19E-04	YES	
BG068227	ESTs, Moderately similar to I49130 reverse transcriptase - mouse [M.musculus]		1.02	2.03	2.91E-04	YES	
BG068953			1.00	2.00	3.86E-04	YES	
BG067527			1.86	3.64	3.87E-11	YES	
BG067898			1.26	2.40	7.65E-06	YES	
BG068627	ESTs		1.06	2.09	1.65E-04	YES	
BG069001	ESTs		1.05	2.07	1.90E-04	YES	
C85471	programmed cell death 8	*Pdcd8*	1.43	2.69	3.94E-07	YES	apoptosis control
BG067553	ESTs		1.39	2.63	7.74E-07	YES	

BG068650			1.17	2.26	3.13E-05	YES	
AU040159	Mus musculus, Similar to KIAA0494 gene product, clone IMAGE:4037144, mRNA, partial cds		1.11	2.16	7.86E-05	YES	
BG071179	ESTs, Moderately similar to S12207 hypothetical protein (B2 element) - mouse [M.musculus]		1.33	2.52	2.21E-06	YES	
AU014844	ESTs		1.42	2.68	4.58E-07	YES	
BG071203	ESTs		1.47	2.77	1.77E-07	YES	
BG067648			1.30	2.46	4.29E-06	YES	
BG067673	ESTs, Weakly similar to S33477 hypothetical protein 1 - rat (fragment) [R.norvegicus]		-1.12	-2.17	7.42E-05	YES	
BG084144	stromal antigen 1	*Stag1*	1.15	2.21	4.89E-05	YES	cell cycle
BG068042	DNA segment, Chr 2, ERATO Doi 501, expressed	*D2Ertd501e*	1.15	2.21	4.89E-05	YES	
BG069140			1.70	3.26	1.48E-09	YES	
BG067368			1.27	2.42	6.29E-06	YES	
AU021923	jagged 1	*Jag1*	1.38	2.61	9.07E-07	YES	development
BG067764			1.23	2.34	1.36E-05	YES	
BG069904	ESTs, Weakly similar to RIKEN cDNA 5730493B19 [Mus musculus] [M.musculus]		1.02	2.03	2.91E-04	YES	
BG069046	ESTs, Weakly similar to apoptotic chromatin condensation inducer in the nucleus [Mus musculus] [M.musculus]		1.44	2.72	3.03E-07	YES	

BG067247			1.50	2.83	9.76E-08	YES	
BG080332			1.97	3.92	2.67E-12	YES	
BG081752			1.14	2.20	5.62E-05	YES	
BG080372			1.08	2.11	1.29E-04	YES	
BG071222	ESTs, Weakly similar to RIKEN cDNA 5730493B19 [Mus musculus] [M.musculus]		1.48	2.80	1.43E-07	YES	
BG071237	ESTs, Highly similar to hypothetical protein FLJ10462 [Homo sapiens] [H.sapiens]		1.20	2.30	2.11E-05	YES	
BG084145	Hermansky-Pudlak syndrome 4 homolog (human)	*Hps4*	1.09	2.13	1.07E-04	YES	
C86021	growth differentiation factor 9	*Gdf9*	1.85	3.61	5.14E-11	YES	growth
C87237	expressed sequence C87237	*C87237*	1.08	2.11	1.33E-04	YES	
BG068428	ESTs		1.08	2.12	1.21E-04	YES	
BG067367	expressed sequence C86090	*C86090*	1.32	2.49	2.98E-06	YES	
BG069183	ESTs, Weakly similar to RIKEN cDNA 5730493B19 [Mus musculus] [M.musculus]		1.20	2.29	2.18E-05	YES	transport
BG068455	ESTs		1.01	2.01	3.52E-04	YES	
BG069189	ESTs		1.10	2.14	1.01E-04	YES	
AU040688			1.05	2.06	2.07E-04	YES	
BG071316	ESTs, Highly similar to hypothetical protein FLJ10462 [Homo sapiens] [H.sapiens]		1.28	2.43	5.55E-06	YES	
AU015471	RIKEN cDNA 2610529C04 gene	*2610529C04Rik*	1.05	2.07	1.99E-04	YES	transport

BG071357	ESTs, Weakly similar to RIKEN cDNA 5730493B19 [Mus musculus] [M.musculus]		1.48	2.79	1.48E-07	YES	
BG072392	ESTs, Weakly similar to RIKEN cDNA 5730493B19 [Mus musculus] [M.musculus]		1.23	2.34	1.38E-05	YES	
BG071395			1.13	2.19	6.15E-05	YES	
AW548258	procollagen-proline, 2-oxoglutarate 4-dioxygenase (proline 4-hydroxylase), alpha 1 polypeptide	*P4ha1*	1.07	2.09	1.57E-04	YES	other
BG074391	Zinc finger protein 212	*Znf212*	-1.07	-2.10	1.52E-04	YES	
AU041552			1.29	2.44	4.85E-06	YES	
AW546889			-1.40	-2.64	6.53E-07	YES	
BG084240	RIKEN cDNA 1010001M04 gene	*1010001M04Rik*	1.17	2.25	3.28E-05	YES	unknown
BG071376	ESTs, Weakly similar to RIKEN cDNA 5730493B19 [Mus musculus] [M.musculus]		1.30	2.47	3.81E-06	YES	
BG072412	ESTs, Weakly similar to I58401 protein-tyrosine kinase (EC 2.7.1.112) JAK3 - mouse [M.musculus]		1.24	2.37	1.03E-05	YES	
BG072414			1.60	3.03	1.46E-08	YES	
BG071380			1.45	2.73	2.71E-07	YES	
BG087300	RIKEN cDNA 5730488L07 gene	*5730488L07Rik*	1.05	2.07	2.02E-04	YES	signal transducer
BG074724	ESTs		1.05	2.08	1.83E-04	YES	
BG075055	ESTs		1.24	2.35	1.18E-05	YES	
AW551387			1.36	2.57	1.42E-06	YES	

BG072149	ESTs, Weakly similar to S26689 hypothetical protein hc1 - mouse (fragment) [M.musculus]		1.24	2.37	1.04E-05	YES	
AU042966	Mus musculus, Similar to waclaw, clone IMAGE:5030191, mRNA		1.14	2.20	5.41E-05	YES	
BG072508	ESTs		1.06	2.08	1.73E-04	YES	
AW547111			1.37	2.58	1.25E-06	YES	
AU043032			1.14	2.20	5.57E-05	YES	
BG072525	ESTs, Moderately similar to S12207 hypothetical protein (B2 element) - mouse [M.musculus]		1.19	2.29	2.32E-05	YES	
BG073476	ESTs, Weakly similar to I58401 protein-tyrosine kinase (EC 2.7.1.112) JAK3 - mouse [M.musculus]		1.02	2.02	3.14E-04	YES	
BG074764	ESTs		1.00	2.00	3.82E-04	YES	
BG075073	thymosin, beta 4, X chromosome	*Tmsb4x*	1.09	2.13	1.11E-04	YES	cytoskeleton
BG086831	programmed cell death 4	*Pdcd4*	1.09	2.13	1.11E-04	YES	apoptosis control
BG074774	ESTs		1.14	2.20	5.44E-05	YES	
BG075098	ESTs, Weakly similar to hypothetical protein FLJ12547 [Homo sapiens] [H.sapiens]		1.34	2.52	2.17E-06	YES	
BG087417			2.19	4.55	8.88E-15	YES	
BG075125	ESTs		1.01	2.01	3.51E-04	YES	
BG087125	nuclear receptor subfamily 0, group B, member 2	*Nr0b2*	1.00	2.00	3.90E-04	YES	signal transducer

a NCBI GenBank database (http://www.ncbi.nlm.nih.gov/) was used to obtain gene name, gene symbol, and summary function

Table S3 Changes in Gene Expression Associated with MAM and HN2 Treatment in Cultured Mouse Neurons*

			MAM/Control				HN2/Control				
Accession ID	Gene Name	Gene Symbol	Ratio (Log2)	Fold Change	unadjusted p (z test using pooled error estimate)	Statistically significant (adjusted for multiple comparisons)	Ratio (Log2)	Fold Change	unadjusted p (z test using pooled error estimate)	Statistically significant (adjusted for multiple comparisons)	Summary Function
BG069826	expressed sequence AW557704	*AW557704*	-1.52	-2.88	7.99E-10	YES	-1.71	-3.28	1.275E-09	YES	
BG075875	ESTs		-1.61	-3.05	9.92E-09	YES	-1.70	-3.26	4.323E-10	YES	
BG080472	WD repeat domain 5	*Wdr5*	-1.64	-3.11	4.24E-11	YES	-1.59	-3.02	1.625E-08	YES	development
BG067062	ESTs, Moderately similar to S12207 hypothetical protein (B2 element) - mouse [M.musculus]		-1.36	-2.56	4.53E-08	YES	-1.59	-3.00	1.838E-08	YES	
BG065423	ESTs		-1.06	-2.09	1.90E-05	YES	-1.54	-2.90	5.151E-08	YES	
AI848921	ESTs		-1.51	-2.85	7.26E-08	YES	-1.51	-2.84	3.399E-08	YES	
BG069820	RIKEN cDNA 5330431K02 gene	*5330431K02 Rik*	-1.37	-2.58	3.57E-08	YES	-1.45	-2.74	2.595E-07	YES	
AW546889			-1.01	-2.02	4.25E-05	YES	-1.40	-2.64	6.528E-07	YES	
AI846978	leucine rich repeat (in FLII) interacting protein 2	*Lrrfip2*	-1.56	-2.96	2.48E-08	YES	-1.39	-2.62	3.58E-07	YES	contains DNA repair motif
BG069818	ubiquitin specific protease 3	*Usp3*	-1.28	-2.43	2.27E-07	YES	-1.35	-2.55	1.691E-06	YES	ubiquitin pathway
BG075881	tyrosine 3-monooxygenase/tryptophan 5-monooxygenase activation protein, zeta polypeptide	*Ywhaz*	-1.31	-2.48	3.07E-06	YES	-1.29	-2.44	2.284E-06	YES	cell adhesion
AI851386	EST		-1.41	-2.65	5.49E-07	YES	-1.27	-2.40	3.533E-06	YES	
AI841920	expressed sequence AL022641	*AL022641*	-1.38	-2.60	9.43E-07	YES	-1.25	-2.37	4.853E-06	YES	transport
BG088094	succinate-Coenzyme A ligase, GDP-forming, beta subunit	*Suclg2*	-1.44	-2.72	2.71E-07	YES	-1.23	-2.35	6.106E-06	YES	mitochondria
AI851062	RIKEN cDNA 1300009F09 gene	*1300009F09 Rik*	-1.17	-2.25	2.99E-05	YES	-1.21	-2.31	9.909E-06	YES	transport
BG069824	expressed sequence AU021092	*AU021092*	-1.05	-2.07	2.33E-05	YES	-1.21	-2.31	1.913E-05	YES	
AI413648	ESTs		-1.25	-2.39	7.84E-06	YES	-1.19	-2.28	1.272E-05	YES	
BG081218	DNA cross-link repair 1A, PSO2 homolog (S. cerevisiae)	*Dclre1a*	-1.09	-2.13	1.13E-05	YES	-1.18	-2.27	2.672E-05	YES	DNA repair
BG075844	ESTs		-1.30	-2.46	3.83E-06	YES	-1.18	-2.26	1.544E-05	YES	
BG080632	Scm-related gene containing four mbt domains	*Sfmbt*	-1.16	-2.23	2.88E-06	YES	-1.17	-2.25	3.304E-05	YES	
AI854856	ESTs		-1.09	-2.13	1.01E-04	YES	-1.13	-2.20	3.207E-05	YES	
BG085385	hypothetical protein MGC37079	*MGC37079*	-1.29	-2.45	1.86E-07	YES	-1.13	-2.19	6.254E-05	YES	
BG075859	Nedd4 WW binding protein 4	*N4wbp4-pending*	-1.21	-2.31	1.63E-05	YES	-1.12	-2.18	3.827E-05	YES	unknown
AI847514	solute carrier family 1, member 3	*Slc1a3*	-1.52	-2.86	6.57E-08	YES	-1.12	-2.17	4.336E-05	YES	transport

AI842820	RIKEN cDNA 5730456K23 gene	*5730456K23 Rik*	-1.20	-2.30	1.83E-05	YES	-1.09	-2.13	6.628E-05	YES	
BG088092	solute carrier family 14 (urea transprorter), member 1	*Slc14a1*	-1.43	-2.70	3.24E-07	YES	-1.07	-2.10	8.568E-05	YES	transport
AI842684	interferon regulatory factor 3	*Irf3*	-1.00	-2.01	3.43E-04	YES	-1.07	-2.10	8.875E-05	YES	transcription regulator
AI847521	ESTs		-1.33	-2.51	2.27E-06	YES	-1.06	-2.09	9.889E-05	YES	
BG075824	ESTs, Weakly similar to S24407 formin isoform IV - mouse [M.musculus]		-1.19	-2.28	2.37E-05	YES	-1.04	-2.05	0.0001458	YES	
AI327246	ESTs, Weakly similar to S20900 titin - mouse (fragment) [M.musculus]		-1.15	-2.21	4.36E-05	YES	-1.01	-2.02	0.0002028	YES	
AI414793	RIKEN cDNA 2010003K11 gene	*2010003K11 Rik*	-1.10	-2.14	8.82E-05	YES	-1.01	-2.01	0.0002194	YES	
AI836925	G3P_MOUSE Glyceraldehyde 3-phosphate dehydrogenase (GAPDH) [M.musculus]		1.53	2.89	4.75E-08	YES	1.01	2.01	0.0002201	YES	
AI836199	protein kinase, cAMP dependent regulatory, type I, alpha	*Prkar1a*	1.80	3.47	1.54E-10	YES	1.01	2.02	0.0002118	YES	development
AI850252	RIKEN cDNA 6720463E02 gene	*6720463E02 Rik*	1.06	2.08	1.62E-04	YES	1.01	2.02	0.0002037	YES	other
AI835325	kelch-like ECH-associated protein 1	*Keap1*	1.27	2.40	6.49E-06	YES	1.01	2.02	0.0002023	YES	transcription regulator
AI845861	syntaxin 6	*Stx6*	1.70	3.24	1.47E-09	YES	1.01	2.02	0.0002019	YES	transport
AI838353	Microtubule-associated protein 1B (MAP 1B) (MAP1.2) (MAP1(X)) [Contains: MAP1 light chain LC1] [M.musculus]		1.31	2.47	3.25E-06	YES	1.02	2.02	0.0001992	YES	
AI843597	RIKEN cDNA 4921519L13 gene	*4921519L13 Rik*	1.52	2.86	6.64E-08	YES	1.02	2.03	0.0001805	YES	
AI853442	RIKEN cDNA 4930404J24 gene	*4930404J24 Rik*	1.33	2.52	2.08E-06	YES	1.02	2.03	0.0001795	YES	
AI846484	DNA segment, Chr 1, ERATO Doi 396, expressed	*D1Ertd396e*	1.61	3.06	9.09E-09	YES	1.03	2.04	0.0001659	YES	
AI835607			1.36	2.57	1.19E-06	YES	1.03	2.04	0.0001593	YES	
AI837434	RIKEN cDNA 4933428G20 gene	*4933428G20 Rik*	1.39	2.62	7.41E-07	YES	1.03	2.05	0.000154	YES	
AI835726	RIKEN cDNA 1810060D16 gene	*1810060D16 Rik*	1.20	2.30	1.81E-05	YES	1.03	2.05	0.0001505	YES	transcription
AI841802	seryl-aminoacyl-tRNA synthetase 1	*Sars1*	1.42	2.67	4.24E-07	YES	1.04	2.05	0.0001473	YES	other
AI840337			1.11	2.16	7.26E-05	YES	1.04	2.05	0.0001407	YES	
AI852095	RIKEN cDNA 5730536A07 gene	*5730536A07 Rik*	1.70	3.25	1.34E-09	YES	1.04	2.06	0.0001348	YES	
AI837029	reticulocalbin	*Rcn*	1.74	3.35	5.13E-10	YES	1.05	2.07	0.0001189	YES	
AI837600	expressed sequence AI850305	*AI850305*	1.54	2.92	3.74E-08	YES	1.05	2.07	0.0001182	YES	
AI850771	EST		1.41	2.65	5.43E-07	YES	1.05	2.07	0.000118	YES	
AI845485	four and a half LIM domains 4	*Fhl4*	1.40	2.65	5.59E-07	YES	1.05	2.07	0.0001178	YES	transport

AI842099	protein kinase C, alpha	*Prkca*	1.59	3.01	1.48E-08	YES	1.05	2.08	0.0001136	YES	signal transducer
BG075908	epithelial protein lost in neoplasm	*Eplin-pending*	1.56	2.95	2.67E-08	YES	1.05	2.08	0.0001126	YES	unknown
AI842511			1.85	3.60	4.45E-11	YES	1.06	2.08	0.0001079	YES	
AI839804	CDC-like kinase 2	*Clk2*	1.01	2.02	3.09E-04	YES	1.06	2.08	0.0001054	YES	other
AI838366	bromodomain-containing 4	*Brd4*	1.42	2.67	4.43E-07	YES	1.06	2.09	0.0001011	YES	unknown
AI843621	expressed sequence AI836659	*AI836659*	1.76	3.38	3.73E-10	YES	1.06	2.09	9.8E-05	YES	
AI844569	RIKEN cDNA 1100001J08 gene	*1100001J08Rik*	1.07	2.10	1.44E-04	YES	1.07	2.09	9.343E-05	YES	
AI850099	RIKEN cDNA 2900002L20 gene	*2900002L20Rik*	1.09	2.13	9.86E-05	YES	1.07	2.11	8.236E-05	YES	unknown
AI845582	sparc/osteonectin, cwcv and kazal-like domains proteoglycan 1	*Spock1*	1.67	3.18	2.76E-09	YES	1.07	2.11	8.205E-05	YES	unknown
AI837996	GRP1 (general receptor for phosphoinositides 1)-associated scaffold protein	*Grasp*	1.61	3.04	1.07E-08	YES	1.08	2.11	7.911E-05	YES	signal transducer
AI837602	EST		1.65	3.13	4.37E-09	YES	1.08	2.12	7.335E-05	YES	
AI842821	phospholipase C-like 2	*Plcl2*	1.10	2.15	8.55E-05	YES	1.08	2.12	7.186E-05	YES	signal transducer
AI839125	RIKEN cDNA 3100001N19 gene	*3100001N19Rik*	1.32	2.50	2.44E-06	YES	1.08	2.12	7.17E-05	YES	protein synthesis
AI852836	RIKEN cDNA 5530600M07 gene	*5530600M07Rik*	1.10	2.14	9.33E-05	YES	1.08	2.12	7.107E-05	YES	
AI835779	Basigin precursor (Basic immunoglobulin superfamily) (Membrane glycoprotein GP42) [M.musculus]		1.86	3.64	3.23E-11	YES	1.09	2.12	6.9E-05	YES	
AI847692	EST		1.06	2.08	1.69E-04	YES	1.09	2.13	6.672E-05	YES	
AI839134	eukaryotic translation initiation factor 3, subunit 3 (gamma, 40kD)	*Eif3s3*	1.44	2.72	2.65E-07	YES	1.09	2.13	6.293E-05	YES	protein synthesis
AI839850	ESTs		1.60	3.04	1.10E-08	YES	1.09	2.14	6.066E-05	YES	
AI839797	RIKEN cDNA 1700008C22 gene	*1700008C22Rik*	1.36	2.57	1.24E-06	YES	1.10	2.14	5.693E-05	YES	
AI838959	actin, alpha 2, smooth muscle, aorta	*Acta2*	1.36	2.56	1.38E-06	YES	1.10	2.14	5.522E-05	YES	cytoskeleton organization
AI845440	RIKEN cDNA C330007D15 gene	*C330007D15Rik*	1.39	2.62	7.10E-07	YES	1.11	2.16	4.872E-05	YES	
AI841636	RIKEN cDNA 1810021J13 gene	*1810021J13Rik*	1.22	2.33	1.43E-05	YES	1.11	2.16	4.735E-05	YES	unknown
AI843291	synbindin	*Sbdn*	1.27	2.41	5.93E-06	YES	1.11	2.16	4.732E-05	YES	transport
AI843538	ESTs		1.52	2.87	6.03E-08	YES	1.11	2.16	4.662E-05	YES	
AI839988	hypothetical protein MGC38419	*MGC38419*	1.47	2.77	1.67E-07	YES	1.12	2.17	4.259E-05	YES	transport
AI841881	RIKEN cDNA 2410008M22 gene	*2410008M22Rik*	1.92	3.79	7.71E-12	YES	1.12	2.17	4.151E-05	YES	
AI849136	EST		1.07	2.10	1.41E-04	YES	1.12	2.17	4.118E-05	YES	
AI839755	insulin receptor substrate 1	*Irs1*	1.77	3.40	3.03E-10	YES	1.12	2.18	3.935E-05	YES	signal transducer

AI839520	guanylate kinase 1	*Guk1*	1.63	3.10	6.04E-09	YES	1.12	2.18	3.842E-05	YES	other
BG076138	ESTs		1.87	3.67	2.41E-11	YES	1.12	2.18	3.759E-05	YES	
AI835378	ESTs		1.33	2.52	1.97E-06	YES	1.13	2.18	3.645E-05	YES	
AI835905	ferritin heavy chain	*Fth*	1.40	2.63	6.62E-07	YES	1.13	2.19	3.577E-05	YES	transport
AI841630	ATP citrate lyase	*Acly*	1.33	2.51	2.26E-06	YES	1.13	2.19	3.565E-05	YES	other
AI850305	expressed sequence AI850305	*AI850305*	1.15	2.22	4.06E-05	YES	1.13	2.19	3.554E-05	YES	
BG076200	DAZ associated protein 1	*Dazap1*	1.27	2.41	5.95E-06	YES	1.13	2.19	3.526E-05	YES	mitochondria
AI854434	ribosomal protein L13a	*Rpl13a*	2.25	4.75	1.11E-15	YES	1.13	2.19	3.47E-05	YES	development
AI835645	formin binding protein 4	*Fnbp4*	1.66	3.16	3.31E-09	YES	1.13	2.19	3.355E-05	YES	
AI843553	heat shock 70kD protein 5 (glucose-regulated protein, 78kD)	*Hspa5*	1.64	3.11	5.47E-09	YES	1.15	2.21	2.698E-05	YES	heat shock response
AI843335	expressed sequence AU040105	*AU040105*	1.04	2.06	2.03E-04	YES	1.15	2.22	2.528E-05	YES	
AI839857	similar to hypothetical protein FLJ12949	*LOC215194*	1.26	2.39	7.39E-06	YES	1.15	2.22	2.464E-05	YES	
AI844003	RIKEN cDNA 4930566A11 gene	*4930566A11Rik*	1.66	3.15	3.62E-09	YES	1.16	2.23	2.182E-05	YES	
AI841241	galactosidase, beta 1	*Glb1*	1.76	3.38	3.98E-10	YES	1.16	2.24	2.075E-05	YES	other
AI843248	RIKEN cDNA 5730405I09 gene	*5730405I09Rik*	1.83	3.55	7.13E-11	YES	1.17	2.24	1.916E-05	YES	cell cycle
AI837371	RIKEN cDNA 1810005K14 gene	*1810005K14Rik*	1.89	3.72	1.50E-11	YES	1.17	2.25	1.853E-05	YES	
AI850599	transcriptional regulating protein 132, isoform 2; rapa-2; rapa-1 [Homo sapiens] [H.sapiens]		1.05	2.07	1.83E-04	YES	1.17	2.25	1.803E-05	YES	
AI836940	EST		1.51	2.84	8.12E-08	YES	1.17	2.25	1.757E-05	YES	
AI838922	EST		1.84	3.59	5.20E-11	YES	1.18	2.27	1.477E-05	YES	
AI838727	EST		1.60	3.03	1.17E-08	YES	1.18	2.27	1.454E-05	YES	
AI840241			1.56	2.95	2.78E-08	YES	1.18	2.27	1.413E-05	YES	
BG072622	ESTs		1.06	2.09	1.84E-05	YES	1.18	2.27	2.643E-05	YES	
AI836953			1.61	3.06	8.87E-09	YES	1.19	2.28	1.341E-05	YES	
AI841880	endothelial cell-selective adhesion molecule	*Esam-pending*	1.53	2.90	4.64E-08	YES	1.19	2.29	1.23E-05	YES	cell adhesion
AI838008	glyoxylate reductase/hydroxypyruvate reductase	*Grhpr*	1.73	3.32	6.75E-10	YES	1.20	2.30	1.095E-05	YES	other
AI842822	RIKEN cDNA 1110003F06 gene	*1110003F06Rik*	1.94	3.84	4.64E-12	YES	1.20	2.30	1.084E-05	YES	
AI839739	EST, Weakly similar to TBA1_MOUSE Tubulin alpha-1 chain [M.musculus]		1.49	2.80	1.15E-07	YES	1.20	2.30	1.083E-05	YES	
AI836538	EST		1.47	2.77	1.63E-07	YES	1.21	2.31	9.15E-06	YES	
AI838773	expressed sequence AI838773	*AI838773*	1.62	3.07	7.87E-09	YES	1.22	2.32	8.402E-06	YES	
AI839330	neuron specific gene family member 1	*Nsg1*	1.34	2.54	1.70E-06	YES	1.22	2.32	8.321E-06	YES	neurogenesis
AI836605	inhibitor of growth family, member 4	*Ing4*	1.97	3.90	2.54E-12	YES	1.22	2.32	8.208E-06	YES	transcription regulator
AI845521	myosin VIIA and Rab interacting protein	*Myrip*	1.62	3.08	7.37E-09	YES	1.22	2.33	7.987E-06	YES	transport
AI838763	expressed sequence AI838763	*AI838763*	1.41	2.65	5.46E-07	YES	1.24	2.36	5.424E-06	YES	

AI835993	EST		1.79	3.46	1.84E-10	YES	1.24	2.37	5.313E-06	YES	
AI845532	related gene family, member C (S. cerevisiae), clone IMAGE:4950601, mRNA, partial cds		1.72	3.29	9.59E-10	YES	1.24	2.37	5.168E-06	YES	other
AI840425	EST		1.70	3.25	1.41E-09	YES	1.24	2.37	5.103E-06	YES	
AI840113	peroxiredoxin 2	*Prdx2*	1.71	3.27	1.08E-09	YES	1.25	2.38	4.672E-06	YES	apoptosis control
AI843670	RIKEN cDNA A230106A15 gene	*A230106A15 Rik*	1.83	3.56	7.03E-11	YES	1.25	2.38	4.638E-06	YES	
AI848056	RIKEN cDNA 2700079K05 gene	*2700079K05 Rik*	1.29	2.45	4.05E-06	YES	1.25	2.38	4.461E-06	YES	
AI838946	Mus musculus, clone IMAGE:4527484, mRNA, partial cds		1.61	3.05	9.57E-09	YES	1.26	2.39	4.239E-06	YES	
AI839628			1.77	3.41	2.86E-10	YES	1.26	2.39	3.963E-06	YES	
AI846342	membrane-type frizzled-related protein	*Mfrp*	1.37	2.58	1.15E-06	YES	1.26	2.39	3.936E-06	YES	development
AI840807	beta-spectrin 4	*Spnb4*	1.84	3.59	5.09E-11	YES	1.26	2.40	3.854E-06	YES	scytoskeleton
AI414844	RIKEN cDNA 2310035L15 gene	*2310035L15 Rik*	1.31	2.48	3.18E-06	YES	1.26	2.40	3.718E-06	YES	
AI838564	expressed sequence AL024016	*AL024016*	1.20	2.30	1.91E-05	YES	1.27	2.41	3.383E-06	YES	
AI845823	(protein for IMAGE:3610257), clone MGC:37326 IMAGE:4975562, mRNA, complete cds		1.73	3.32	6.87E-10	YES	1.27	2.41	3.268E-06	YES	
AI835639	ESTs		1.28	2.43	5.22E-06	YES	1.28	2.42	2.835E-06	YES	
AI849361	RIKEN cDNA 1700056O17 gene	*1700056O17 Rik*	1.72	3.29	9.24E-10	YES	1.29	2.44	2.398E-06	YES	
AI843257	hypothetical protein MGC36831	*MGC36831*	1.60	3.04	1.14E-08	YES	1.29	2.45	2.159E-06	YES	
AW556086			1.15	2.21	4.45E-05	YES	1.29	2.45	2.081E-06	YES	
AI836589	ATP synthase, H+ transporting mitochondrial F1 complex, beta subunit	*Atp5b*	1.34	2.54	1.68E-06	YES	1.30	2.46	1.98E-06	YES	transport
AI836010	RIKEN cDNA 1110013B16 gene	*1110013B16 Rik*	2.10	4.30	6.75E-14	YES	1.30	2.46	1.898E-06	YES	unknown
AI836047	ESTs		1.60	3.03	1.27E-08	YES	1.30	2.47	1.778E-06	YES	
AI840275	protein COII [Mus musculus] [M.musculus]		1.58	2.98	1.93E-08	YES	1.31	2.47	1.669E-06	YES	
AI834863	ribosomal protein L26	*Rpl26*	1.81	3.50	1.18E-10	YES	1.31	2.48	1.532E-06	YES	protein synthesis
AI839010			1.43	2.69	3.66E-07	YES	1.32	2.49	1.403E-06	YES	
AI845510	ESTs		2.15	4.45	1.71E-14	YES	1.32	2.49	1.375E-06	YES	
AI837442			1.71	3.28	1.04E-09	YES	1.32	2.50	1.255E-06	YES	
AI843781	protein phosphatase 3, catalytic subunit, alpha isoform	*Ppp3ca*	2.09	4.25	1.01E-13	YES	1.32	2.50	1.217E-06	YES	
AI840919	DNA-damage inducible transcript 3	*Ddit3*	1.45	2.74	2.24E-07	YES	1.33	2.51	1.134E-06	YES	apoptosis control
AI840347	EST		1.95	3.87	3.36E-12	YES	1.33	2.52	1.034E-06	YES	
BG088348	cDNA sequence BC032200	*BC032200*	1.22	2.33	1.36E-05	YES	1.33	2.52	1.001E-06	YES	
BG076127	expressed sequence AU021063	*AU021063*	1.25	2.37	8.99E-06	YES	1.34	2.52	9.789E-07	YES	
AI851852	ESTs		1.76	3.39	3.46E-10	YES	1.34	2.53	9.485E-07	YES	

AI852958	ESTs, Weakly similar to pregnancy upregulated non-ubiquitously expressed CaM kinase; calcium/calmodulin-dependent protein kinase 1, beta [Mus musculus] [M.musculus]		1.31	2.48	3.05E-06	YES	1.34	2.53	8.889E-07	YES	
AI840047	EST		1.58	2.98	1.95E-08	YES	1.34	2.53	8.776E-07	YES	
AI843570	ESTs		1.65	3.13	4.59E-09	YES	1.35	2.54	8.082E-07	YES	
AI841643	polypeptide	*Pdgfb*	1.59	3.01	1.51E-08	YES	1.35	2.54	8.051E-07	YES	cell cycle
AI837380	hypothetical protein MGC36388	*MGC36388*	2.18	4.52	9.10E-15	YES	1.35	2.55	7.763E-07	YES	mitochondria
AI839815	EST		1.39	2.63	6.84E-07	YES	1.35	2.55	7.651E-07	YES	
AI845464	RIKEN cDNA 1700042O10 gene	*1700042O10 Rik*	2.14	4.40	2.69E-14	YES	1.35	2.55	7.463E-07	YES	
AI835656			1.18	2.27	2.59E-05	YES	1.35	2.56	6.984E-07	YES	
AI838600	EST		1.69	3.23	1.66E-09	YES	1.36	2.56	6.787E-07	YES	
AW556082	aladin	*LOC223921*	1.02	2.02	2.98E-04	YES	1.36	2.57	6.259E-07	YES	
AW555822			1.08	2.12	1.12E-04	YES	1.36	2.57	6.128E-07	YES	
AI836098	EST		1.57	2.97	2.21E-08	YES	1.37	2.58	5.579E-07	YES	
AI846795	EST		1.75	3.36	4.72E-10	YES	1.37	2.58	5.576E-07	YES	
AW559004			1.15	2.21	4.36E-05	YES	1.37	2.58	5.318E-07	YES	
AI840342	EST		1.67	3.18	2.63E-09	YES	1.37	2.58	5.183E-07	YES	
AI841447			1.14	2.21	4.78E-05	YES	1.38	2.60	4.339E-07	YES	
AI845870	EST		1.54	2.90	4.46E-08	YES	1.38	2.60	4.305E-07	YES	
AI836097	EST		1.45	2.73	2.36E-07	YES	1.38	2.61	4.044E-07	YES	
AW556391	ribosomal protein L32	*Rpl32*	1.20	2.30	1.84E-05	YES	1.39	2.62	3.657E-07	YES	protein synthesis
AI843286			1.69	3.23	1.71E-09	YES	1.39	2.62	3.562E-07	YES	
AI842597	transcription factor 4	*Tcf4*	1.76	3.39	3.38E-10	YES	1.39	2.63	3.248E-07	YES	development
AI837129	ESTs		1.90	3.74	1.26E-11	YES	1.39	2.63	3.24E-07	YES	
AI840852	EST		1.66	3.16	3.19E-09	YES	1.41	2.65	2.494E-07	YES	
AI846743	transgelin	*Tagln*	1.72	3.29	9.28E-10	YES	1.41	2.65	2.479E-07	YES	development
AI839634			1.56	2.95	2.56E-08	YES	1.42	2.67	2.114E-07	YES	
AI838940	RIKEN cDNA 1110057L18 gene	*1110057L18R ik*	1.98	3.94	1.81E-12	YES	1.42	2.67	2.038E-07	YES	
AI840334	EST, Weakly similar to beta-tubulin cofactor C; tubulin-specific chaperone c [Homo sapiens] [H.sapiens]		1.78	3.44	2.13E-10	YES	1.42	2.68	1.867E-07	YES	
AI854139	ESTs, Moderately similar to T00388 hypothetical protein KIAA0616 - human (fragment) [H.sapiens]		1.25	2.38	8.14E-06	YES	1.43	2.69	1.748E-07	YES	
AI846726	EST		1.92	3.78	7.83E-12	YES	1.43	2.70	1.482E-07	YES	
AI841315	EST		1.83	3.57	6.27E-11	YES	1.45	2.74	1.012E-07	YES	
BG088381	RIKEN cDNA 2010100O12 gene	*2010100O12 Rik*	1.17	2.25	3.03E-05	YES	1.45	2.74	9.893E-08	YES	unknown
AI845753	RIKEN cDNA 2810422J05 gene	*2810422J05 Rik*	1.97	3.92	2.16E-12	YES	1.46	2.74	9.401E-08	YES	

AI835315	Mus musculus, clone IMAGE:3586350, mRNA, partial cds		1.57	2.96	2.36E-08	YES	1.46	2.75	9.278E-08	YES	
AI841536			1.75	3.37	4.29E-10	YES	1.46	2.76	8.012E-08	YES	
AI841876	hemoglobin, beta adult minor chain	*Hbb-b2*	1.99	3.98	1.22E-12	YES	1.50	2.82	4.278E-08	YES	transport
AI845502	eukaryotic translation initiation factor 3	*Eif3*	2.29	4.89	4.44E-16	YES	1.50	2.83	4.004E-08	YES	protein synthesis
AI840835	EST		1.21	2.31	1.61E-05	YES	1.50	2.83	3.924E-08	YES	
AI837833	zinc finger protein 95	*Zfp95*	1.52	2.87	5.76E-08	YES	1.51	2.86	2.827E-08	YES	transcription regulator
AI844955			1.10	2.14	9.19E-05	YES	1.53	2.88	2.177E-08	YES	
AI842966	RIKEN cDNA 1110019I12 gene	*1110019I12Rik*	1.93	3.82	5.51E-12	YES	1.54	2.91	1.6E-08	YES	
AI842497	RIKEN cDNA 1810060J02 gene	*1810060J02 Rik*	1.73	3.32	6.90E-10	YES	1.54	2.91	1.582E-08	YES	
AI836541	ribosomal protein L19	*Rpl19*	1.94	3.85	4.38E-12	YES	1.54	2.91	1.578E-08	YES	protein synthesis
AI844129	cDNA sequence BC017158	*BC017158*	1.18	2.26	2.82E-05	YES	1.56	2.95	1.111E-08	YES	
AI840930	ESTs		1.94	3.83	5.05E-12	YES	1.56	2.95	1.108E-08	YES	
AI835847	ESTs, Highly similar to NUMM_MOUSE NADH-ubiquinone oxidoreductase 13 kDa-A subunit (Complex I-13KD-A) (CI-13KD-A) [M.musculus]		2.01	4.03	8.04E-13	YES	1.57	2.97	8.517E-09	YES	
AI840311	EST		1.98	3.94	1.83E-12	YES	1.58	2.99	7.364E-09	YES	
AI837935	EST		1.65	3.15	3.79E-09	YES	1.58	2.99	7.351E-09	YES	
AI836491	heat shock 10 kDa protein 1 (chaperonin 10)	*Hspe1*	2.01	4.03	7.99E-13	YES	1.58	2.99	7.114E-09	YES	chaperone
AI841812	expressed sequence AI043088	*AI043088*	1.94	3.83	5.13E-12	YES	1.58	3.00	6.605E-09	YES	
AI836814	EST		1.80	3.48	1.46E-10	YES	1.60	3.03	4.702E-09	YES	
AI838397	EST, Weakly similar to COP9 subunit 6 (MOV34 homolog, 34 kD) [Homo sapiens] [H.sapiens]		1.44	2.71	2.91E-07	YES	1.60	3.03	4.401E-09	YES	
AI837396	EST		1.84	3.57	5.92E-11	YES	1.62	3.08	2.76E-09	YES	
AW555902			1.37	2.58	1.13E-06	YES	1.63	3.10	2.288E-09	YES	
AI849487	EST		1.71	3.27	1.09E-09	YES	1.69	3.22	6.376E-10	YES	
AI837644	RIKEN cDNA 2900002K07 gene	*2900002K07 Rik*	2.26	4.78	8.88E-16	YES	1.74	3.34	1.901E-10	YES	unknown
AI841463	NK6 transcription factor related, locus 2 (Drosophila)	*Nkx6-2*	2.12	4.34	4.42E-14	YES	1.74	3.34	1.83E-10	YES	transcription regulator
AI848163	ESTs		1.80	3.47	1.58E-10	YES	1.74	3.34	1.804E-10	YES	
AI837370	EST, Weakly similar to JW0059 mtprd protein - mouse [M.musculus]		1.82	3.53	9.34E-11	YES	1.76	3.38	1.197E-10	YES	
AI840542			2.13	4.39	2.89E-14	YES	1.78	3.43	7.284E-11	YES	
AI836319	EST		2.12	4.36	3.75E-14	YES	1.79	3.45	6.02E-11	YES	
AI841883			2.06	4.16	2.43E-13	YES	1.79	3.45	5.809E-11	YES	
AI839548			1.98	3.94	1.90E-12	YES	1.80	3.48	4.341E-11	YES	
AI837859	RIKEN cDNA 1810036J22 gene	*1810036J22 Rik*	2.21	4.62	3.77E-15	YES	1.82	3.53	2.701E-11	YES	

AI845665		1.79	3.45	1.95E-10	YES	1.89	3.70	4.603E-12	YES	
AI837335	EST	1.78	3.42	2.53E-10	YES	1.94	3.85	1.033E-12	YES	
AI853386	EST	1.95	3.85	4.09E-12	YES	2.26	4.78	2.22E-16	YES	

a NCBI GenBank database (http://www.ncbi.nlm.nih.gov/) was used to obtain gene name, gene symbol, and summary function
